# From “More Is Different” to Algorithmic Emergence: Regularity, Compression, and the Limits of Discovery

**DOI:** 10.3390/e28101090

**Published:** 2026-10-07

**Authors:** Giulio Ruffini, Francesca Castaldo

**Affiliations:** 1 Barcelona Computational Foundation (BCOM), 08022 Barcelona, Spain; francesca.castaldo@bcom.one; 2 Brain Modeling Department, Neuroelectrics, 08035 Barcelona, Spain; 3 Starlab Barcelona SLU, 08035 Barcelona, Spain

**Keywords:** emergence, regularity, compression, algorithmic information theory, Kolmogorov complexity, model discovery, cognition, coarse-graining, uncomputability, algorithmic statistics, 68Q30, 94A15

## Abstract

Scientific discovery seeks regularities that support explanation and prediction. Compression makes their reuse explicit: shared structure is described once, while parameters specify individual cases. A model can, therefore, pay for itself through repeated use even when its description is not minimal, provided it captures reusable structure in the data. Because regularities are often easier to identify in simpler systems, a reductionist approach is often employed: discover the laws of the parts and treat them as fundamental. Yet knowing those laws does not by itself provide useful coarse-grained models of the larger systems they compose. Here we formulate Anderson’s distinction between reduction and construction for finite algorithmic observers and prove three barriers to such construction. First, an observer’s coarse-grained record may retain so much information about initial or boundary conditions that no substantially shorter description exists, even when the underlying laws are simple and known. Second, when a shorter description does exist, open-ended search can eventually find one, but there is no computable bound on how long this may take, and no algorithm that always halts with a valid description can succeed in every case. Third, every such algorithm has blind spots at all sufficiently large lengths: records it leaves unshortened even though they admit descriptions of only logarithmic length. Yet observers do discover useful models. We call the event in which an observer acquires a representation relevant to its objective and a reusable model that reveals previously unavailable regularity *algorithmic emergence*. We give a sufficient certificate in explicit code lengths: the pair passes when, with its own description cost included, it compresses the retained data relative to an agreed baseline and yields further savings on later observations. Even for a stream that repeats one block, no computable procedure that always halts with valid codes can guarantee finding a passing pair with the observations available whenever one exists, nor remain within a fixed number of bits of the best qualifying complete code. Favorable structure, including symmetry and restricted model classes, can nevertheless make discovery feasible.

## 1. Introduction: Reduction Is Not Construction

Scientific discovery seeks regularities that support succinct explanation and prediction across cases—compression and generalization. A model captures what the cases share; its parameters specify what changes from one case to another. The parameter sequence can itself have regularity, such as a recurring movement. Together, the model and jointly encoded parameters generate the modeled observations. They provide a compressed description when the cost of the model, the parameters, and whatever the model leaves unexplained is less than that of describing each case separately. The model’s fixed cost is paid once, so even a large implementation can yield savings through reuse. If the regularity persists in new observations, the same model supports prediction.

This role of compression in science and cognition is explicit in the *Kolmogorov Manifesto*, in the minimum message length and minimum description length principles, and in Li and Vitányi’s treatment of induction as compression [1,2,3,4,5]. Schmidhuber connects discovery to improvements in an agent’s ability to compress its observations, and recent work on language models develops the relation between prediction and lossless coding [6,7]. Prediction and compression are two uses of one regularity: under logarithmic loss a predictor’s loss is an ideal code length, and a deterministic predictor compresses a binary record by transmitting only the positions of its mistakes, provided these are few (Appendix B). Compression gives evidence that a model captures structure; reuse on new records tests whether that structure generalizes.

There is also a reason to expect short descriptions to generalize. Solomonoff’s universal predictor weights each program *p* by $2^{-|p|}$. For any computable data-generating source, this predictor’s expected cumulative excess logarithmic loss relative to that source is bounded by the source’s description complexity, up to fixed coding constants [8,9,10]. This is a predictive bound within the class of computable sources, not a proof that Nature is simple. Earlier KT work considered a simplicity bias in the structures encountered in the world [11], and related results show how such a bias arises in broad classes of low-complexity computable input–output maps [12].

Models guide the actions of agents in pursuit of their objectives. We write OF for the objective function that determines what matters to the observer. One important class is telehomeostatic agency, in which action is organized around regulation that supports continued persistence—of the agent, its lineage, or a larger system to which it contributes [13]. Modeling is instrumental rather than an end in itself: successful regulation requires the agent to capture reusable regularities in its world and in its own dynamics [1].

Among models that are comparably adequate, shorter effective descriptions have two advantages. They provide stronger inductive grounds for expecting the captured regularity to generalize, and they reduce the cost of storing and transmitting the model. But description length is not the only resource that matters. A longer implementation of the same functional regularity may be preferable if it produces predictions faster, uses less working memory, or requires less energy to run. Thus compression is evidence that reusable regularity has been captured, not the ultimate objective of the agent. None of these considerations, however, supplies a general procedure for discovering such regularities.

Furthermore, while compression is necessary for modeling, it is not sufficient. A model that captures reusable regularity can compress the observations it applies to through reuse, even when it is imperfect or far from minimal; its description cost need not yet have been repaid. The converse fails. A compressor of past data does not hand over a model: a short program for one record need not expose which part is shared structure and which part is the case, how the case is parametrized, or what to send when the next observation arrives. This is why the paper measures acquisition by compression and tests it by reuse. Reuse on new observations, with the same coordinates in the same roles, is what the certificate requires beyond a shorter code for the past.

Regularities are often easier to find in simpler systems. This is one motivation for reductionism: isolate smaller parts or fewer degrees of freedom, discover the laws that govern them, and treat those laws as the foundation for understanding composite systems. A microscopic theory is itself a compression success, often obtained in such a restricted setting. The question is whether that success transfers upward. Given the microscopic laws, the scientist would still need a procedure for discovering the theories of the larger systems those parts compose. In *More Is Different*, Anderson denied that the laws supply one:

“The ability to reduce everything to simple fundamental laws does not imply the ability to start from those laws and reconstruct the universe.”

[14]

Here, we take “reconstructing” to mean finding the higher-level variables, organizing principles, and effective theories that make the behavior of a larger system intelligible—in other words, compressible. Compression is the part of intelligibility this paper measures; explanatory or causal adequacy requires further conditions (Section 4.2).

Given a microscopic law and the information specifying a particular case, direct simulation can generate its observed history. We call this *generative reduction*. The stronger *constructionist converse* would provide a useful higher-level representation and reusable model. A simulated trajectory does not by itself supply these. A tornado, for example, is understood through variables such as location, rotation, intensity, and expected evolution, rather than a list of every molecule. Whether the resulting macromodel also provides a computational shortcut is a separate question.

Throughout, “microscopic” and “macroscopic” are relative terms. A microscopic description is a set of variables together with a law that updates them; a macroscopic description is another set of variables obtained from the first by a projection. Nothing here requires the microscopic level to be fundamental physics, and nothing requires the projection to be an average. A cell’s chemistry is microscopic for its physiology, and a phase label or a single halting bit can be a macroscopic variable. Any level can play the microscopic role for the level built on it.

The choice of projection also fixes what the observer must then model. Under reversible microscopic dynamics, the complete-state algorithmic information is conserved once the law and the elapsed time are supplied, but information held initially in the omitted detail can later reach the observed variables. A projection that makes one snapshot simple can, therefore, leave a complicated history, or discard the distinctions needed to predict how the retained variables evolve. Section 6 gives this accounting and its consequences for choosing ρ.

Within Kolmogorov Theory (KT), we take *algorithmic emergence* to be an event in an observer’s descriptive history: the acquisition of a useful, reusable model under which the observer’s retained observations admit a shorter description than it could give before. A pattern is what such a model captures; emergence lies in its discovery, without changing the complexity of the fixed record.

This view aligns with Bédard and Bergeron’s account of emergence in theory space, where new partial models yield discrete gains in description  [15]. Our emphasis is on the observer’s acquisition of a usable model and on whether a procedure can guarantee such a transition. The representation may already be supplied; the acquisition concerns a newly available description.

We study a finite algorithmic version of this construction problem:

**Q1.**  *Existence.* Does the specified observed history admit a substantial lossless shortening relative to its literal code?
**Q2.**  *Discovery.* If any shortening exists, can one general terminating procedure always find a valid one?
**Q3.**  *Near-optimality.* Can such a procedure always stay within a fixed number of bits of the shortest description?

Three barriers answer these questions. Simple known dynamics can preserve almost all the initial-condition information in the observed history, so no substantial shortening exists (the *residual-information* barrier, Theorem 1). No algorithm that always halts with a valid description finds a shortening whenever one exists (the *discovery* barrier, Theorem 2). No such algorithm stays within a fixed number of bits of the shortest description (the *optimality* barrier, Theorem 3), and each leaves records with logarithmic-length descriptions unshortened at every sufficiently large length (Proposition 1).

The same barriers reach model discovery. Suppose the observations simply repeat one block, and a constructor must return a representation and a reusable model from the first copy. Every such constructor leaves some block unshortened; that block nevertheless has a short description, one that names it through the constructor’s own failure and also predicts its repetitions. A  qualifying pair, therefore, exists that the constructor, with the observations available, cannot return (Proposition 2).

**Figure 1:**
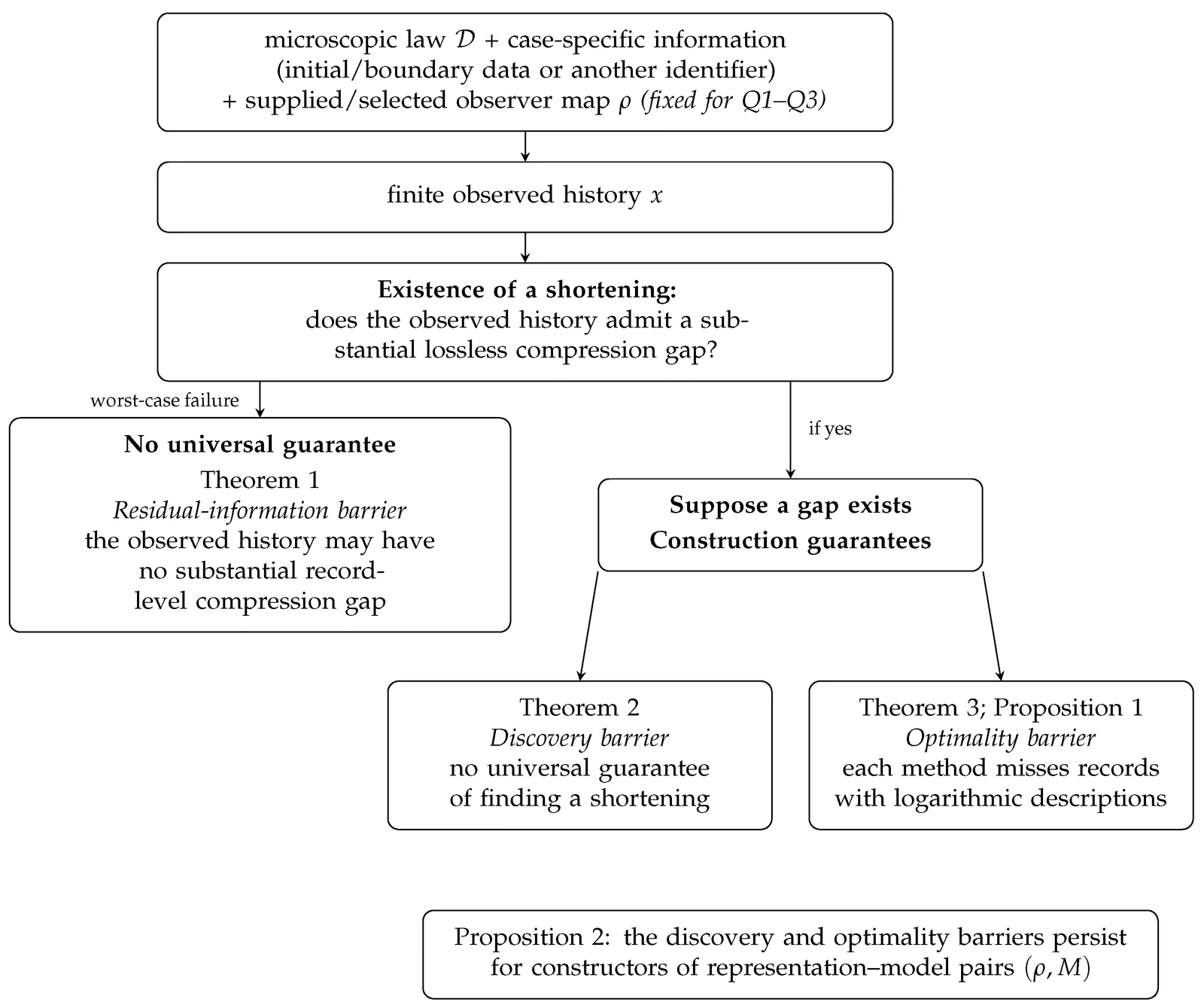
**Construction questions and guarantees.** Once the representation ρ is fixed, the microscopic law and case-specific information generate a finite observed history. The left branch asks whether that history has a substantial lossless compression gap; the right branch asks what a total computable constructor that always returns valid descriptions can guarantee when compression exists. The blind-spot bound applies at every sufficiently large record length for each fixed method. Proposition 2 transfers the discovery and optimality barriers to constructors that must return a representation together with a reusable model; the witness family is constructed in Appendix D.6; its guarantee concerns the available construction observations.

The existence claim concerns the lossless shortening of the specified history; it does not settle whether a model can capture some regularity or support a narrower predictive task. Figure 1 summarizes the three questions and their answers.

The barriers limit what any one method can guarantee, yet agents do discover reusable models. Such a discovery can carry the surprise often associated with emergence: the observer recognizes coherent organization that was not apparent in the microscopic description. A Game of Life glider crossing a cellular automaton and the coordinated motion of a flock are familiar examples  [16,17]; each invites a description in terms of collective entities and their behavior. Surprise is an explicit criterion in the emergence test of Ronald, Sipper, and Capcarrère [18].

For an isolated glider, a recurring shape and a translation rule describe many successive configurations through position, orientation, and phase; Section 5.3 works this case through. The model need not be optimal, and its description cost may be repaid only through repeated use. This connects the recognition of emergent organization to the compression progress associated with discovery  [1,6]. Scientific discovery and perceptual learning are instances; evolution can also build useful modeling capacity into organisms. Surprise motivates the definition without entering it. Acquisition changes the descriptions available to the observer, not the complexity of a fixed record under a fixed machine.

Definition 3 gives a sufficient certificate using explicit code lengths. A representation and model pass the test when, with their own description cost counted, they describe the data they keep in fewer bits than a baseline fixed in advance, and do so again on observations obtained later. Savings may arise across observations even when individual chunks cannot be shortened. Passing certifies reusable regularity. Failing does not rule it out, because a model can be useful before its description cost has been repaid.

The classical noncomputability results for Kolmogorov complexity are known. The contribution here is to locate them within the finite micro-to-macro problem and to carry them to the acquisition of a representation and reusable model. Problem 1 asks which effective restrictions allow one terminating method to find a qualifying pair whenever one exists, and Section 7 shows how Curie–Weiss symmetry, renormalization, hydrodynamic assumptions, and the Montbrió reduction constrain that search in physical systems. The record-level reductions are machine-checked in Lean 4; the model-acquisition proposition is proved at paper level (Appendix F).

Section 2 fixes the observer-relative setup and Section 3 proves the three barriers. Section 4 casts representation choice as a retained–residual split, and Section 5 gives the acquisition certificate and transfers the barriers to it. Section 6 shows how conserved information moves between the retained and the omitted coordinates, and what that costs a badly chosen representation. Section 7 examines the physical structures that make discovery feasible, and the Discussion separates these limits from answer-side undecidability. Appendix A collects the notation, and Appendix B develops the relation between compression and prediction.

## 2. Observer-Relative Setup

Fix a universal prefix-free Turing machine *U*, a finite observer-agent O, and a shared description frame $B_{0}$ containing its coding conventions and admissibility criteria. Write $C:=(O,B_{0})$ for the fixed observer/description frame and reserve ρ for a computable projection or coarse-graining. “Finite” means that the observer has finite physical state and computational resources at every finite time; no theorem depends on a particular architecture. Unless shown explicitly, complexities are relative to the fixed *U* and *C*, with their cost absorbed into additive constants. Tuples inside *K* use a fixed computable self-delimiting pairing in $B_{0}$. Conditioning on this frame is analogous to specifying the information available to Jaynes’s idealized reasoning robot [19,20].

We use *observer* and *agent* for the same system: agent when its actions are in view, observer when only its modeling is. It is the algorithmic agent of Kolmogorov Theory [21,22], which has a modeling engine that builds its world-model, an objective function, and a planning engine that selects actions; only the first two enter the results below.

The observer’s objective function OF specifies what it seeks to minimize. In a parametrized model, it may depend on the model and its changing parameters; more generally, it evaluates the program *M* in a declared context. The objective and its interpretation belong to the fixed setup. A relevance requirement translates that objective into distinctions the representation must preserve, at a declared accuracy, to evaluate or pursue it. A concrete task specifies the required inputs and outputs; equal objective values need not mean equal task behavior.

We model the observer’s construction procedures as Turing-computable, with resources finite at every finite time but no fixed memory bound across instances. Finiteness alone does not justify the computability restriction. Gandy’s analysis of discrete deterministic mechanisms shows that a device assembled from bounded-size parts within a bounded structural hierarchy, whose transitions depend only on causal neighborhoods of bounded size, computes only recursive functions [23]. Its physical assumptions are a lower bound on the size of a part and an upper bound on the speed of propagation of changes. An observer with a halting oracle, or an infinite-time machine [24], falls outside this class and can compute *K*. Theorems 2 and 3 place no restriction on it, while Theorem 1 applies unchanged because counting involves no computation. For ordinary oracle Turing machines, the computational barriers recur for description complexity defined relative to the same fixed oracle.

Let N={0,1,2,…}. Let ${\left\{0,1\right\}}^{*}$ denote the set of finite binary strings and ${\left\{0,1\right\}}^{+}$ its nonempty subset. For $x,y\in {\left\{0,1\right\}}^{*}$,

(1) K(x)=min{|p|:U(p)=x}, K(x∣y)=min{|p|:U(p,y)=x},

where *p* is a self-delimiting program, |p| and |x| denote bit lengths, and U(p,y) denotes execution with *y* supplied as auxiliary input. Equation (1) is the standard classical numerical notation; none of the operational claims requires a finite observer to possess or compute the exact value of *K*, and Appendix C gives a formulation directly in terms of description relations. For finite sets, |S| denotes cardinality. All logarithms are base two. The mutual algorithmic information of two strings is $I_{K}\left(a:b\right)=K\left(a\right)+K\left(b\right)-K\left(a,b\right)$, with K(a,b) the complexity of their fixed computable pairing. When common side information *z* is displayed, we use $I_{K}\left(a:b|z\right):=K\left(a|z\right)+K\left(b|z\right)-K\left(a,b|z\right)$. These symmetric forms agree with the usual directional definitions up to additive O(logK(a,b)) terms.

Unless stated otherwise, O(1) and O(logn) terms may depend on *U*, $B_{0}$, and fixed coding conventions, but not on the varying instance.

A procedure is *total computable* if it halts on every admissible input, and *uniform* when the same procedure must work at every input size. A predicate is *semidecidable* when positive instances are eventually found although negative searches may run forever; dovetailing interleaves candidate computations so every halting one is eventually seen. We call *x compressible* relative to its raw code when K(x)<|x|. When a procedure returns a standalone program *p* for a record *x*, its additive description regret is |p|−K(x)≥0. This is a static gap in bits. The benchmark |x| is the bit length of the data itself; the proofs below compare program length against it.

Let $X_{t}$ denote the substrate state at time *t*, and let $X_{\leq t}:={\left(X_{s}\right)}_{s\leq t}$ denote its finite history from a fixed starting time. The observer sees $P_{s}^{\rho }:=\rho \left(X_{s}\right)$ through a computable projection ρ acting at each time. Its observation history is $P_{\leq t}^{\rho }:={\left(P_{s}^{\rho }\right)}_{s\leq t}$. Write ${\mathrm{ME}}^{O}$ for the Modeling Engine implemented by observer O. It constructs the world model

(2) $$W_{t}={\mathrm{ME}}^{O}\left(P_{\leq t}^{\rho }\right).$$

**Figure 2:**
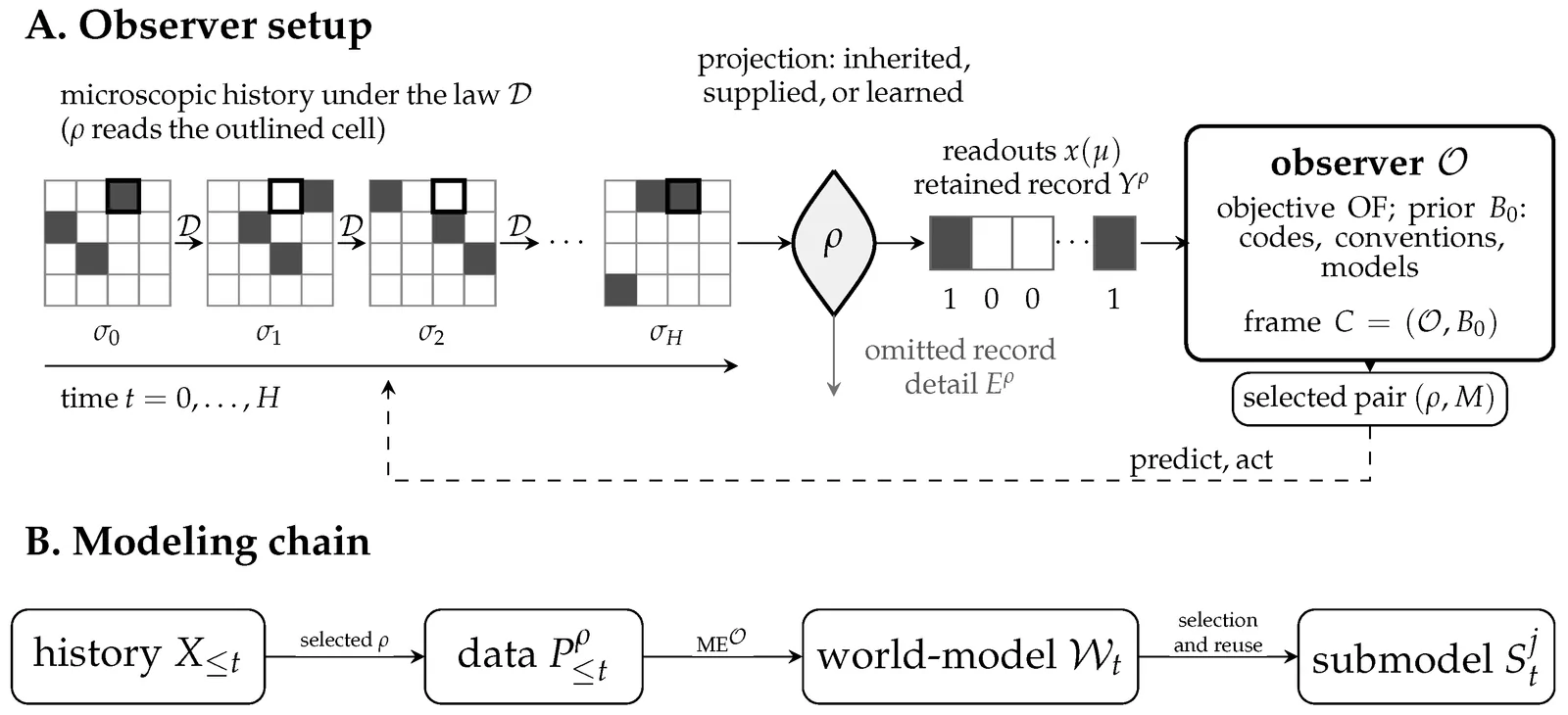
Observer-relative reconstruction. Panel (**A**) illustrates the observer setup: microscopic evolution generates successive readouts through a projection ρ (here ρ reads one outlined cell of a drifting pattern, so the record is one bit per step); relative to its prior frame $C=(O,B_{0})$, the observer models the retained record $Y^{\rho }$, omits residual detail $E^{\rho }$, and uses a selected representation–model pair (ρ,M) to predict or support action in pursuit of its objective OF. Panel (**B**): the observer need not receive the projection ρ as an intrinsic feature of the substrate: it may be inherited, supplied, or learned using the observer’s prior modeling resources. Conditional on the selected projection, O builds a world-model and retains candidate submodels that continue to compress, predict, or support action. The record-level barrier theorems concern this fixed-representation subproblem; the general emergence problem selects the representation and reusable model as one pair.

A candidate macroscopic submodel $S_{t}^{j}$, indexed by *j*, is a component of this structured world-model. Figure 2 shows these levels. We reserve calligraphic $W_{t}$ for the full world-model and use *M* for a candidate parametrized macromodel.

A projection is *admissible* when it belongs to a class specified under frame *C* and preserves the distinctions required by the objective. It cannot erase a target distinction that the declared task needs. Stored patterns and lookup tables are allowed, provided their acquired information is charged; unseen observations are not available as free side information. Admissibility is a modeling restriction, not a hypothesis needed by the record-level barrier theorems: those results require only that the displayed ρ be computable. In joint acquisition, ρ may be inherited, supplied, or learned together with *M*; Section 4.2 explains the relevance constraint. Here ρ specifies retained distinctions, not whether the observer acts on the system. A specified computable observer–world interaction can be included in the dynamics; any additional action information supplied to the decoder must enter the coding account.

*A note on “coarse-graining.”* We use the term in an observer-relative informational sense, broader than spatial averaging or the elimination of short-wavelength degrees of freedom. A coarse-graining is any computable representation that treats some distinctions in the substrate as irrelevant to the observer’s stated task while retaining others. A block average, an order parameter, a phase label, an object category, or a binary property such as “the device has halted” can all be coarse descriptions in this sense. What makes a variable macroscopic here is, therefore, not necessarily spatial scale: it is that the observer uses it as a higher-level description that ignores lower-level distinctions. Relevance is essential, since erasing all distinctions would produce maximal compression and no useful model.

To separate microscopic generation from macromodel construction, one must first specify the finite object that contains everything needed to simulate a single observed history.

**Definition 1** (Finite micro-experiment)**.**
*A* finite micro-experiment *comprises an update rule, an initial state, an observation map, and a finite horizon. We write it as $\mu =(D,\sigma_{0},\rho ,H)$. Let* Σ *be a finite microstate alphabet and N the number of microscopic degrees of freedom. The computable map $D:\Sigma^{N}\to \Sigma^{N}$ updates the initial state $\sigma_{0}\in \Sigma^{N}$, with $D^{0}\sigma_{0}=\sigma_{0}$. The computable map $\rho :\Sigma^{N}\to \Xi$ gives observations in a finite readout alphabet* Ξ*, and H∈N is the observation horizon, so the record contains H+1 observations. Its* macrohistory *is*

(3) $$x\left(\mu \right)=\left(\rho \left(\sigma_{0}\right),\rho \left(D\sigma_{0}\right),\ldots ,\rho \left(D^{H}\sigma_{0}\right)\right),$$

*encoded as a binary string using the fixed computable convention in $B_{0}$; for a fixed-width binary readout alphabet $\Xi ={\left\{0,1\right\}}^{r}$, this convention writes each r-bit block literally in temporal order (block boundaries are implicit in μ). In particular, for Ξ={0,1} the macrohistory is literally the readout bit sequence, and a single full-state readout from ${\left\{0,1\right\}}^{n}$ is encoded by its n bits.*

The notation distinguishes states from records. The finite experiment encodes the conceptual substrate state $X_{t}$ as $\sigma_{t}=D^{t}\sigma_{0}$, and its observed history $P_{\leq H}^{\rho }$ as the binary record x(μ). In the general modeling problem, *D* denotes a record before representation and $Y^{\rho }=\rho \left(D\right)$ the retained record; there ρ may act on the whole record. Section 6 uses $\Omega_{t}$ for a complete encoded state and $Q_{t}^{\rho }$ for its projected state. These are instantaneous states, rather than history encodings.

The tuple $\mu =(D,\sigma_{0},\rho ,H)$ is a convenient finite initial-value formal class for the barrier results, not a claim that fundamental physics must provide freely specifiable initial conditions. Section 3.5 rewrites the same information accounting in terms of boundary or global prescriptions and residual realization identifiers.

Complete knowledge of the experiment yields its macrohistory by direct simulation, giving the upper bound

(4) $$K\left(x(\mu )|C\right)\leq K\left(D,\sigma_{0},\rho ,H|C\right)+O\left(1\right),$$

and, with the common mechanism (D,ρ,H) supplied as side information,

(5) $$K\left(x(\mu )|D,\rho ,H,C\right)\leq K\left(\sigma_{0}|D,\rho ,H,C\right)+O\left(1\right).$$

These are the generation statements announced in the introduction: microscopic determination supplies a generative route and an upper bound on description length. The theorems of Section 3 ask what stronger conclusion follows.

A description of *x* is a standalone program *p* with U(p)=x. “Standalone” means that the auxiliary tape is empty: any side information must either be encoded in *p* or be made explicit through a conditional complexity K(x∣z). Two baselines are distinguished. The residual-information theorem conditions on the common law, observation map, and horizon and asks how much record-specific information remains. The discovery and optimality theorems give the procedure the complete experiment μ but require it to return a standalone program for x(μ). Appendix D shows separately that conditioning on the known laws does not restore computability.

Each experiment has a finite state space and horizon, and produces a finite record. The computational theorems concern a single procedure required to work as these bounds grow without a fixed maximum. On a fixed finite domain, answers could in principle be tabulated. The theorems concern lossless descriptions in an unrestricted program class; restricted predictive, causal, or intervention-based classes require their own analysis.

Simulation generates a record from the experiment. Compression seeks a shorter description of that record. Reusable model construction also selects a relevant representation and captures structure shared across records. The following sections develop these requirements in turn.

## 3. The Algorithmic Construction Barrier

### 3.1. Simple Microlaws Need Not Reduce Record-Specific Information

The first barrier does not rely on universal dynamics. It shows that simple microscopic access does not guarantee that the observer-visible history has a substantial compression gap relative to its raw code. Case-specific information can survive the observation process so completely that little of the raw length can be saved, even when the update rule and the instantaneous projection are both simple.

**Theorem 1** (Residual-information barrier)**.**
*For each integer n≥1, let $D_{n}$ be the cyclic left shift on the n-cell binary register and let $\rho_{n}$ read the leading cell, so that $K\left(D_{n},\rho_{n}|n,C\right)=O\left(1\right)$. Write $\mu_{n,y}$ for the micro-experiment with initial state $y\in {\left\{0,1\right\}}^{n}$ and horizon H=n−1; then $x(\mu_{n,y})=y$. For every integer d with 0≤d≤n,*

(6) $$\#\left\{y\in {\left\{0,1\right\}}^{n}\;:\;K\left(x\left(\mu_{n,y}\right)|D_{n},\rho_{n},n,C\right)<n-d\right\}\;<\;2^{n-d}.$$

*Hence at least a fraction $1-2^{-d}$ of the n-bit initial microstates produce macrohistories satisfying $K(x\left(\mu_{n,y}\right)|D_{n},\rho_{n},n,C)\geq n-d$.*

**Proof.**  Writing $y=y_{1}\cdots y_{n}$ and indexing readout steps by t=0,…,n−1, under $D_{n}$ the register content shifts one cell per step and $\rho_{n}$ outputs the cell now leading. The readout at step *t* is, therefore, $y_{t+1}$ and the *n*-step record is exactly *y*. Fix the conditioning string $z=(D_{n},\rho_{n},n,C)$. Conditional descriptions are programs for a fixed machine with *z* on the auxiliary tape, and there are fewer than $2^{m}$ programs of length less than *m*; taking m=n−d gives fewer than $2^{n-d}$ strings *y* with K(y∣z)<n−d. Since the map $y\mapsto x(\mu_{n,y})$ is the identity, (6) follows, and the complement has size at least $2^{n}-2^{n-d}$.    □

*Interpretation.* The shift register provides a minimal witness. For n>1, $\rho_{n}$ is pointwise many-to-one: each observation retains only one bit of an *n*-bit state. Yet the omitted bits are not lost from the *history*; the reversible shift feeds them into later observations until the complete initial state has been exposed. The example, therefore, separates pointwise lossiness from history-level lossiness. It establishes a non-guarantee, not a model of a typical natural system.

Under the uniform counting measure, at least a fraction $1-2^{-d}$ of records retain n−d bits of conditional complexity despite the known law, projection, and horizon; for d=10 this exceeds 99.9%. Relative to the *n*-bit raw record, the ideal compression gap is, therefore, at most *d* bits. The ensemble $S={\left\{0,1\right\}}^{n}$ is simple, but identifying the realized member still costs approximately *n* bits.

Theorem 1 concerns the lossless shortening of the realized history. Its counting measure does not assume that physical initial conditions are uniformly distributed. Restricting the initial or boundary information, or discarding information from the history, can reduce the description burden; compression requires that the retained history become shorter to describe than its raw code. Microlaws may still expose useful symmetries or predictive constraints. In particular, a simple stochastic law can describe an ensemble while identifying its realized member remains costly.

### 3.2. No Total Procedure Finds a Shortening Whenever One Exists

The second barrier is computational rather than informational. It asks only for improvement, not optimality: if the completed macrohistory has a lossless description shorter than its literal code, can one total procedure always find some such shortening?

No shift-register mechanism is needed. For each n≥1, let $I_{n}$ be the identity update on ${\left\{0,1\right\}}^{n}$ and let $\varrho_{n}:{\left\{0,1\right\}}^{n}\to {\left\{0,1\right\}}^{n}$ be the full-state readout. For an arbitrary nonempty string *y*, define the one-observation experiment

(7) $$\nu_{y}:=\left(I_{\left|y\right|},y,\varrho_{\left|y\right|},0\right).$$

Under the fixed-width readout convention of Definition 1, $x(\nu_{y})=y$. Thus $y\mapsto \nu_{y}$ is a computable embedding of arbitrary finite records into the formal experiment class. The computational barriers below require only this representational richness, not the information-leak mechanism used in Theorem 1.

A universal discovery rule would, therefore, have to exploit every compression gap in this embedded family while halting and returning a valid description on every input. The discovery barrier rules out that guarantee.

**Theorem 2** (Discovery barrier)**.**
*There is no total computable procedure A that, for every finite micro-experiment μ, returns a program A(μ) satisfying*

(8) U(A(μ))=x(μ),

*and is guaranteed to satisfy*

(9) K(x(μ))<|x(μ)| ⟹ |A(μ)|<|x(μ)|.

**Proof.**  Suppose such an *A* exists. Given any nonempty string *y*, construct $\nu_{y}$ by Equation (7) and compute $p=A(\nu_{y})$. By the unconditional validity condition (8), $U\left(p\right)=x\left(\nu_{y}\right)=y$, so |p|<|y| implies K(y)<|y|. Conversely, if K(y)<|y|, the guarantee (9) gives |p|<|y|. Comparing $\left|A(\nu_{y})\right|$ with |y| would, therefore, decide whether K(y)<|y|, contradicting undecidability of the compressible strings (Appendix C). The empty-string case can be hard-coded.    □

*Interpretation.* For every total procedure that always returns a valid lossless description, there exists at least one compressible record on which that procedure fails to shorten. A single such record defeats the guarantee. The theorem, therefore, does not say that no procedure ever compresses, that a given compressor cannot shorten most records of interest, or that all shorter programs must be produced; nor does it exclude open-ended search, which succeeds on positive instances without a stopping rule.

Even complete access to the finite micro-experiment does not provide a universal shortcut. “Complete” includes the microscopic rule, initial state, observation map, and finite horizon, so the record is straightforwardly computable by simulation. The reduction uses only that the formal experiment class can represent an arbitrary finite record through the trivial identity experiment. Classical undecidability of raw compressibility, therefore, transfers without invoking the shift-register witness or any assumption about incompressible initial states. Thus *a supplied program that reproduces the data gives an upper bound on its description length; finding a shorter description is a further computational task*.

*Finding a shortening, and knowing when to stop.* With the reference machine fixed, a finite record *x* presents three distinct tasks: exhibit a program shorter than *x*; establish that no such program exists; or determine the shortest program. For a threshold *b*, dovetail all programs shorter than *b* and return the first that halts with output *x*. If K(x)<b, this search eventually succeeds and the program is a finite witness. There is no computable bound on its waiting time valid for all positive instances, even when the bound may depend on *x* and *b* (Proposition A2). At any stipulated execution rate, some successful searches, therefore, outlast any fixed time budget, including the age of the universe. The same absence of a computable bound holds for the runtime of a fastest shortest program (Appendix C.2).

Certifying that a candidate is shortest instead requires excluding every shorter description. Continued simulation supplies no general stopping rule: short programs can halt extremely late, as measured by runtime Busy Beaver functions [25]. Shortness of the record alone gives no assurance of feasible certification. Particular instances and finite ranges can nevertheless be settled. A complete halting catalog through *b* program bits decides compressibility for every record of length at most b+1, and gives exact complexity for every output represented in it. Appendix C.2 gives the precise implication and a published example; the cutoff depends on the machine. An unresolved candidate need not obstruct a particular record if one can establish that it cannot output that record.

### 3.3. Optimality and Unbounded Additive Regret

The third barrier asks a different computational question. A procedure may find useful shortenings on many instances while still returning descriptions arbitrarily far from shortest. For a total computable procedure *A* on micro-experiments that always returns a valid program, define its additive description regret by

(10) $$R_{A}\left(\mu \right):=\left|A\left(\mu \right)\right|-K\left(x\left(\mu \right)\right)\geq 0.$$

Every fixed total constructor incurs arbitrarily large regret on some compressible records. The quantitative bound below will locate such blind spots at every sufficiently large length.

**Theorem 3** (Optimality barrier: unbounded regret on compressible records)**.**
*Let A be a total computable procedure that returns a valid description U(A(μ))=x(μ) for every finite micro-experiment μ. Then for every c∈N there is a finite micro-experiment μ whose macrohistory is compressible,*

(11) $$K\left(x(\mu )\right)<\left|x(\mu )\right|,\;\mathrm{yet}\;\left|A(\mu )\right|>K\left(x(\mu )\right)+c.$$

*Equivalently, the additive regret $R_{A}$ is unbounded for every such procedure already on the compressible instances.*

**Proof.**  Proposition 1 below gives witnesses with $K\left(x_{n}\right)\leq K\left(n\right)+c_{A}$ and $R_{A}\left(\nu_{x_{n}}\right)\geq n-K\left(n\right)-c_{A}$. Since K(n)=O(logn), choose *n* so that $n-K\left(n\right)-c_{A}>c$. Then $x_{n}$ is compressible and its regret exceeds *c*.    □

The following elementary counting-and-selection argument makes the failure quantitative.

**Proposition 1** (Blind spots at every length)**.**
*Under the hypotheses of Theorem 3, there is a constant $c_{A}$ such that, for every n≥1, some record $x_{n}\in {\left\{0,1\right\}}^{n}$ in the identity experiment family satisfies*

(12) $$|A\left(\nu_{x_{n}}\right)|\geq n,\;K\left(x_{n}\right)\leq K\left(n\right)+c_{A},\;R_{A}\left(\nu_{x_{n}}\right)\geq n-K\left(n\right)-c_{A}.$$

*Here K(n) uses the fixed integer encoding of the observer frame.*

**Proof.**  Validity makes $x\mapsto A(\nu_{x})$ injective: one program cannot output two distinct records. There are $2^{n}$ records of length *n* but only $2^{n}-1$ binary programs shorter than *n*. Let $x_{n}$ be the lexicographically first length-*n* record with $|A(\nu_{x_{n}})|\geq n$. It exists by counting and is computable from *n* by evaluating the total procedure on the finite ordered list. A fixed program implementing this search gives $K\left(x_{n}\right)\leq K\left(n\right)+c_{A}$. Subtracting yields the regret bound. The identity embedding preserves the record exactly.    □

*Exact answers with abstention.* Even a partial computable procedure that returns K(x) correctly whenever it answers can answer on only finitely many distinct records (Theorem A2, Appendix C.2; cf. ([26] Theorem 1)). This concerns guaranteed exactness on every answered instance, not a compressor’s incidental success in returning a shortest program without recognizing its optimality.

*Interpretation.* Every fixed total method has records it fails to shorten at each sufficiently large length, although those records have descriptions of only O(logn) bits. The bound implies Theorem 3 and Corollary 1, and supplies witnesses for Theorem 2 at every sufficiently large length. It describes worst-case blind spots, not typical performance. The constant $c_{A}$ depends on the method, reference machine, and fixed encodings; constructing $x_{n}$ may be extremely expensive. Thus the bound gives no numerical hardness guarantee for a particular short record. Appendix C.1 gives a witness-based version of unbounded regret that does not take exact numerical complexity as primitive.

Section 5 will translate between programs and the representation–model pairs defined there, and each translation costs a fixed number of bits. The hard instances must therefore stay hard after a fixed compression margin is required, so that they can pay that cost. The following strengthening lets the compression margin and the regret tolerance be set independently.

**Corollary 1** (Regret with a fixed compression margin)**.**
*Under the hypotheses of Theorem 3, for every c,l∈N there is a finite micro-experiment μ such that*

(13) $$K\left(x(\mu )\right)<\left|x(\mu )\right|-l,\;\mathrm{and}\;\left|A(\mu )\right|>K\left(x(\mu )\right)+c.$$

**Proof.**  Choose *n* with $n-K\left(n\right)-c_{A}>\max\left\{c,l\right\}$ in Proposition 1. Its witness satisfies both inequalities.    □

The discovery and optimality barriers are logically distinct. Discovery asks for a guarantee of *some* shortening whenever one exists. Bounded regret asks that every returned description stay within a fixed distance of *K*. A fixed-*c* approximation need not shorten records lying within *c* bits of their literal length, while a procedure that successfully shortens many records may still lie arbitrarily far above *K*. Both are computational obstructions, but they exclude different guarantees.

Appendix D records further technical variants: exact conditioning on the common microlaw does not make the residual complexity computable, and the open-ended below-threshold search has no computable universal schedule.

A separate limitation concerns proof rather than discovery: Chaitin incompleteness prevents a fixed sound formal theory from proving arbitrarily strong lower bounds on *K* (Appendix C.3).

### 3.4. Restricted Construction Problems

The computational reductions use the ability to represent arbitrary finite records, with microscopic simulation itself kept easy. Restricting the physical systems or observations may remove this embedding and permit a constructor on the restricted class. A positive result must identify both the source of the compression gain and a procedure that can find it. We first examine where the record-specific information can reside.

### 3.5. Source Accounting: Where Realization Information Can Reside

Theorem 1 raises an accounting question: where do the record-specific bits reside? For a computable deterministic evolution

(14) $$\sigma_{t+1}=D\left(\sigma_{t}\right),$$

with computable projection ρ, simulation gives

(15) $$K\left(x(\mu )|D,\rho ,H,C\right)\leq K\left(\sigma_{0}|D,\rho ,H,C\right)+O\left(1\right).$$

Theorem 1 shows that the right-hand side can scale with record length. The same inequality also identifies one route to making compression possible: if physics constrains the information selecting the realized trajectory, it constrains the residual burden. This is not yet a compression theorem, because the remaining burden must still fall substantially below the raw cost of the retained macrohistory.

*From an initial condition to a boundary rule.* Suppose first that an additional computable prescription B selects the initial state from the dynamics,

(16) $$\sigma_{0}=B\left(D\right).$$

Then

(17) $$K\left(x(\mu )|D,\rho ,H,C\right)\leq K\left(B|D,\rho ,H,C\right)+O\left(1\right),$$

and, if B is included in the known physical background,

(18) $$K\left(x(\mu )|D,B,\rho ,H,C\right)=O\left(1\right).$$

The information has not disappeared by notation: it has moved from the free initial datum $\sigma_{0}$ into the selecting prescription B. If B itself requires an arbitrarily long description, no algorithmic gain has been obtained. If instead B is a fixed, low-complexity physical principle, the particular shift-register witness of Theorem 1 is excluded on that restricted class. For sufficiently long records this can create a large compression gap relative to the physical background; for short finite records, fixed coding overheads can still consume the practical gain.

The formulation can be made independent of the language of initial conditions. Let Sol(D) denote the histories admitted by the dynamical law, and let a computable global prescription B restrict them to a family

(19) $$H_{D,B}\subseteq \mathrm{Sol}\left(D\right).$$

If this family contains more than one history, let ι denote whatever additional finite information is sufficient, together with (D,B), to select the realized history relevant to the finite record. Whenever this selection is computable, the information accounting has the schematic form  

(20) $$\begin{array}{cc} K\left(x|D,\rho ,H,C\right) & \leq K\left(B|D,\rho ,H,C\right)\\ & \;+K\left(\iota |D,B,\rho ,H,C\right)+O\left(1\right). \end{array}$$

Equation (20) generalizes Equation (15). Description cost can reside in the dynamical law, in a boundary or selection principle, or in the information needed to identify one realization among those that principle permits. A short description of a finite admissible family H does not make its typical members short: conditioned on H, fewer than $2^{m}$ members can have complexity below *m*. Thus a unique computably selected history makes the ι term O(1), whereas a simple large family can still require close to log|H| bits to identify the realized member. This is the ensemble–realization distinction of Theorem 1, with the information source made explicit. Bédard and Bergeron’s data-to-model index plays a related role: their boundary conditions locate a record within a model’s possibilities [15]. For the retained record, this corresponds to the realization coordinates and reconstruction corrections of Section 5; the omitted projection residual is separate.

*Hartle–Hawking and Penrose.* The Hartle–Hawking no-boundary proposal illustrates the global form above. Its law-like no-boundary condition defines a quantum state on regular histories  [27]; semiclassically, that state yields a weighted family of classical histories [28]. In the present bookkeeping, the condition plays the role of B, replacing an independently specified initial wave function by a global prescription. If that prescription uniquely and computably selected one history, the residual realization identifier would be trivial; if it specifies only a nontrivial family, the ι term in Equation (20) can remain substantial. The distinction does not depend on an interpretation of quantum probabilities.

Penrose’s Weyl-curvature hypothesis is a weaker example: it strongly restricts the early gravitational state without fixing every microscopic degree of freedom  [29]. In this language it narrows the admissible history family and may lower the information required of ι, but need not select a unique O(1)-complexity microstate. A boundary principle can, therefore, replace, constrain, or only partially determine what would otherwise be free realization data.

*A weaker escape: a complexity promise.* Between arbitrary realization information and an explicitly supplied compact selecting rule lies an intermediate case. Suppose the observer cannot reconstruct $\sigma_{0}$ but has a valid bound

(21) $$K\left(\sigma_{0}|D,\rho ,H,C\right)<s.$$

By Equation (15), the record then satisfies $K\left(x|D,\rho ,H,C\right)<s+c_{\mathrm{sim}}$ for a fixed simulator constant $c_{\mathrm{sim}}$. The arbitrarily large residual used in Theorem 1 is, therefore, excluded when *s* is fixed, but a useful compression gap exists only when this bound is substantially below the raw record length.

If the observer is supplied an explicit threshold *b* with K(x)<b, the open-ended search of Section 3.2 is guaranteed eventually to find a program shorter than *b*, but the promise supplies no computable universal deadline: otherwise running the search only that long would decide whether K(x)<b. Thus *guaranteed eventual discovery does not imply computably scheduled discovery*; the formal statement and proof are given in Appendix D. A promise of existence is still weaker than being given the program itself.

An explicit witness certifies an upper bound: if a program *p* with |p|<b is exhibited and its execution yields U(p)=x, then K(x)<b follows immediately. A method that always halts with a valid description cannot guarantee finding a shortening whenever one exists. Lower bounds and optimality require different evidence: Chaitin-type incompleteness (Appendix C.3) limits sufficiently strong lower-bound proofs in a fixed sound formal theory, without limiting explicit upper-bound witnesses.

There is another useful distinction between a set being finite and a search through it being known to finish. The promise $K(\sigma_{0})<s$ implies that only finitely many programs shorter than *s* could describe $\sigma_{0}$, but there is no computable stage at which one knows that all halting programs of that length have appeared. By contrast, if physics supplies an explicit parametrization

(22) $$\sigma_{0}=g\left(\theta \right),\;\theta \in \left\{1,\ldots ,J\right\},$$

with *g* total and the finite number of candidates *J* explicitly known, all candidates can be generated, simulated, and checked under a computable schedule. Physics has then replaced unrestricted program search by a decidable finite model class.

*Stronger prescriptions buy stronger guarantees.* A concise boundary prescription that selects the realization can make long records simple relative to the physical background; a promise K(x)<b guarantees eventual open-ended discovery but no computable deadline; an explicit witness settles its instance and an explicit finite model class $\sigma_{0}=g\left(\theta \right)$ makes exhaustive search computably schedulable *within that class*. None guarantees that a supplied class contains every globally available compressor.

These restrictions may also break the arbitrary-record reductions behind the discovery and optimality barriers, so the present impossibility proof need not transfer. That is a boundary on the negative result, not a positive constructor theorem. Source accounting is the common ledger: realization information must reside in the initial state, boundary rule, realization identifier, or law; be rendered irrelevant by discarding information from the history; or come from a compact source. Moving arbitrary bits between accounts does not reduce their description cost, and reducing the burden creates compression only when the retained history is simpler than its raw code.

## 4. Structure Functions and the Geometry of Discovery

Source accounting asked where the case-specific information resides. The next question is how a description should divide its cost between a model and the information that identifies one record among those the model covers. Algorithmic statistics quantifies this division: its structure function balances the cost of specifying a macrostate against the information needed to locate the realized microstate within it.

### 4.1. Macrostates, Model Budget, and Cracks

For a finite record $x\in {\left\{0,1\right\}}^{n}$, read a finite set S∋x as a candidate *macrostate* (literally, a set of states): it declares which microrecords remain equivalent at that descriptive level. Its cost is K(S), while log|S| bits suffice to identify the realized *x* once the macrostate is known. For a model-description budget α, Kolmogorov’s structure function is

(23) $$h_{x}\left(\alpha \right)=\min_{S}\left\{\log|S|:x\in S,\;K(S)\leq \alpha \right\}.$$

Thus $h_{x}\left(\alpha \right)$ is the residual index length of the tightest macrostate containing *x* that is specifiable within model budget α. The trivial macrostate $S={\left\{0,1\right\}}^{n}$ costs only the description of *n* but leaves *n* residual bits.

Once a model S is available, extra model bits can specify progressively finer cells of S: listing S in canonical order and fixing *k* leading index bits selects a cell of size $\left|S\right|/2^{k}$ that is describable from S, *k*, and the cell index. Hence, as long as residual information remains, ordinary refinement gives, up to the usual logarithmic precision,

(24) $$h_{x}\left(\alpha_{2}\right)\leq \max\left\{0,h_{x}\left(\alpha_{1}\right)-\left(\alpha_{2}-\alpha_{1}\right)\right\}+O\left(\log n\right),\;\alpha_{2}>\alpha_{1}.$$

**Figure 3:**
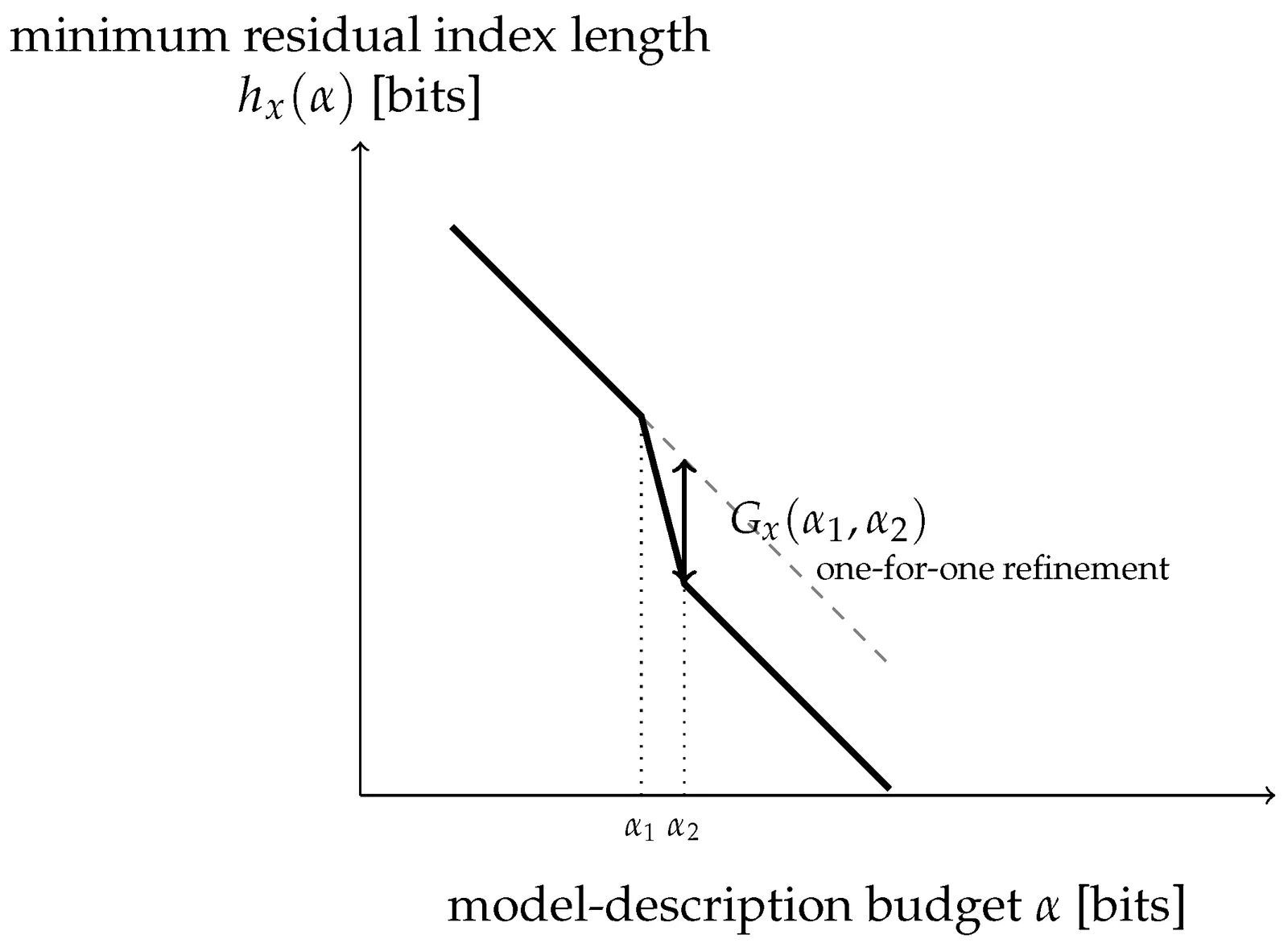
Schematic structure-function frontier. Ordinary refinement trades roughly one additional model bit for one fewer residual bit (dashed slope-−1 reference). A crack is a finite interval in which the residual falls appreciably faster; equivalently, the total two-part cost $\alpha +h_{x}\left(\alpha \right)$ improves. The horizontal axis is description budget, not discovery time; a crack marks a candidate descriptive gain and does not establish acquisition, relevance, or reuse. Discreteness and lower-order coding terms are suppressed.

A one-bit increase in model description can, therefore, buy roughly one bit less residual without revealing any new economy: information has merely moved from the index into the macrostate description. Candidate discoveries are drops substantially steeper than this one-for-one baseline (Figure 3).

For an interval $\alpha_{1}<\alpha_{2}$, define the net structure gain

(25) $$G_{x}\left(\alpha_{1},\alpha_{2}\right):=h_{x}\left(\alpha_{1}\right)-h_{x}\left(\alpha_{2}\right)-\left(\alpha_{2}-\alpha_{1}\right).$$

We call an interval a *crack* when $G_{x}$ is positive by an amount resolvable above the O(logn) coding uncertainty: a small increase in model complexity removes more residual information than it costs. Since α is discrete and Kolmogorov complexities are defined only to lower-order precision, “slope” and “discontinuity” are schematic descriptions of this finite-difference event.

Bédard and Bergeron [15] develop a more refined version of this idea. Their modified structure-function construction is designed so that ordinary slope-−1 segments correspond to refinement within the same minimal partial model, whereas nontrivial drops mark transitions to successively richer partial models. Our $G_{x}$ uses the ordinary structure function and asks only whether an interval establishes net description gain beyond one-for-one refinement. It is, therefore, a weaker geometric diagnostic, used here to nominate *candidate algorithmic discovery events*; Section 5 asks whether such an economy can be reorganized as a reusable parametrized compression that continues to work on new data.

The corresponding constrained two-part, or MDL, function is

(26) $$\lambda_{x}\left(\alpha \right)=\min_{S}\left\{K(S)+\log|S|:x\in S,\;K(S)\leq \alpha \right\}.$$

It does not define a second frontier. With the definitions above,

(27) $$\lambda_{x}\left(\alpha \right)=\min_{0\leq \beta \leq \alpha }\left\{\beta +h_{x}\left(\beta \right)\right\}.$$

Thus $\lambda_{x}$ is the running minimum of the same model–residual frontier after the one-for-one trade is charged explicitly. Along an ordinary refinement, $\beta +h_{x}\left(\beta \right)$ is approximately constant; a crack lowers it when the new model establishes a better total description. The MDL view is, therefore, often more intuitive economically, but it contains no separate discovery geometry.

When a macrostate satisfies

(28) K(S)+log|S|=K(x)+O(logn),

it is an algorithmic sufficient statistic: at that precision, all compressible regularity has been assigned to the macrostate and the remaining index is irreducible  [30,31].

This picture also reconnects to the discovery barrier. For a residual target *r*, the statement $h_{x}\left(\alpha \right)<r$ asserts the existence of a finite-set model within budget α whose residual is below *r*. Dovetailed search finds such a model when it exists but need not halt when it does not; indeed, the unrestricted structure function is not computable  [31]. The frontier is an ideal description of what can be discovered, not a computable map handed to the observer.

*From the structure-function split to a coarse-graining.* The structure function is an abstract retained–residual frontier. A projection ρ gives a concrete realization of the same split. Here *D* denotes the finite pre-representation record at the modeling grain under discussion, and ρ the corresponding computable record-level representation map. For a time series this may be induced by, or extend, a pointwise observation map; in the barrier theorems that observation map is already fixed and x(μ) denotes its output. Fix a finite computable record domain X as part of the declared frame; let D∈X, and define

(29) $$Y^{\rho }:=\rho \left(D\right),\;S_{\rho ,Y}:=\left\{D^{\prime }\in X:\rho \left(D^{\prime }\right)=Y^{\rho }\right\}.$$

The set $S_{\rho ,Y}$ is the fiber of ρ: the records that share the retained value $Y^{\rho }$.

Choose a self-delimiting residual index $E^{\rho }$ identifying *D* inside this finite fiber. Then, at the declared finite domain/resolution and up to fixed coding overhead,

(30) $$D\;⟷\;(Y^{\rho },E^{\rho }).$$

Moreover,  

(31) $$\begin{array}{cc} K(S_{\rho ,Y}|C) & \leq K\left(\rho ,Y^{\rho }|C\right)+O\left(1\right),\\ \left|E^{\rho }\right| & =\lceil \log|S_{\rho ,Y}|\rceil +O\left(1\right). \end{array}$$

for the canonical index code within the finite fiber; the fixed domain X is part of the conditioned frame *C*. Thus $\left(\rho ,Y^{\rho }\right)$ realizes a concrete point on, or above, the ideal structure-function frontier.

We call $Y^{\rho }$ the *retained algorithmic kernel* relative to ρ: it is the part of the record kept by the observer’s representation. The coordinate $E^{\rho }$ is the realization detail discarded by the lossy coarse-graining. Thus a candidate ρ selects one particular model–residual decomposition from the broader structure-function frontier. Operationally, science keeps the kernel and throws away the residual; the lossless pair in Equation (30) is only the AIT bookkeeping device that tells us exactly what was discarded. The kernel is what the representation keeps; it may itself remain incompressible, and modeling it is the next step.

This also refines the usual MDL decomposition. Once a reusable model *M* of $Y^{\rho }$ is introduced, the data term L(·), an actual code length, separates up to coding overhead as

(32) $$L\left(D|M,\rho \right)\approx L\left(\theta |M,\rho \right)+L\left(E^{\rho }|M,\rho ,\theta \right),$$

where θ denotes meaningful coordinates of the retained kernel. The first term is not “noise”: it is retained model state or realization-specific parameter information. The second is the omitted residual. Section 5 makes this reusable interpretation explicit.

### 4.2. Relevance-Constrained Coarse-Graining

The main barriers fix the projection ρ. Joint construction must also choose what to retain and what to omit. The objective supplies the reason for a relevance constraint: without one, a constant map could discard everything, while the identity map could retain everything. Neither extreme alone establishes useful compression.

For fixed (D,N,H,Σ), define

(33) $$H_{\rho }:=\left\{x\left(D,\sigma_{0},\rho ,H\right):\sigma_{0}\in \Sigma^{N}\right\},$$

the macrohistory family induced by ρ. These dynamically realizable families form a restricted subclass of the arbitrary finite-set macrostates in Equation (23), with only the general upper bound

(34) $$K\left(H_{\rho }|C\right)\leq K\left(D|C\right)+K\left(\rho |C\right)+K\left(N,H,\Sigma ,\Xi |C\right)+O\left(1\right).$$

This bound controls the cost of naming the history family induced by a candidate ρ; it does not compare candidates, because changing ρ changes both the family and the retained data.

Fix the objective OF and a nonempty, effectively encoded class $R_{C}$ of admissible projections under frame *C*. The relevance criterion states which distinctions are needed to evaluate or pursue that objective, and how their preservation is assessed. It constrains acceptable omission; the compression certificate separately charges the representation and model of what remains.

Predictive sufficiency, dynamical closure, and information about a designated target provide different relevance criteria. Predictive sufficiency identifies histories that induce the same predictive distribution for a designated future target, as in computational mechanics [32]. Exact dynamical closure requires a macro-update $\bar{D}$ satisfying $\rho \circ D=\bar{D}\circ \rho$. Intervention compatibility additionally requires the representation and model to reproduce the effects of the specified interventions. These conditions support different scientific claims: prediction of a target, autonomous macrodynamics, and causal or interventional adequacy. Compression alone establishes none of them. For a finite target record *z*, retained algorithmic information $I_{K}\left(Y^{\rho }:z|C\right)$ is an ideal diagnostic; its evaluation is uncomputable, in general, so an effective test needs a computable surrogate or restricted structure. Israeli and Goldenfeld give a constructive closure example by searching a finite class of local block maps [33]. Finiteness belongs to that example; the present formulation allows infinite classes of programs.

The constructive target is the pair (ρ,M): ρ preserves the relevant distinctions and *M* supplies a reusable code for what it retains. The two programs may be discovered together or refined in alternation. Supplying ρ fixes one component of this same construction task. Section 5 gives the certificate, proves the general construction barrier, and asks which effective restrictions restore a guarantee (Problem 1).

## 5. Algorithmic Emergence: Acquiring Reusable Regularity

### 5.1. Models Shared Across Observations

Figure 2 shows the setup: the observer receives a history through ρ and acquires a pair (ρ,M) to predict or support action. We now ask what that acquisition achieves. Let observations arrive in chunks $D_{m}=\left(D_{1},\ldots ,D_{m}\right)$, with supplied conditions $u_{m}=\left(u_{1},\ldots ,u_{m}\right)$. Here $D_{i}$ denotes a data chunk, whereas D denotes the microscopic update rule. The retained sequence is $Y_{m}^{\rho }=\left(\rho \left(D_{1}\right),\ldots ,\rho \left(D_{m}\right)\right)$. The representation ρ may act on a whole chunk; applying the same state readout at each time is a special case. The objective OF determines which distinctions must survive this representation; the model describes regularities within and across the retained observations. Omitted detail belongs to $E^{\rho }$, as in Section 4.

A sequence of hand images makes the distinction concrete  [11]. The shared model describes a hand; changing parameters describe its pose, rotation, and shape. If the objective concerns hand movement, background detail may be omitted. A recurring gesture also makes the parameter sequence compressible. Individual images need not become shorter: the saving can come from what the sequence shares. Repeated copies of an otherwise incompressible image are another example. Storing that image once and referring back to it captures a real regularity.

Let the finite model program $M=(e_{M},g_{M})$ provide computable encoder and decoder maps, total on the declared domain of finite observation blocks. Write

(35) $$e_{M}\left(Y_{m}^{\rho },u_{m}\right)=\theta_{m},\;g_{M}\left(\theta_{m},u_{m}\right)=Y_{m}^{\rho }.$$

Here $\theta_{m}$ denotes the realization coordinates or states for the block, represented by one joint code. Its length $L_{M}\left(\theta_{m}|u_{m},C\right)$ includes all framing and any corrections needed for exact reconstruction of the retained data. Approximate predictions can, therefore, support a lossless description of the retained observations when their correction data are included and charged. Separate parameter codes with total length $\sum_{i}\left|\theta_{i}\right|$ are a special case; joint or sequential coding can exploit relations among them. The same interface assigns coordinates a stable role across observations, such as pose, object identity, initial state, phase, or an intervention handle. A learned neural encoder may supply those coordinates without being human-readable. Supplied conditions and deterministically recovered state can be used by both encoder and decoder; additional information must be encoded. When the coordinates and conditions are available before a future observation, the same decoder also supplies a prediction.

The complete retained description has length

(36) $$L_{\mathrm{keep}}\left(D_{m};\rho ,M|u_{m},C\right)={\left|\langle \rho ,M\rangle \right|}_{C}+L_{M}\left(\theta_{m}|u_{m},C\right).$$

The first term is the actual additional self-delimiting code needed to acquire the pair beyond the prior frame *C*; it is paid once. It is not the time or energy spent learning. Either component may reuse prior resources, and an inherited or supplied ρ has zero incremental cost. The second term describes the observations through that shared model. Appendix B gives the full accounting when omitted detail is restored.

Even a large, inelegant implementation can capture regularity and repay its cost through reuse. This raises a separate question: how much of its program length is needed to reproduce the behavior that matters? Different programs and algorithms can compute the same function  [34,35]. We compare implementations by a declared task T that specifies the relevant input/output behavior. The objective motivates this task, but equality of objective values alone would not establish behavioral equivalence. A shortest equivalent program is the model’s *functional core*; for a coding task it is the minimum reusable program cost of that compression strategy.

**Definition 2** (Task-relative functional core)**.**
*Fix a finitely encoded task T with admissible input domain $X_{T}$ and a computable interface specifying the observed output. Let $F_{M}^{T}$ denote the behavior induced by model M, computable on that domain. Write*

(37) $$M\equiv_{T}N\;⟺\;F_{M}^{T}\left(x\right)=F_{N}^{T}\left(x\right)\;\mathrm{for}\;\mathrm{every}\;x\in X_{T}.$$

*The* functional core complexity *of M relative to the pre-acquisition frame C is*

(38) $$\kappa_{T}\left(M|C\right):=\min\left\{|p|:U\left(p,\langle x,T,C\rangle \right)=F_{M}^{T}\left(x\right)\;\mathrm{for}\;\mathrm{every}\;x\in X_{T}\right\}.$$

*A* functional core *is any program attaining this minimum. If the declared behavior yields systematic coding gains, such a representative is a* compressive core *of M.*

For the generative interface in Equation (35), the behavior may be $\left(\theta ,u\right)\mapsto g_{M}\left(\theta ,u\right)$; for a predictor, it may be a next-observation map or a conditional predictive distribution. A core preserves that behavior, including its errors and memorized exceptions. It is the shortest among equivalent implementations; another model may predict or compress better. The core need not be a localized subprogram of the acquired implementation.

Consider a program that predicts planetary trajectories using a Newtonian solver. A second program runs the same solver but also executes a finite Minecraft simulation before returning each prediction. The game’s state and output never affect the celestial-mechanics calculation, and its output is discarded. Both programs, therefore, return exactly the same predictions, although the second can contain much more code and take much longer to run. For this prediction task, they have the same functional core complexity: the additional game contributes nothing to the declared predictive behavior.

Additional code can also serve a purpose. A third implementation could store exact precomputed answers to frequent queries, returning those answers directly and using the original Newtonian solver for all other inputs. At the declared precision, its predictions remain identical, but the stored answers can reduce response time [36]. Its functional core complexity is again unchanged. These examples distinguish the minimum code needed to reproduce the predictions from the cost and speed of a particular implementation. A response deadline imposes an additional runtime requirement.

Finding a shortest equivalent program is not computable in general: for a fixed task with a single admissible input $x_{0}$ and declared behavior $F_{M}^{T}\left(x_{0}\right)=w$, the functional core complexity is K(w∣z), where $z=\langle x_{0},T,C\rangle$ is fixed. No total computable procedure computes this value for all *w* (Appendix C.2). Even guaranteed shortening is impossible in general: no total computable procedure can always return an equivalent program and shorten it whenever a shortening exists. Repeated application of such a procedure, stopping when the length no longer decreases, would yield a shortest equivalent program.

The functional core is, therefore, an ideal benchmark; the observer must pay for the implementation it actually acquires. Appendix B gives the condition under which repeated coding gains repay that cost.

### 5.2. Algorithmic Emergence: Acquisition and Reuse

Hold fixed the observer’s pre-acquisition frame $C=(O,B_{0})$, objective OF, and relevance requirements. They determine the admissible projection class $R_{C}$. Changing the model or its coordinates does not change the objective. Relevance constrains acceptable omission; it does not guarantee that the retained data contain a compressible regularity.

Let $B_{\rho }\left(Y_{m}^{\rho }|u_{m},C\right)$ be a computable baseline code length for the retained sequence, with decoding rules and supplied information fixed before candidate selection. It may be a literal code or an incumbent compressor. A sum $\sum_{i}B_{\rho }\left(Y_{i}^{\rho }|u_{i},C\right)$ is one allowed baseline. Use fixed encodings of the required variables so that padding cannot inflate the apparent savings, and require $B_{\rho }\left(Y_{m}^{\rho }|u_{m},C\right)\leq L_{0}\left(D_{m}|u_{m},C\right)$, where $L_{0}$ is the declared original-data baseline. These comparisons concern valid codes, not arbitrary numerical scores.

**Figure 4:**
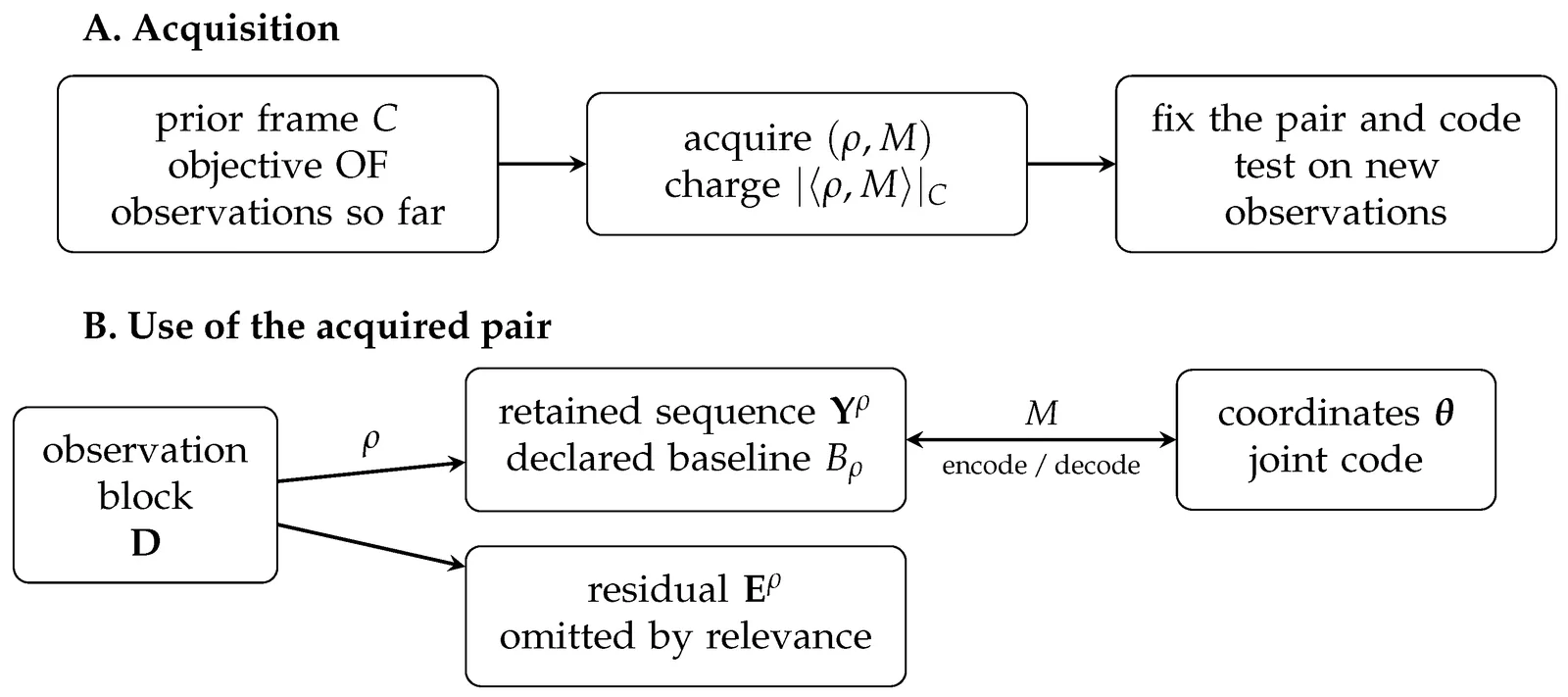
Acquisition and use of a reusable model-compressor, within the observer setup of Figure 2. The prior frame *C*, objective OF, relevance requirements, and baselines precede selection. The pair’s incremental code cost is charged once; an inherited or supplied ρ has zero incremental cost. The same model jointly encodes the retained observations through changing coordinates, while detail not required by the objective is omitted. New observation blocks test further savings with the pair fixed (Definition 3).

Choose fixed positive total margins $\delta_{\mathrm{comp}}$ and $\delta_{\mathrm{test}}$ before selection. After acquisition, evaluate the pair on a new finite observation block. Write $h_{m}$ for any history or model state already available to both encoder and decoder from the construction observations. Both test codes may use this same declared information; any extra state must be paid for. Figure 4 summarizes this acquisition and reuse accounting.

**Definition 3** (Compression-certified algorithmic emergence)**.**
*Relative to the pre-acquisition frame C,* compression-certified algorithmic emergence *occurs when O acquires a pair (ρ,M) exposing previously unexploited regularity and meeting the following requirements.*

(i) Relevance to the objective. *$\rho \in R_{C}$ retains the distinctions required to evaluate or pursue OF, under the declared accuracy and domain. The omitted detail $E^{\rho }$ is irrelevant, or acceptably irrelevant, under these requirements.*
(ii) Reusable model and coordinates. *The same finite model $M=(e_{M},g_{M})$ and coordinate interface satisfy Equation *(35) *throughout the declared domain of finite observation blocks. The representation, model, and coding rules remain fixed during evaluation; their declared state and parameter values may change as observations arrive. The coordinates have declared roles that remain fixed across observations—for example, pose, object identity, state, or phase—while their values may change. Models without realization parameters are also allowed; any framing or correction data needed for reconstruction still count toward the code length.*
(iii) Net compression of the retained observations. *With the observations collected so far, the complete retained code beats its baseline:*
  (39) $$L_{\mathrm{keep}}\left(D_{m};\rho ,M|u_{m},C\right)\leq B_{\rho }\left(Y_{m}^{\rho }|u_{m},C\right)-\delta_{\mathrm{comp}}.$$
(iv) Prospective reuse. *On a new observation block acquired after the pair is fixed, its coordinate code satisfies*
  (40) $$L_{M}\left(\theta_{\mathrm{test}}|u_{\mathrm{test}},h_{m},C\right)\leq B_{\rho }\left(Y_{\mathrm{test}}^{\rho }|u_{\mathrm{test}},h_{m},C\right)-\delta_{\mathrm{test}}.$$
  *The acquisition cost paid in (iii) is not charged again. Decoding uses only the acquired pair and the declared shared information, and reconstructs the retained test block exactly.*

The inequalities certify compression and reuse relative to the declared baseline. They do not by themselves establish historical novelty: a model already usable in the prior frame does not become a new acquisition merely by beating that baseline.

The certificate measures compression *within* the retained observations; omitting detail alone does not satisfy (iii). Savings can come from a shared object model, regular changes in parameters, or simple repetition. A stored pattern qualifies when its use provides new net savings relative to the declared baseline, with its storage cost counted. Neither margin requires every chunk to be shortened. A useful regularity can be acquired before enough observations have arrived to repay its implementation cost; the certificate then remains unmet. Passing the prospective test shows reuse on the tested observations, without guaranteeing every future continuation.

The ideal quantities involving *K*, the explicit certificate, and empirical estimates serve different purposes. The first describe shortest codes but are generally uncomputable. For a candidate with an established admissible coding interface, the certificate checks finite reconstruction and actual code lengths; it does not decide totality or relevance for arbitrary programs.

At the empirical level, the Coding Theorem Method estimates complexity from small-program output frequencies, and the Block Decomposition Method combines local estimates and block multiplicities [37,38]. Such scores can guide search, but passing the certificate requires a complete lossless code, including its model, arrangement information, and framing. A periodic record can have balanced symbol frequencies while admitting a short description of its repetition, so single-symbol frequencies alone can miss regularity. Perturbation-based methods provide another route to proposing generative models [39]. Any computable method that always terminates with a valid description remains subject to Proposition 1. Numerical scores alone are not descriptions.

Prospective reuse concerns coding observations obtained after selection. Advance prediction additionally requires the relevant coordinates or predictive distribution before those observations arrive.

### 5.3. Reusable Objects and Coarse Laws

Two examples help to make Definition 3 concrete. In both, the observer already knows the microscopic law and can simulate; the question is what a further model adds and whether its reuse repays its cost. The first model captures a recurring object, the second a law for a coarse variable. The register example in Theorem 1 illustrates a different obstruction: the record can retain so much initial-condition information that no substantial shortening exists.

**Figure 5:**
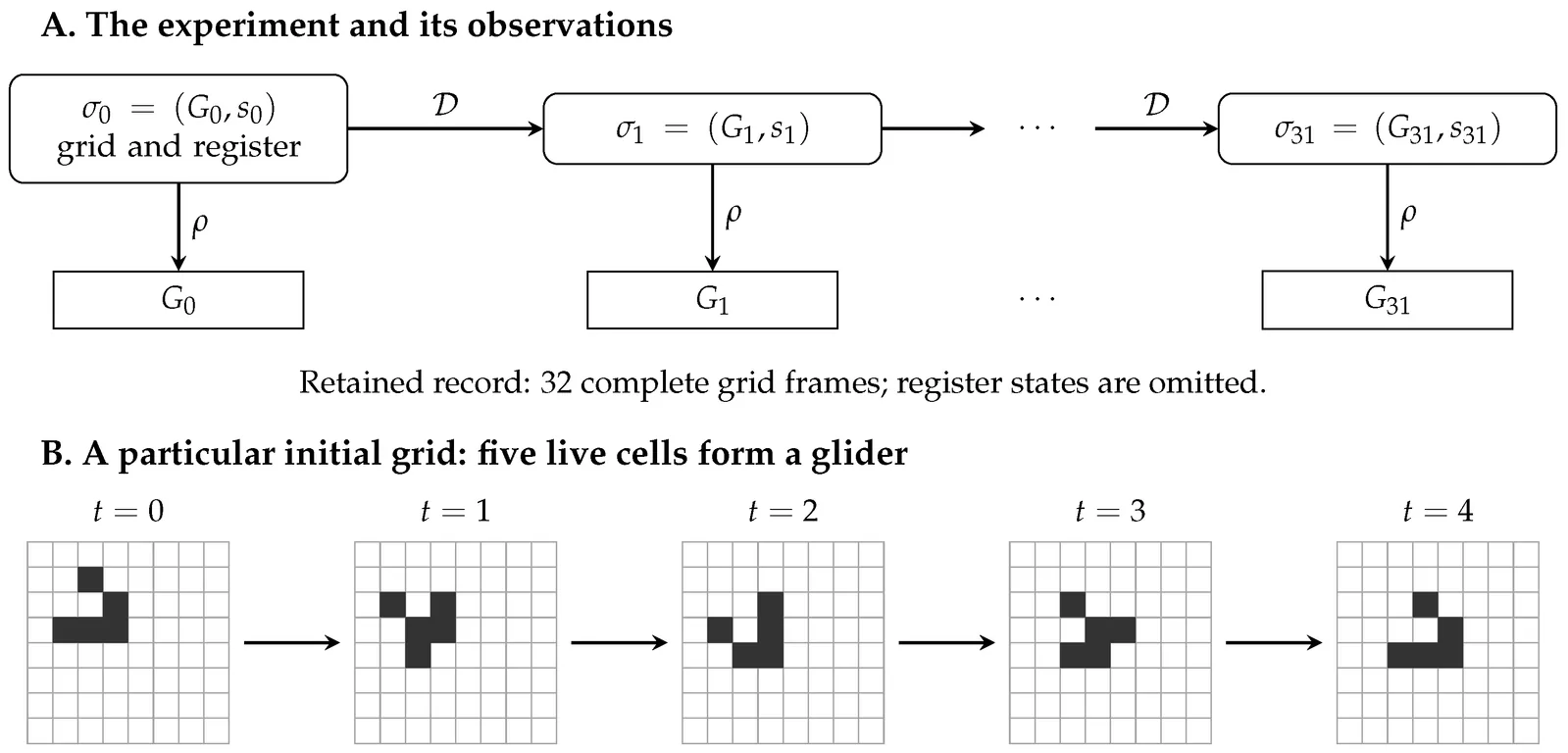
The finite micro-experiment used in the worked compression example. (**A**) Each microstate contains the entire binary grid $G_{t}$ and register $s_{t}$. The update D applies Life to the grid and a cyclic shift to the register. The observation map ρ retains the grid at each of 32 times; both competing codes must reconstruct this same grid movie. (**B**) Local windows show the first five grid states for the chosen initial configuration. Shaded cells are alive and arrows indicate one Life update. The shape at t=4 is the shape at t=0 translated one cell down and right. The intermediate shapes specify the four phases used by the glider model. Position, phase, and direction reconstruct the past frames and predict later ones under the same translation rule. The full grid has periodic boundaries.

*A recurring object.* The observer’s objective is to predict live-cell positions and retain 32-frame movies of Conway’s Game of Life, a two-dimensional binary cellular automaton [16]. On our 256×256 periodic grid, all cells update simultaneously: a live cell survives with two or three live neighbors among its eight neighbors, and a dead cell becomes live with exactly three. Each microstate contains the grid *G* and an independent 4096-bit cyclic register *s*; the update D applies Life to *G* and a one-cell cyclic shift to *s*. The observation map ρ(G,s)=G retains the grid, which is the variable required by the objective. Figure 5 defines the experiment and shows an initial five-cell configuration called a *glider*: its shape recurs after four Life updates, translated by one cell in each coordinate.

A model storing that cycle and translation reconstructs the past frames and predicts later ones from the same initial position, phase within the four-step cycle, and direction. Those coordinates change across runs while the model is reused. Against an incumbent that already stores the five live-cell positions and simulates Life, the model saves 65 bits per restarted run before its acquisition cost is charged. The supplied implementation passes the 128-bit construction margin after 294 runs and the same reuse margin on two subsequent runs; a single movie does not repay it. Appendix B.2 gives the complete accounting and coding conventions.

*A law for coarse observations.* The observer now seeks to predict which blocks of neighboring cells are fully occupied. The microscopic system is an *elementary cellular automaton* (ECA): a line of binary cells updated simultaneously, each from its own bit and its two nearest neighbors. Rule numbers follow Wolfram’s convention [40]. We use Israeli and Goldenfeld’s  [33] example of rule 146, which outputs 1 for neighborhoods 001, 100, and 111, and 0 otherwise. Our periodic ring has 360 cells, grouped into 120 aligned triples and observed every three microscopic steps.

**Figure 6:**
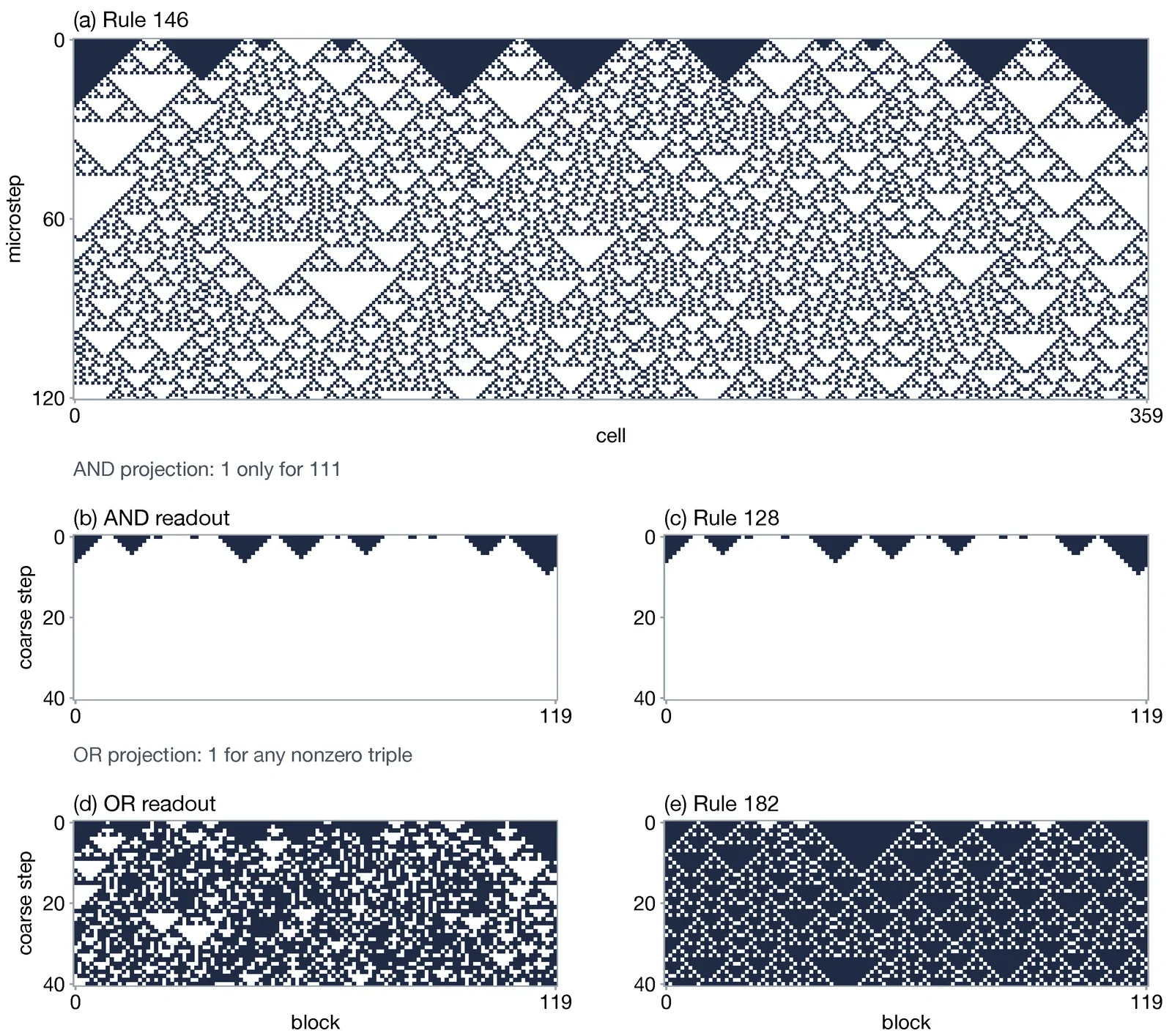
A closed coarse law and a failed elementary-rule search for the same microscopic run. (**a**) Rule 146 evolves a 360-cell periodic ring from the displayed initial row. (**b**,**d**) Each aligned triple is mapped to one bit every three microscopic steps: AND is 1 only for 111; OR is 1 for any nonzero triple. (**c**,**e**) The selected coarse rule runs from the corresponding first coarse row. Rule 128 reproduces the AND record exactly. Rule 182, which minimizes one-step error among the 256 elementary rules for the OR record, diverges from it. The observed transitions have zero closure conflicts for AND and 1476 for OR, counted by the convention in Appendix B.2. Shaded cells denote 1; time increases downward. The same projection is used on both sides of each reconstruction comparison.

The readout is 1 only for the triple 111, the Boolean AND projection. Israeli and Goldenfeld showed that its future follows rule 128 exactly: a coarse cell remains occupied precisely when it and both neighboring coarse cells are occupied, so occupied intervals shrink from their ends. The initial coarse row and this law generate every later retained row, independently of the discarded microscopic detail (Figure 6b,c). In the declared code, acquisition and the first movie cost 151 bits against the microscopic simulator’s 361-bit description; a separately initialized test movie costs 121 bits with the pair held. Both savings exceed 128 bits.

The observation task changes if the observer must instead predict whether each triple contains *any* occupied cell, the Boolean OR readout. Equal coarse neighborhoods then have unequal successors, so none of the 256 elementary rules reproduces every observed transition. The rule minimizing one-step error produces the divergent reconstruction in Figure 6e; correcting it costs more than the microscopic baseline. This declared model search fails the certificate. Models using additional state, history, or a different coding method remain possible. Appendix B.2 supplies the search, the second coding account, and the fresh-run test. These examples make acquisition and its finite checks concrete; the theorems establish the limits of uniform discovery guarantees.

### 5.4. Why Discovery Can Fail

The certificate allows real discoveries, including repetition. It does not provide a method guaranteed to find them with the observations available. Consider a stream of fixed-size images that repeats a finite movie. A compact program may generate that movie and its repetitions, yet a given total method can fail to find a shortening of the movie it has received.

**Proposition 2** (Discovery and regret barriers for reusable models)**.**
*Fix positive total margins $\delta_{\mathrm{comp}}$ and $\delta_{\mathrm{test}}$. Let the modeling domain include the periodic-block family of Appendix D.6, with fixed chunk width b≥1, supplied block length m, and literal baseline bm. Let A be a total computable constructor that always returns a valid candidate: an admissible pair whose encoder and decoder are total on the declared domain and reconstruct its retained data.*

(a) Discovery. *For every sufficiently large m, there is an m-chunk observation block for which a pair satisfies Definition 3, but A returns a complete retained code of length at least bm and, therefore, fails (iii).*
(b) Regret. *There is a constant $c_{A}$, independent of m, such that the best qualifying complete code on these instances has length at most $c_{A}$. The excess length returned by A is consequently at least $bm-c_{A}$, and is unbounded.*

**Proof** **idea.** There are $2^{bm}$ possible observation blocks. Validity makes their complete codes distinct, but only $2^{bm}-1$ binary strings have length below bm. Some block is, therefore, left unshortened. A fixed program containing *A* can find the first such block by finite search, given *m*, and replay it. Its code cost is independent of *m*. A match flag encodes each predicted block; an escape flag followed by literal data keeps the code valid on other blocks. For sufficiently large *m*, this model passes both fixed margins. Appendix D.6 gives the complete construction and accounting.    □

The short model may be extremely slow: this is a description-length result with no runtime deadline. Storing the block literally and replaying it is possible after the first copy, but does not yet save bits once storage is charged. Further matching observations can repay that cost. The model constructed in the proof already meets the construction compression margin on the first copy and meets the prospective-reuse margin on the next copy, yet the given constructor misses it. Failure concerns the observations collected so far; the same method may recognize and exploit repetition later. The witness uses a supplied representation. That is enough to defeat a general joint guarantee, since a method required to handle the full family must handle this subclass. It does not establish a separate hardness result for choosing ρ.

**Problem 1** (Joint construction in restricted families)**.**
*Under which effective restrictions can one terminating algorithm find a relevant representation and a reusable compressive model whenever a qualifying pair exists? Any pair meeting Definition 3 suffices; optimality is not required.*

*Each finite instance w declares the construction inputs, prior frame, objective and relevance requirements, admissible representation and model classes, baselines, margins, and evaluation protocol. It specifies the effective access to those requirements; test observations arrive only after selection. Characterize families on which the same total computable constructor A(w) always returns a valid candidate, and returns a qualifying pair whenever one exists with the available construction observations and declared continuation. Candidate classes may be infinite. Where a guarantee fails, identify the obstruction; restrictions on future continuations and effective stopping rules are part of the problem.*

Section 6 explains why omitted information can matter to later modeling. Section 7 examines structures that can make the joint search manageable.

## 6. Conserved Information and the Choice of Coarse-Graining

What a representation omits does not thereby leave the substrate. Under reversible microscopic dynamics, complete-state algorithmic complexity is conserved up to fixed coding constants, with the law and elapsed time supplied. Detail omitted now can reach the retained variables later. The retained variables, therefore, need not stay simple, and need not determine their own future. Both bear on representation choice, because a useful projection has to support modeling across time, not only at one instant.

Let $\Omega_{t}$ be a complete finite encoded state, and assume that, at the declared resolution, its dynamics are a computable bijection. Let $\rho_{\Omega }$ be a computable projection on these states and write $Q_{t}^{\rho }:=\rho_{\Omega }\left(\Omega_{t}\right)$. A self-delimiting residual index $E_{t}^{\rho }$ identifies $\Omega_{t}$ within the finite set of states having the same projected value $Q_{t}^{\rho }$. Its length may vary between these sets. Unlike the records used in model acquisition, these variables describe one instant.

Dropping the residual makes the representation lossy; it does not by itself make the retained information easier to model.

*Complete-state conservation and localization.* Write the fixed reversible evolution as

(41) $$\Omega_{t+T}=F^{T}\left(\Omega_{t}\right),$$

for a supplied signed offset *T* and computable maps *F* and $F^{-1}$. Fix ${\tilde{C}}_{\rho }=\left(C,F,T,\rho_{\Omega }\right)$. Reversible invariance then gives

(42) $$K\left(\Omega_{t+T}|{\tilde{C}}_{\rho }\right)=K\left(\Omega_{t}|{\tilde{C}}_{\rho }\right)+O\left(1\right).$$

The dynamics may unfold and redistribute the complete description, but do not create new algorithmic information that grows with system size  [13,41].

At the declared finite resolution, the forward map $\Omega_{t}\mapsto \left(Q_{t}^{\rho },E_{t}^{\rho }\right)$ and its inverse form a computable reversible *self-delimiting representation*,

(43) $$\Omega_{t}\;\overset{\pi_{\rho }}{⟷}\;\langle Q_{t}^{\rho },E_{t}^{\rho }\rangle .$$

The allowed residual codes may, therefore, depend on $Q_{t}^{\rho }$; we do not assume one fixed residual alphabet for every projected value. The actual coarse-graining is the subsequent lossy operation that retains $Q_{t}^{\rho }$ and omits $E_{t}^{\rho }$. With the same ${\tilde{C}}_{\rho }$ held fixed,

(44) $$\begin{array}{cc} K(\Omega_{t}|{\tilde{C}}_{\rho }) & =K\left(Q_{t}^{\rho }|{\tilde{C}}_{\rho }\right)+K\left(E_{t}^{\rho }|{\tilde{C}}_{\rho }\right)\\ & \;-I_{K}\left(Q_{t}^{\rho }:E_{t}^{\rho }|{\tilde{C}}_{\rho }\right)+O\left(1\right). \end{array}$$

Subtracting the same identity at two times and using Equation (42) gives the localization balance, where Δ denotes the change between the two times,

(45) $$\Delta K\left(Q^{\rho }\right)+\Delta K\left(E^{\rho }\right)-\Delta I_{K}\left(Q^{\rho }:E^{\rho }\right)=O\left(1\right),$$

with the common conditioning suppressed for readability.

This is the sense in which a projected macrocoordinate can become more complex while the global budget is conserved: description already present in the complete state can be relocalized into the retained kernel, the residual, and their shared algorithmic structure. Two difficulties follow for the observer, neither of them a failure of the accounting. First, the retained coordinate can gain description length as detail once held in the residual becomes visible in it. Second, and independently, the projection can discard predictive distinctions: two complete states sharing a retained value may evolve to different retained values, so no deterministic update on the retained value alone reproduces both. The observer must then enlarge the representation, condition on more of the history, or describe the retained variables probabilistically. This is a failure of the exact closure condition $\rho \circ D=\bar{D}\circ \rho$ of Section 4.2; Section 7 exhibits structures under which it holds. Neither effect amounts to information creation by the projection:

(46) $$K\left(Q_{t}^{\rho }|{\tilde{C}}_{\rho }\right)\leq K\left(\Omega_{t}|{\tilde{C}}_{\rho }\right)+O\left(1\right).$$

The fuller coordinate decomposition and exact localization ledger are developed in the companion paper [41]; they are not needed for the construction results here.

*A minimal relocalization picture.* Consider a reversible cellular automaton whose block-averaged initial field is simple while the within-block arrangement is incompressible. Most of $K(\Omega_{0}|{\tilde{C}}_{\rho })$ then resides in $E_{0}^{\rho }$. Under a mixing reversible rule, fine-scale differences can later affect the retained block field, so bits initially localized in the residual become necessary to specify $Q_{t}^{\rho }$. Equation (45) accounts for this transfer without creating information. Detail omitted at t=0 can therefore contribute to the description complexity of the later retained field; simplicity of the initial field alone gives no guarantee of an economical description across time. Theorem 1 shows how detail omitted from an initial readout can become visible across an observed history: the instantaneous readout discards all but one bit at every step, yet the history it generates reproduces the entire initial state. Each readout remains simple; the accumulated history carries the complexity. Conversely, if the seed is simple conditional on the law, then $K\left(\Omega_{t}|{\tilde{C}}_{\rho },t\right)=O\left(1\right)$ for every *t*; apparent macro-level richness is only the unfolding of a short program.

*From states to observed histories.* The balance above concerns instantaneous states; model acquisition concerns records accumulated through time. A useful projection must preserve the distinctions required by the objective across that history. Omission can reduce what must be modeled; the certificate requires additional savings within the retained information, after charging the representation and model. Section 7 examines the physical structures that make such a pair accessible: symmetry, timescale separation, and closure.

## 7. Macromodel Discovery in Favorable Systems

Physical structure can make useful compression possible and make its discovery tractable. Constraints on realizations and task-relevant omission reduce what must be described. Symmetries, restricted model classes, and experiments can then guide an effective search. These are separate requirements: an economical description may exist without being accessible to the observer’s available procedure. The following cases identify sources of structure relevant to the restricted joint construction asked for in Problem 1.

For the search, it is useful to separate *inherited microstructure* from *bridge structure*. Symmetries, conservation laws, locality, and interaction constraints may be computably recoverable from the microlaw and can sharply shrink the admissible macro-model class without adding substantial conditional description cost. The projection, order parameter, scaling regime, timescale separation, and closure that connect levels are often the difficult part. Empirical data can then supply constitutive laws or parameters not fixed by the microscopic description.

If $a_{\mathrm{bridge}}$ denotes whatever bridge information is not already available from the conditioned microscopic description and a fixed construction Φ on a restricted class satisfies

(47) $$\left(\rho ,M\right)=\Phi \left(D,a_{\mathrm{bridge}}\right),$$

then

(48) $$K\left(\rho ,M|D,C\right)\leq K\left(a_{\mathrm{bridge}}|D,C\right)+O\left(1\right).$$

Microscopic knowledge can reduce description and search cost without itself being a uniform constructor. The split is frame-relative: if some component of $a_{\mathrm{bridge}}$ is derived from D or learned earlier, it moves into the conditioned background *C*.

*Symmetry and restricted coarse dynamics.* Anderson’s positive lesson is that microscopic symmetry is not discarded at the higher level: it constrains admissible phases, order parameters, and collective modes  [14]. His ammonia and crystal examples also show what symmetry does not do: the microscopic Hamiltonian can constrain the possible sectors without selecting the realized symmetry-broken organization or the variables in which it is simplest. Israeli and Goldenfeld give a constructive discrete analog: within a restricted cellular-automaton search they identify block maps whose projected variables evolve by a simpler rule of their own, with no reference to the discarded detail  [33,42]. This construction succeeds within the specified search class. Figure 6 shows the rule-146 example and a failed projection for the same microscopic run.

*Particles to fluids.* Newtonian mechanics and local conservation constrain continuum balance equations but do not by themselves close them: stresses, heat fluxes, equations of state, and transport coefficients require constitutive information. A kinetic description connects particle dynamics to hydrodynamic variables; constitutive relations then close the balance equations into fluid equations. In controlled scaling regimes, the Boltzmann equation yields Euler and Navier–Stokes limits [43]; once the relevant variables and regime are identified, kinetic and statistical mechanics can also relate macroscopic coefficients to microscopic interactions. Projection methods explain the qualification: exact elimination of unresolved variables generally produces memory and fluctuating terms rather than an autonomous macrolaw [44]. Scale separation, weak memory, local equilibrium, or a restricted constitutive family are the favorable structures that make closure possible.

*Microlaws constrain the joint search over (ρ,M).* In favorable systems, exact symmetries, conservation laws, interaction topology, analytic structure, or scale hierarchy first restrict plausible projections ρ. One then derives the evolution or statistical weight of those projected variables. If unresolved variables remain, closure has failed: the representation must be enlarged, an approximation justified, or another scaling regime chosen. Exact closure, self-averaging, or renormalization-group irrelevance can instead yield a closed equation or effective Hamiltonian *M*. The following examples realize different versions of this route: ρ identifies the retained kernel, unresolved coordinates become $E^{\rho }$, and the closed law supplies *M*.

*Exact quotient: Curie–Weiss.* For *N* spins $s_{i}\in \left\{-1,1\right\}$, the mean-field Ising Hamiltonian $H_{N}$ and magnetization *m* are

(49) $$H_{N}\left(s\right)=-\frac{J}{2N}{\left(\sum_{i=1}^{N}s_{i}\right)}^{2}-h\sum_{i=1}^{N}s_{i},\;m\left(s\right)=\frac{1}{N}\sum_{i=1}^{N}s_{i},$$

where *J* is the coupling strength and *h* the external field; *J*, *h*, *m*, and the inverse temperature β below are local to the Ising examples. The Hamiltonian factors exactly through the magnetization,

(50) $$H_{N}\left(s\right)=-N\left(\frac{J}{2}m^{2}+hm\right).$$

The all-to-all coupling and permutation symmetry, therefore, do more than suggest *m*: for energetic purposes they identify an exact quotient ρ:s↦m. Combinatorial counting gives the multiplicity of each magnetization class, and the thermodynamic limit turns energy plus multiplicity into a one-dimensional free-energy problem whose stationary condition yields the mean-field self-consistency law at inverse temperature β,

(51) $$m=\tanh\left[\beta (Jm+h)\right].$$

Here the microlaw removes almost the entire representation search: symmetry identifies ρ:s↦m, the microscopic arrangement within a fixed-*m* fiber is the residual $E^{\rho }$, combinatorial counting measures that residual, and the large-*N* limit supplies *M*, the self-consistency law above [45].

*Asymptotic relevance: short-range Ising and renormalization.* The previous factorization fails for the nearest-neighbor Ising model. Its Hamiltonian depends on local pair structure, so microscopic configurations with the same global magnetization can have different energies. It nevertheless supports macroscopic laws. At zero field, the $Z_{2}$ spin-flip symmetry singles out magnetization as the natural symmetry-breaking order parameter; translation symmetry and locality motivate block magnetizations, correlation fields, and long-wavelength modes rather than arbitrary functions of the $2^{N}$ spin configurations.

Renormalization then turns this guidance into a construction procedure: coarse-grain the microscopic field, integrate out short-scale variables, and follow the induced flow of the effective couplings. Symmetry restricts which operators may appear, locality organizes their form, and spatial dimension determines which couplings are relevant, marginal, or irrelevant. Near a fixed point, most microscopic couplings die away while a small set of relevant directions controls phase structure, scaling laws, and critical exponents [46,47]. Thus ρ need not be an exact finite-scale quotient to support macroscopic laws: the microlaw can instead identify a representation whose discarded coordinates become asymptotically irrelevant. The renormalization-group flow identifies which operators continue to affect long-scale behavior and which become irrelevant, with the fixed-point theory supplying *M*.

*Exact dynamical closure in a neuronal population.* Montbrió, Pazó, and Roxin provide a dynamical counterpart  [48]. For a globally coupled heterogeneous population of quadratic integrate-and-fire neurons, the microscopic equations first remove neuronal labels from the interaction: in the thermodynamic limit, the natural object is a population voltage density rather than *N* independent trajectories. On the invariant Lorentzian-ansatz family, instantaneous coupling and Lorentzian heterogeneity allow the population integral to close exactly by analytic continuation. Writing $f_{t}$ for the continuum population-density state, the resulting projection

(52) $$\rho :f_{t}⟼\left(r\left(t\right),v\left(t\right)\right)$$

retains the population firing rate *r* and mean membrane potential *v* (in the principal-value sense), whose evolution obeys a closed pair of ordinary differential equations. Finite populations provide empirical estimates of these continuum observables. Firing rate alone is not sufficient for all collective states; failure of that smaller representation is what forces the additional macrovariable *v*. In the language used here, global coupling and analytic structure narrow the candidate ρ, the closure calculation tests whether the omitted microscopic coordinates can indeed be relegated to $E^{\rho }$, and the exact reduced equations are *M*.

These cases illustrate three distinct ways microscopic information can make macromodel discovery tractable: an *exact quotient* can collapse the representation space immediately; a *controlled asymptotic limit* can make discarded microscopic distinctions irrelevant; or an *invariant/analytic closure* can identify the minimal set of collective variables needed for autonomous dynamics. These are mechanisms for discovering representations and laws; satisfying Definition 3 additionally requires the declared compression margins and held-out reuse. None is a universal constructor. What they show is more specific: favorable microlaws can encode strong clues about *which representation to try* and can provide mathematical tests of whether the proposed representation supports a closed macrolaw.

*Hybrid and macro-first construction.* Hodgkin and Huxley combined membrane-level charge and circuit constraints with a mesoscopic representation and empirically inferred ionic conductances and gating kinetics  [49]. Their model is neither “from microphysics” nor “from data alone.” Thermodynamics supplies the converse historical route, with stable macrolaws preceding their statistical-mechanical explanation. Reduction is, therefore, usually a ladder of effective levels, and science may enter that ladder from above, below, or both.

These examples suggest the positive scientific counterpart to the theorems: *constrained cumulative search*. Science does not enumerate all programs. It inherits representations and laws, uses experiments and interventions to discriminate candidates, restricts attention to model classes that have worked before, and retains structures that continue to compress new data under a stable parameterization. A restricted class makes the search manageable when, for example, it is finite and effectively enumerable or has a computable stopping rule.

Within such a search, the observer can certify a relative optimum. Fix ρ, the modeling task and coding convention, a candidate class C, and a computable score *L*. If every candidate in C can be checked and a computable rule determines when the search is complete, then the observer can certify

(53) $$M_{C}^{*}\in \underset{M\in C}{\mathrm{arg min}}\;L\left(M\right).$$

This is optimality only within (ρ,C). Successful models then become part of the next observer frame *C*, shrinking later searches. The success of this cumulative process depends on which representations the observer inherits, encounters, and can test.

## 8. Discussion

Constructing a reusable model, computing answers from it, and using it to act impose different requirements. These distinctions explain how the construction barriers coexist with successful scientific modeling and how they relate to other accounts of emergence.

An acquired model’s implementation is judged against the agent’s objectives. For telehomeostatic agents, models support regulation that sustains the persistence of the agent or its kind, so predictive accuracy, response time, and resource demands matter through their effects on that objective [13]. A shortest program can be too slow for a required action, while a larger equivalent program may meet the deadline. Evolution or learning can favor the additional stored structure when its benefit outweighs its cost. The compression certificate tests coding savings after acquisition costs; adequacy for the task and effective planning are separate requirements for successful action (Section 8.3).

### 8.1. Construction and Use: Distinct Computational Limits

We distinguish *constructing* a model from *using* one. Construction asks whether reusable structure exists in the chosen representation and whether an agent can find it. Use asks how a declared question *q* can be answered from the microscopic description or from an already acquired model. These problems can fail for different reasons. In particular, description length and runtime are separate axes. The shift-register example has a history that can remain incompressible even though a bit at a specified future time is cheap to obtain. Conversely, a system started from a short generative seed may have a compact description while answering a far-future question may still require running the dynamics.

Evaluation may have a computable stopping criterion, such as a supplied finite horizon or a finite-state cycle certificate, or be open-ended. This concerns answering *q*, not coding the finite record. Box 1 distinguishes construction failures from answer-side undecidability.

Box 1 **Diagnostic situations in the micro-to-macro problem.**

*No substantial lossless compression of the retained history.* Even for a finite micro-experiment, the retained history may have no substantial compression gap (Theorem 1).

*Every total model builder has blind spots.* Each fixed total valid description method leaves some records unshortened at every sufficiently large length, even though they have logarithmic-length descriptions (Proposition 1). The discovery and fixed-regret failures also hold for complete codes under the compression certificate, with the observations available so far (Proposition 2).

*No computable macro map for a chosen variable.* With open-ended computation and fixed relevant (ρ,q), there may be no algorithm that returns the requested answer for every microscopic specification in the family, as in the Ising-observable and phase-diagram constructions  [50,51].

*No computable long-run answer inside a known model.* A compact update rule may be explicit and each finite step computable, while its eventual state or fixed point remains undecidable, as in a Turing machine with an unbounded tape or the renormalization-group construction [52,53].

*When microscopic evaluation has a stopping rule.* For eventual-time queries on a deterministic finite reachable state graph, exact cycle detection supplies a stopping rule. Instance sizes and horizons may still grow without bound across the family. A compact continuous phase space need not be finite-state, and Poincaré recurrence does not, in general, imply exact periodicity; geometric boundedness alone, therefore, supplies no such rule (Appendix C.4). When evaluation terminates, the remaining question is whether the observer can avoid replaying the microscopic trajectory.

**Figure 7:**
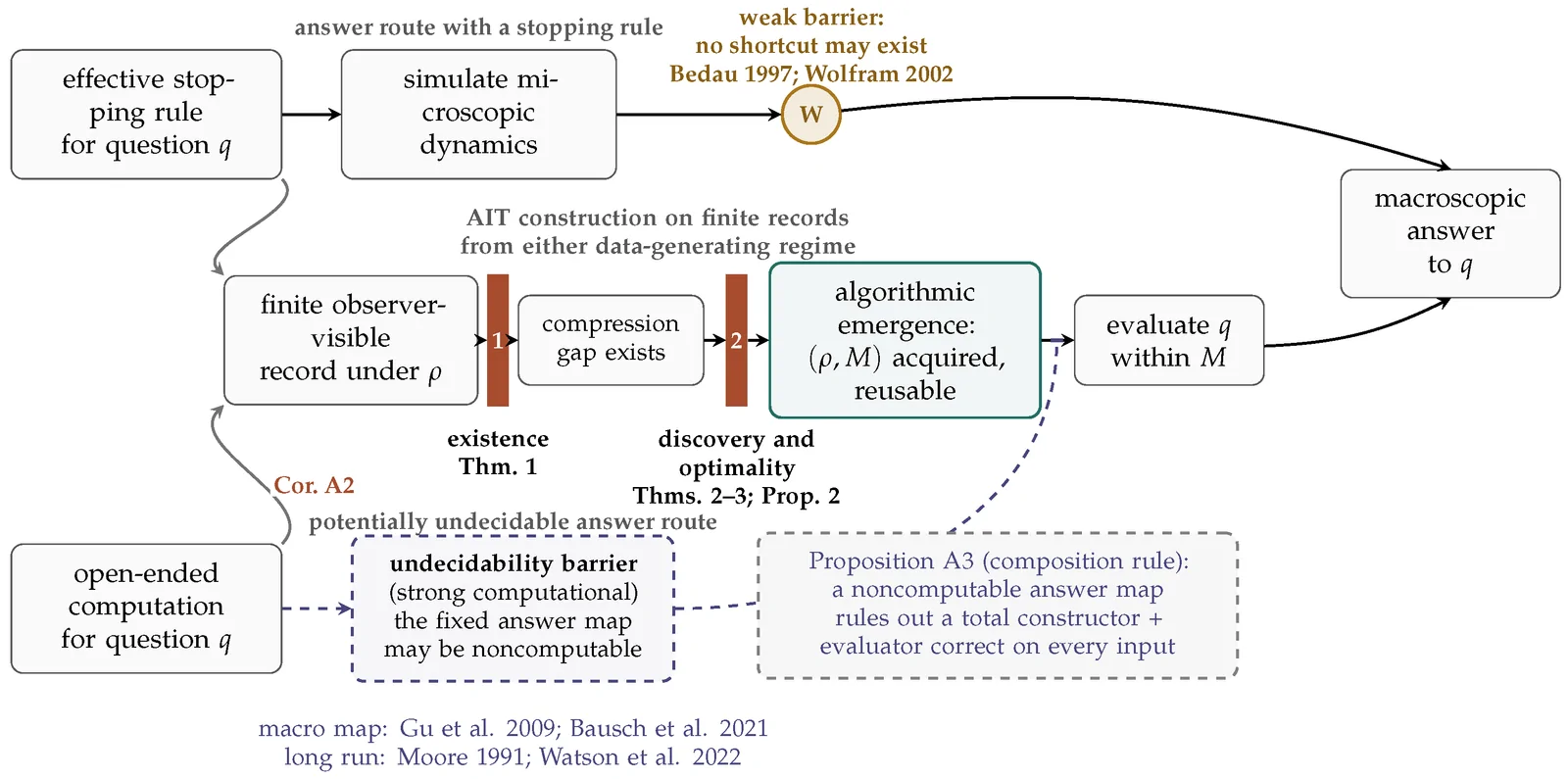
**Construction and answer barriers act on different coordinates.** The top route covers microscopic evaluation with an effective stopping rule; the gold marker denotes the possible weak-emergence toll that no shortcut exists [40,54]. A stopping rule is available when the target’s state carries bounded encoded information, so that its reachable state graph is finite, or when the question supplies a horizon. The middle route starts from a finite observer-visible record supplied by either data-generating regime. The rust barriers mark failure of a substantial lossless compression gap to exist (1) and failure of universal discovery or near-optimality guarantees (2); the green node marks successful algorithmic emergence. The gray curved arrows show that finite observations from either regime enter the same construction problem. The lower arrow is labeled by Corollary A2, which transfers the barriers through suitable record-preserving embeddings. The bottom route covers open-ended computation, whose answer map may be noncomputable, producing the undecidability barrier [50,51,52,53]. Unbounded encoded information, an infinite lattice, growing memory, or a continuum state, is what makes that possible. The dashed implication is Proposition A3, a logical implication rather than an evaluation route.

Bedau calls a macrostate weakly emergent if it is derivable from the system’s microdynamics and external conditions, but only by simulation. His “macrostate” can also denote a property or pattern of behavior. Wolfram’s principle of computational irreducibility gives the corresponding no-shortcut thesis for rule-based systems: predicting the state at horizon *t* may require work equivalent to running the evolution to *t* [40,54]. In this terminating case the obstruction is computational cost, not the absence of an answer. Neither formulation by itself proves a complexity lower bound for every rule and every macroscopic question. Figure 7 separates possible evaluation routes from the limits on universal construction and answering guarantees.

Acquiring (ρ,M) gives the observer an alternative route to answers within the model’s domain, through autonomous coarse dynamics, a closed relation, or an effective law for the retained variables.

Representation couples description length and runtime. Section 7 discusses Israeli and Goldenfeld’s coarse-grainings that restore predictive shortcuts for retained questions in computationally irreducible automata [33,42]; Figure 6 illustrates one exact coarse law. Section 6 gives the information accounting: with the law and elapsed time supplied, reversible dynamics conserves complete-state complexity up to coding constants while redistributing information. The projection ρ determines which distinctions remain visible and available for prediction; computational irreducibility is, therefore, relative to the variables being tracked. The barriers proved here concern compression-certified construction, not the runtime of what is constructed.

*When the answer computation is open-ended.* An answer computation is open-ended when *q* depends on memory, lattice size, or elapsed time for which no finite cutoff is supplied. Such a model can encode the halting problem. When the problem specifies a total answer map and that map is noncomputable, no total algorithm realizes it on every input. This *undecidability barrier*, labeled the strong computational barrier in Figure 7, concerns the absence of a uniform algorithm rather than high computational cost. The open-ended resource makes the obstruction possible but does not imply it.

*Physical realizations of the undecidability barrier.* Open-ended computation need not require an unbounded geometric domain. Moore showed that smooth finite-dimensional dynamics, including a particle moving in a three-dimensional potential, can simulate a universal Turing machine [52,55].

More recent constructions realize Turing-complete stationary Euler and Navier–Stokes flows on compact or suitably metrized manifolds [56,57]. These fluid results concern Lagrangian reachability, not undecidability of Navier–Stokes singularity formation; Tao’s proposed time-dependent Eulerian fluid-computation route to blow-up is related but distinct [58,59]. Appendix C.4 explains how compact continua evade finite-state cycle detection and why Poincaré recurrence does not restore it. Elementary cellular automata provide a discrete universal counterpart [60].

Physical many-body systems realize the same distinction. Gu et al. encode cellular-automaton evolution in the ground states of an infinite periodic Ising lattice and obtain averaging observables that cannot be computed uniformly from the microscopic specification [50]. The infinite lattice is essential; fixed finite systems remain computable in principle. Spectral-gap undecidability likewise concerns the thermodynamic limit [61]; the obstruction persists in one-dimensional spin chains, extends to phase diagrams, and survives rotational symmetry on the square lattice [51,62,63].

Watson, Onorati, and Cubitt construct an explicit renormalization-group map whose finite iterates are computable, while determining which fixed point the flow approaches is undecidable [53]; see [64] for a recent review.

*Finite-record construction persists in larger system classes.* A finite-horizon observation from an infinite or computationally open-ended system remains a finite record and is still subject to the AIT construction barriers. Corollary A2 makes this inheritance precise: any effectively encoded larger class admitting a computable, finite-horizon, record-preserving embedding of the finite experiments inherits all three barriers. For the residual-information bound, the embedded law, projection, horizon, and frame must remain common across the initial-state ensemble. A constructor universal on that larger class would otherwise solve the embedded finite hard family. Gu et al. fix a macroscopic variable and ask for its value, whereas the observer here seeks a reusable representation and compressed model. A larger substrate class can present both problems.

*Composition links construction to use.* For fixed ρ and *q*, suppose the formal problem assigns one answer to each microscopic input. If a total constructor always produced a suitable theory and a total evaluator always recovered that answer, their composition would compute the answer map. Proposition A3 states the implication: a noncomputable map forces at least one stage to fail to be total or universally correct. It does not identify the failing stage or exclude useful theories for individual systems. Appendix E gives the formal statement and its one-way scope.

The converse carries no such consequence. A computable answer need not arise from a compressed, reusable theory, and a useful theory need answer only questions in its declared domain. A constructor and evaluator may reproduce the microscopic computation. If the constructor may choose ρ without a relevance constraint, it can evade the question by discarding the distinction that *q* asks it to preserve.

### 8.2. Emergence as Model Discovery

The AIT barriers concern the existence and discovery of short descriptions; algorithmic emergence concerns an observer’s acquisition of a reusable compressive model. Acquiring a model is a different question from whether its predictions require simulation, whether all questions about its behavior are decidable, or whether higher-level facts follow from lower-level facts. Table 1 organizes the accounts by these questions and by their criteria for model structure and causal influence. These questions can intersect: an observer can acquire a useful model whose behavior still requires simulation or admits undecidable questions. Our proposal is, therefore, a separate epistemic criterion, not a refinement of weak emergence. Comparisons must specify the property and levels under consideration.

*Acquisition can make prediction cheaper.* A macroscopic model can replace microscopic simulation with direct calculation or with a cheaper simulation of the retained variables. For the glider in Section 5.3, position, phase, and orientation determine later observations without updating every cell. Acquisition can, therefore, yield both description savings and faster prediction. Other acquired models may still require step-by-step evolution. Bedau’s criterion concerns which derivations of a macrostate exist, rather than which the observer has discovered [54].

*An acquired model can retain computational barriers.* Suppose a coarse-graining yields a compact macroscopic rule that implements a universal Turing machine when its memory is allowed to grow without bound. If the observer acquires a representation–model pair based on this rule that passes Definition 3, algorithmic emergence has occurred. Yet no algorithm decides, for every input, whether this macroscopic machine will ever halt. The undecidability concerns unbounded computation; each fixed finite-horizon record remains computable. Thus model discovery can succeed while computational barriers remain within the discovered model.

*Observer-dependent emergence and acquired compression.* Abrahão and Zenil formalize observation through mutual perturbation between a system and an observer equipped with a formal theory [68]. Their observer-dependent emergence concerns trajectory information still needed beyond the observation, observer history, and fixed allowances for coding, observational error, and processing. Their criterion identifies an informational deficit relative to the observer’s theory; ours identifies an acquisition that supplies reusable compression. Our ρ specifies retained distinctions; observer–world interaction may be included in the dynamics. Theorem 1 shares the concern with information remaining after supplied knowledge, but uses different conditioning data.

An extension of the observer’s theory can remove their deficit by storing a literal answer, without yielding a shorter or reusable description. Conversely, a model can compress a selected component without eliminating the deficit for the full trajectory. Prospective reuse is therefore a different requirement, not a stronger version of their criterion. Their asymptotic notion fixes an evolving system and eventually exceeds each fixed observer’s informational resources. Proposition 1 instead fixes a total valid description method and finds simple records that it leaves unshortened at each sufficiently large length. No implication between these claims is asserted. Their reading of Bedau emphasizes informational incompressibility; here description length and the computation required to use a description remain separate.

*Model structure and model acquisition.* Bédard and Bergeron [15] define emergence through several minimal partial models of a record, distinguished by drops in a modified structure function. Their closing discussion relates scientific investigation to successive improvements in models, while emphasizing that optimal models cannot be computed from data, in general. Their formal definition characterizes models for a record; ours concerns an observer’s transition to a usable model. Our certificate (Definition 3) tests acquisition through task relevance, actual implementation cost, a declared baseline, and later coding reuse; it does not require computing a structure function or identifying a minimal model. Section 4.1 uses the ordinary structure function and the weaker net-gain diagnostic $G_{x}$. Earlier KT work treats emergence as a resource-limited agent’s discovery of useful coarse-grainings [20]. A drop from microscopic to macroscopic description length can reflect omission alone and does not by itself certify reusable regularity.

Compression is also the common ground with Gell-Mann and Lloyd’s effective complexity, which measures the description length of a record’s regularities [66]. Here the additional question is when an observer acquires a model that captures and reuses regularities.

*Causal emergence and acquired models.* When the representation and model reproduce the effects of the specified interventions (Section 4.2), the compression-and-reuse certificate can record the acquisition of a causal macroscopic model. Hoel and colleagues define causal emergence by an increase in effective information at a macro level: coarse-graining can reduce noise or degeneracy in the induced transitions enough to outweigh the smaller state space  [67]. Acquiring a causal macromodel makes this comparison available; the compression certificate does not determine its outcome.

Reversible dynamics give a case in which the criteria separate. For a complete finite microscopic state space with *N* states, a deterministic reversible update has effective information $\log_{2}N$ bits under Hoel et al.’s uniform interventions. A coarse-graining with m<N macrostates has at most $\log_{2}m$ bits, so it cannot increase this measure. An observer may nevertheless discover a compact, reusable model of its retained observations that passes Definition 3. The acquisition changes the observer’s descriptive resources while the microscopic dynamics remain information preserving. Algorithmic emergence thus requires neither information loss in the microscopic evolution nor an increase in effective information at the macro level.

*Existence, entailment, and construction.* Chalmers asks whether higher-level truths follow from the complete lower-level facts, interpreting *follow* as conceptual or metaphysical necessitation; strong emergence is a failure of this relation even in principle [65]. Of the two algorithmic tasks, answering a fixed macroscopic question is closer in form to Chalmers’s question than constructing a reusable model. This comparison concerns the questions asked, not an implication from undecidability to strong emergence. Necessitation is distinct from provability in a specified formal theory and from uniform effective construction of an answer or model. Under classical semantics, truth in an intended mathematical structure differs from consequence of a specified theory. In classical first-order logic, consequence across all models of the axioms and formal provability coincide by soundness and completeness. An arithmetic statement can nevertheless be true in the standard natural numbers yet unprovable from a given sound, effectively axiomatized arithmetic theory. Likewise, every finite string has a shortest program, and hence a definite K(x), although no algorithm computes it for every string. Neither observation by itself establishes a failure of metaphysical determination.

Constructive approaches give a different meaning to truth and existence  [69]. On an intuitionistic proof interpretation, the meaning of a proposition is given by what counts as a proof: an existential proof supplies a witness and a proof that it satisfies the asserted property, while a proof of an implication transforms proofs of its premise into proofs of its conclusion ([70] pp. 412–414). Where these constructions are interpreted computationally, a uniform existence proof must supply a witness-producing procedure from the specified inputs and evidence. Intuitionism does not by itself equate construction with Turing computability, and the absence of a uniform procedure does not preclude constructions in particular cases.

Knowing how to generate each finite stage of a microscopic process does not, in general, provide a uniform procedure for settling macroscopic questions about its unbounded behavior across all inputs. A classical realist can nevertheless regard each such proposition as already true or false; an intuitionistic proof interpretation need not assign it that proof-independent status. Forster, Kunze, and Lauermann’s constructive formalization treats complexity as a relation between objects and description lengths, rather than as a computable numerical function [71]; Appendix C.1 explains the description and compression witnesses.

Direct simulation constructs each finite observed record; an economical, reusable model is a further construction. Proposition 2 separates individual availability from uniform construction over the stated periodic-block family: no total computable constructor returning valid candidates guarantees finding a qualifying pair whenever one exists, even with the representation and block length supplied and no runtime deadline. Microscopic facts may fix the record without supplying a general effective route to a compact, reusable model. Algorithmic emergence concerns the observer’s acquisition of that model, giving Anderson’s distinction between reduction and construction an operational form.

*Counting and diagonal arguments.* The barrier theorems rest on two mechanisms. Theorem 1 is a counting argument and takes no diagonal step. Theorems 2 and 3 and Proposition 1 are diagonal arguments: they reduce to the halting theorem or use Berry-type constructions, and Proposition 2 carries their obstruction to representation–model pairs. Lawvere’s fixed-point theorem gives that diagonal family its common abstract form, shared with Cantor’s theorem and the halting, Gödel, and Tarski arguments [72,73]. In its set-theoretic form, if a family indexed by a set represents every function from that set to an output set, every self-map of the output set must have a fixed point. Boolean negation has none. Applying a represented function to its own index and negating the result, therefore, defeats the proposed universal representation.

Wolpert’s physical-inference limits use the same diagonal mechanism [74]. A device weakly infers a quantity when, for each question whether it has a specified value, some setup guarantees a correct answer in every possible world compatible with that setup. The device belongs to the universe it describes, so its own binary conclusion is one such quantity. Asking whether that conclusion is “no” requires the answer to equal its own negation. No setup can meet this requirement, so no device weakly infers every binary function of its universe.

The two-device result closes the same loop. Mutual weak inference would allow one device to reproduce the other’s conclusion and the other to negate the first’s. If every pair of setups can coexist (Wolpert’s setup-distinguishability), these requirements must hold together, again forcing a conclusion to equal its own negation. Thus two such devices cannot weakly infer one another. Both arguments turn on the absence of a fixed point of negation, the obstruction expressed by Lawvere’s theorem, and require neither Turing-computable devices nor infinitely many possible worlds.

The connection to algorithmic information theory also holds by reduction. Computing exact Kolmogorov complexity for a fixed optimal universal machine would allow one to decide halting. Consequently, the uncomputability of *K* follows from the halting theorem and hence, through this reduction, from Lawvere’s diagonal framework [75].

Berry-type arguments make the description-length accounting explicit. For a fixed sound, effectively axiomatized theory, Chaitin’s certification ceiling follows by searching its theorems: a proof that a specified string has sufficiently high complexity would enable a shorter search program to find that string, contradicting the bound  [76]. In Proposition 1, the fixed total method returning valid descriptions and the length *n* specify the first *n*-bit record the method leaves unshortened. This gives the record a description of length O(logn), with a method-dependent constant. Theorems 2 and 3 use this reasoning or reductions to it; Proposition 2 transfers the obstruction to qualifying representation–model pairs within the stated periodic-block family. The constants and additive gaps require the explicit encoding estimates supplied by these proofs.

Li presented an index-based Berry construction within Lawvere’s framework ([77] p. 500), and Bauer obtains the Kleene–Rogers recursion theorem through a multivalued extension [78].

Counting supplies a complementary argument. With background knowledge fixed, there are $2^{n}$ binary records of length *n*, but fewer than $2^{n-k}$ programs shorter than n−k bits, for integers 1≤k≤n. Fewer than a fraction $2^{-k}$ of those records can, therefore, have such short descriptions. This finite fact is the whole mechanism of Theorem 1.

Neither counting nor diagonalization requires infinity. Unboundedness enters the applications separately. The halting theorem concerns arbitrary finite programs whose available memory and execution time have no fixed ceiling. Each terminating computation nevertheless uses only finitely much of both. A deterministic machine with a known finite configuration space admits a halting test by simulation until termination or repetition. Gödel’s and Tarski’s arithmetic results concern the unbounded natural numbers and families of finite formulas; no individual formula or proof is infinitely long. Chaitin’s ceiling similarly compares a finitely specified theory with claims of arbitrarily high complexity.

Physical undecidability constructions encode unbounded computation in the observed model. Gu and colleagues use an infinite periodic lattice, while spectral-gap undecidability concerns the thermodynamic limit [50,61]. The continuum examples discussed earlier show why bounded spatial extent does not imply a finite configuration space.

In our construction barriers, every observed experiment, horizon, and record is finite. The unboundedness lies in the family over which one constructor must provide its guarantee. The observer’s program is finite, while its working memory has no fixed ceiling; this does not make any observed experiment infinite. Classically, a table of shortest descriptions exists for every fixed description frame and maximum record length, but no computable procedure generates these tables uniformly as that maximum grows.

The common diagonal mechanism thus connects the limitations, while their assumptions determine which guarantee fails: universal inference, a uniform answer, a shortening, a qualifying model, or a fixed additive gap. None of these arguments alone establishes that a macrostate requires simulation or fails to follow from complete lower-level facts.

### 8.3. Restricted Discovery, Action, and Inherited Structure

Restricted criteria and prior knowledge can guide the search for useful models. Successful action then depends on what those models predict and on the policies the agent can find.

Computational mechanics builds macrovariables by quotienting histories with identical predictive futures  [32]. Causal-emergence methods select macroscales that maximize an interventionist effective-information criterion  [67], while information-theoretic methods can identify relevant variables at renormalization-group fixed points [79]. These approaches optimize a specified predictive, causal, or scale-dependent criterion within a restricted class. They show how task relevance and restricted structure can guide parts of the joint search in Problem 1; a complete acquisition must also meet the certificate’s compression and reuse requirements. Zenil and colleagues’ perturbation-based decompositions provide another method for proposing generative models [39]. Connecting perturbations of a description to physical interventions requires additional modeling assumptions or evidence.

A complementary boundary case is model universality. De les Coves and Cubitt showed that the two-dimensional Ising model with fields can emulate arbitrary finite classical spin models, and Reinhart, Engel, and De les Coves formalize such emulations as efficient, modular transformations  [80,81]. This is a forward compiler once the target theory is specified; it does not infer from microscopic data which representation and macromodel are relevant. Target-specific information remains in the programmed simulator, so universal expressivity does not collapse the discovery problem.

*Models, planning, and successful action.* To help minimize OF through action, a model must predict the consequences of the actions being considered. The objective must also be attainable using the retained information and available actions, and an effective planner must find a satisfactory policy. In finite-horizon Markov decision processes, simulation lemmas bound policy-value errors using errors in the transition and reward models [82,83]. A performance guarantee then needs a margin covering both model error and planning error.

The converse asks when a successful controller can yield a reusable model of the task-relevant dynamics. Selecting actions and predicting observations are different interfaces; the same policy can succeed in systems with different dynamics. Recovering a model, therefore, needs further assumptions about the tasks, observations, or interventions that distinguish those systems. This remains a question for future work; successful control alone does not supply the compression certificate.

The prior frame itself may contain structure inherited through evolution, development, and scientific culture. The filter-of-time hypothesis in *Pattern, Persist!* proposes that surviving agent–world organizations are enriched for representations and models useful for persistence and regulation [13]. This is an evolutionary hypothesis, not a consequence of the construction barriers; it guarantees neither optimality nor transfer beyond the selecting environments.

Construction, evaluation, and successful action impose distinct requirements. These distinctions apply to scientific discovery and to cognitive model acquisition; science makes the construction problem unusually explicit.

## 9. Conclusions

Knowing the microscopic law and the information specifying a case can suffice to generate its observed history. It does not by itself yield a reusable higher-level model. Theorem 1 shows that even simple known dynamics and coarse-grained observation need not leave a substantial lossless shortening of the history. Every fixed total valid description method also leaves some records unshortened at each sufficiently large length, although they have logarithmic-length descriptions (Proposition 1). Open-ended search finds a shortening whenever one exists, but without a computable worst-case deadline. Allowing abstention does not restore unlimited exactness: a partial computable method correct whenever it answers can determine *K* on only finitely many distinct records (Theorem A2).

Algorithmic emergence is an event in the observer’s descriptive history: a relevant representation and reusable model make a previously unavailable compression usable. A pattern is what the model captures; discovery changes the available description, not the complexity of a fixed record. Compression connects these limits to discovery by making the reuse of regularity measurable. To meet the certificate, a representation must retain what the objective requires, and its model must save bits across the retained observations after acquisition costs, with further savings on new observations. Already for a stream that repeats one block, Proposition 2 shows that each total valid method can miss an already qualifying model with the observations available so far. The certificate is one-sided: passing it witnesses reusable regularity, while failing it leaves useful regularity unconfirmed. A short code for past data alone does not establish acquisition of a reusable model; the certificate, therefore, demands fixed coordinate roles and further savings on later observations. Repeated coding gains can repay a model’s fixed cost even when its implementation is large, so a regularity can be worth holding before that cost has been repaid.

The model’s functional core is a shortest implementation of the same declared behavior, with the same imperfections; that program need not be fastest or the best available predictor. For agents whose models serve telehomeostasis, a larger implementation can be preferable when it enables timely action.

Scientific practice makes progress by restricting the problem: physical constraints guide representation choices, experiments distinguish candidates, and successful models become resources for later searches. Under the reversible dynamics considered here, information omitted by a representation remains in the substrate and can reach the retained variables later. A useful projection must, therefore, preserve the distinctions required by the objective across time. The examples in Section 7 show how such restrictions can support construction.

A separate limit concerns answering a fixed macroscopic question. When a declared macroscopic answer map is noncomputable, no total computable constructor–evaluator pair can be correct on every input, even though useful models may exist for individual systems and questions.

Problem 1 asks when one terminating method can find a qualifying representation and model whenever such a pair exists in a restricted family. Further questions concern limits on predictive discovery under the same cost and algorithmic assumptions (Appendix B), and when acquiring and using a model is cheaper or supports more effective action than microscopic evaluation. These questions require accounting for description length, computation, repeated use, and the resources each acquisition adds to the observer’s frame.

## Figures and Tables

**Table 1:** Accounts of emergence and complexity, grouped by their defining questions. These questions concern logical derivation, computation, model structure, causal influence, or an observer’s knowledge. Several can apply to different aspects of the same system.

Account	Defining Question
*Derivation and computation*	
Strong emergence (Chalmers) [65]	Do higher-level facts fail to follow, even in principle, from complete lower-level facts?
Weak emergence (Bedau) [54]	Can a macrostate be derived from the microdynamics and the system’s external conditions only by simulation?
Physical undecidability [50,64]	Can one algorithm answer a specified physical question for every input in a family?
*Model structure and prediction*	
Minimal partial models (Bédard–Bergeron) [15]	Does the record’s modified structure function identify several minimal partial models?
Statistical complexity [32]	How much information is stored in the process’s optimal predictive causal states?
Effective complexity [66]	How long is a description of the identified regularities?
*Causal influence*	
Causal emergence [67]	Does a macro level have greater effective information than the micro level under specified interventions?
*Observer knowledge and acquisition*	
Observer-dependent emergence (Abrahão–Zenil) [68]	What trajectory information exceeds the observer’s knowledge and fixed allowances for coding, error, and processing?
Algorithmic emergence (this paper)	Has the observer acquired a reusable compressive model and a representation relevant to its objective?

## Data Availability

The example code, verifiers, recorded bit counts, and reproduction instructions are openly available in the companion repository https://github.com/giulioruffini/WP0007-algorithmic-emergence (accessed on 6 October 2026), at commit b63dd83 for this version. The Lean 4 formalization supporting the machine-checked results is openly available at https://github.com/giulioruffini/KTAIT (accessed on 6 October 2026). The core reductions behind the barrier results are formalized in Lean 4 in the KTAIT development, whose inventory and axiom audit are WP0195 [84]; Appendix F gives the declaration-level mapping and identifies the results proved only at paper level, including Proposition 2. Both repositories are cited in the Data Availability Statement.

## Appendix A. AIT Primer and Notation

This appendix states the AIT distinctions needed to read the paper and collects its notation. The formal definitions and theorem hypotheses in the body remain authoritative.

### Appendix A.1. AIT Assigns Description Lengths to Individual Finite Records

Fix a universal decoding procedure *U* whose valid programs form a prefix-free set, so a program is self-delimiting and concatenated descriptions can be parsed without an external end marker. A program *p* is a lossless description of a finite binary record *x* when U(p)=x. Its cost is its bit length |p|, and the prefix Kolmogorov complexity

(A1) $$K_{U}\left(x\right)=\min\left\{|p|:U\left(p\right)=x\right\}$$

is the least such cost. Changing the universal decoder changes $K_{U}\left(x\right)$ by at most an additive constant independent of *x*; this is why the paper fixes one *U* and writes K(x) [5]. A practical compressor supplies a computable upper bound on K(x) through the length of its output. It does not, in general, establish that its output is shortest.

Kolmogorov complexity and Shannon entropy answer different questions. Shannon entropy is an average uncertainty or coding cost for draws from a specified probability distribution. Kolmogorov complexity is the shortest effective description length of one finite record, whether or not a probability distribution has been supplied. This paper concerns realized histories and therefore uses *K* as its ideal description-length benchmark; no claim identifies K(x) with the output of a practical compressor or with the thermodynamic entropy of a physical substrate.

### Appendix A.2. Conditional Complexity Records What the Observer Already Knows

Conditional complexity K(x∣y) is the length of the shortest program that outputs *x* when *y* is already available as auxiliary information. The description frame *C* plays this role for the observer. It contains the fixed decoder, coding conventions, admissibility rules, and descriptive resources acquired before the event under study. Holding *C* fixed prevents a trivial reassignment of the new model-compressor to the background: its compression certificate must charge the information added beyond resources the observer already possessed. The framework is observer-relative in this explicit sense, while the theorems compare records under the same fixed frame.

Side information also separates a common mechanism from realization-specific information. If an update rule, observation map, and time horizon are shared across a family of experiments, their codes may be conditioned on rather than charged anew for every record. The remaining program must still identify the initial state, boundary data, or other realization identifier needed to reproduce the realized history. The residual-information barrier shows that this remaining cost can be extensive even when the shared mechanism is simple.

### Appendix A.3. Searching for Shorter Descriptions Has No General Stopping Rule

Candidate programs can be run in parallel. When one halts with output *x*, it certifies an upper bound on K(x); continued search may find a shorter program. No general stopping rule certifies that the shortest program has been reached or that no program below a chosen threshold will ever halt. Existence below a threshold is semidecidable, whereas nonexistence is not. This is how an optimization problem about finite records can fail to have a terminating algorithm.

### Appendix A.4. The Projection Selects the Record to Be Modeled

The map ρ turns a microscopic history into the record visible at the observer’s chosen scale. It may discard microscopic distinctions, but lossiness alone does not make a representation useful: a constant map discards every distinction and preserves no task-relevant variation. The paper therefore treats relevance as a constraint derived from the observer’s objective and measurement requirements. Once ρ is fixed, the barrier theorems ask whether its output history is compressible and whether a general procedure can construct a short description. This is the fixed-representation subproblem. The compression-certified form of algorithmic emergence evaluates the model-compressor (ρ,M) as a pair: ρ determines what is retained and omitted, while the finite parametrized model *M* compresses the retained information and transfers to new observations. When ρ is supplied, this is the fixed-representation subclass of the joint search described in Section 5.

### Appendix A.5. Terminology Guide

Several ordinary words carry technical meanings in different parts of the paper. The following guide fixes each meaning.

**Finite instance.**

One specified micro-experiment and its observation record.

**Uniform procedure/open-ended family.**

One algorithm used across inputs whose sizes have no fixed upper bound.

**Answer computation with an effective stopping rule.**

Evaluation of a declared question *q* comes with a computable certificate of termination, such as a supplied finite horizon or exact cycle detection on a finite reachable state graph.

**Open-ended answer computation.**

Evaluation of *q* has no supplied finite stopping condition and may encode an unbounded computation. This is the setting in which answer-side undecidability may arise; it is distinct from the finite-record construction barriers.

**Description frame C.**

The observer together with the coding conventions and modeling resources already available before the acquisition being studied. Costs already in *C* are not charged again.

**Objective and task.**

OF specifies what the observer seeks to minimize. Relevance states which distinctions must be retained to evaluate or pursue it. A task T specifies input/output behavior; a question *q* specifies an answer to be computed. Neither is identified with the objective’s numerical value.

**Representation/projection ρ.**

A computable map selecting which distinctions are retained under the declared relevance requirement. “Coarse-graining” is used in this informational sense and need not mean spatial averaging.

**Retained kernel $Y^{\rho }$.**

The information kept by ρ and modeled. The word “kernel” here means the retained task-relevant record, not a null space in linear algebra.

**Residual $E^{\rho }$.**

The omitted detail needed only if one wants to reconstruct the original fine-grained record exactly.

**Realization identifier ι.**

Extra information selecting one realized history from a family of histories allowed by a law plus a boundary or global prescription.

**Program/algorithm/function.**

A program is a concrete description; an algorithm is, in the convention used here, an equivalence class under a declared notion of procedural sameness; a function is input–output behavior. Following Yanofsky, these levels are related by quotient maps rather than literal subset inclusions.

**Compressive core.**

A shortest program with the model’s declared task behavior, when that behavior yields systematic coding gains. Its length is $\kappa_{T}\left(M|C\right)$.

**Model-compressor (ρ,M).**

The acquired representation–model pair. The pair is charged relative to *C*; ρ determines relevant omission, while *M* compresses the retained kernel and is reused prospectively.

**Compression across observations.**

A shared model and jointly coded parameters describe the retained sequence. Individual chunks need not be shortened, and both margins are fixed total savings rather than per-chunk requirements.

### Appendix A.6. Symbol Reference

Main-text symbols are listed first; symbols used only inside an appendix or a worked example are collected at the end. Tuple arguments of *K* use the fixed self-delimiting pairing convention specified in Section 2.

**Symbol/Term**	**Meaning**
**AIT and computability**	
*U*	Fixed universal prefix-free Turing machine.
*p*; |p|	Self-delimiting program; its length in bits. For a binary string *x*, |x| likewise denotes bit length.
K(x)	Prefix Kolmogorov complexity: length of the shortest *U*-program that outputs *x*.
K(n); $c_{A}$	Complexity of the fixed integer encoding of *n*; a method-dependent description constant. Propositions 1 and 2 use such constants for their respective codes.
K(x∣y)	Conditional prefix complexity with *y* supplied on the auxiliary input tape.
${\mathrm{Short}}_{U}\left(x,b\right)$	Relational description threshold: there exists a *U*-program *p* with |p|<b and U(p)=x. Appendix C.1 gives the corresponding foundation-neutral reading of the barrier results.
$I_{K}\left(a:b\right)$; $I_{K}\left(a:b|z\right)$	Symmetric mutual algorithmic information used here: K(a)+K(b)−K(a,b), or conditionally K(a∣z)+K(b∣z)−K(a,b∣z).
K(a,b,…)	Complexity of a tuple under the fixed computable self-delimiting pairing convention in $B_{0}$.
${\left\{0,1\right\}}^{*}$; ${\left\{0,1\right\}}^{+}$	Finite binary strings; nonempty finite binary strings.
N	{0,1,2,…}.
O(1), O(logn)	Additive coding terms independent of the varying instance; constants may depend on *U*, $B_{0}$, and fixed encodings.
*total computable*	Halts on every admissible input.
*uniform*	The same procedure must work over an admissible family of finite inputs with no fixed global upper size bound.
*semidecidable*	Positive instances are eventually recognized; the computation may fail to halt on negative instances.
*dovetailing*	Fair interleaving of candidate computations so every halting candidate is eventually observed.
*compressible*	In the raw-length sense used in Section 3.2, K(x)<|x|; |x| is a data-length benchmark, not a prefix-program length. Relative to a supplied threshold *b*, compressibility means K(x)<b.
*microlaw*	Informal term for the microscopic update rule D (sometimes together with fixed background conventions). Knowing the microlaw is distinct from knowing the realized initial state $\sigma_{0}$.
B	Computable boundary/selection prescription used in Section 3.5. In the special law-selected-IC case, $\sigma_{0}=B\left(D\right)$. Distinct from the shared description frame $B_{0}$.
$H_{D,B}$	Family of histories admitted jointly by the dynamics D and global prescription B.
$H_{\rho }$	Family of macrohistories induced by a candidate projection ρ over all initial states, Equation (33).
ι	Residual realization-identifying information sufficient to identify one realized history within $H_{D,B}$ when the family is not a singleton.
*simulation/generation*	Computing the finite record x(μ) from the complete micro-experiment μ. This is distinct from finding a shorter reusable parametrized description of the macrodata.
*macromodel construction*	Discovery of a reusable parametrized model *M* at the observational scale. With ρ supplied, only *M* remains to be found; the joint search selects (ρ,M).
*algorithmic emergence*	Acquisition of objective-relevant reusable regularity beyond an agent’s prior frame. Definition 3 specifies the compression-certified subclass used in the transfer results.
$R_{A}$; *additive description regret*	Excess output length above optimal complexity, $R_{A}\left(\mu \right)=\left|A\left(\mu \right)\right|-K\left(x\left(\mu \right)\right)\geq 0$ when *A* returns a valid standalone description; a static gap in bits, not decision-theoretic regret.
**Observer framework**	
O	Observer-agent. Conceptually finite at each finite time; the formal barrier theorems do not depend on its architecture.
$B_{0}$	Shared coding, admissibility, and pre-acquisition descriptive/modeling resources available to the observer.
$X_{t}$; $X_{\leq t}$	Conceptual substrate state and finite substrate history.
$C=(O,B_{0})$	Fixed observer/description frame, matching the notation of the persistence companion.
OF	Observer’s objective function, with its evaluation context and relevance requirements fixed before selection. It may depend on *M* and its parameters or evaluate the program more generally.
ρ	Computable observation/projection or coarse-graining map. “Coarse” is observer-relative: the map discards distinctions irrelevant to the declared task and need not be a spatial average or a thermodynamic coarse-graining.
$P_{t}^{\rho }$; $P_{\leq t}^{\rho }$	Conceptual projected observation and observation history, with $P_{t}^{\rho }=\rho \left(X_{t}\right)$.
${\mathrm{ME}}^{O}$	Modeling Engine of observer O.
$W_{t}$	Full world-model retained by the observer at time *t*.
$S_{t}^{j}$	Candidate macroscopic submodel indexed by *j*; this is the same role denoted $S_{t}^{\alpha }$ in *Pattern, Persist!*, with *j* used here to avoid collision with the structure-function budget α.
**Micro-experiments and theorem witnesses**	
$\mu =(D,\sigma_{0},\rho ,H)$	Finite micro-experiment.
Σ; *N*	Finite microstate alphabet; number of microscopic degrees of freedom.
D; $\sigma_{t}$	Computable micro-update rule; encoded microstate $\sigma_{t}=D^{t}\sigma_{0}$.
Ξ; *r*	Finite readout alphabet; for $\Xi ={\left\{0,1\right\}}^{r}$, the readout block width. Section 4.1 reuses *r* for a residual target, Section 7 for the population firing rate, and Corollary A1 for a compression witness.
*H*	Finite observation horizon of a micro-experiment; the macrohistory contains H+1 readouts.
*T*	Signed offset used only for reversible complete-state comparisons in Section 6, matching the companion notation.
$\Omega_{t}$	Sufficiently complete finite encoded state used in Section 6 for reversible complete-state bookkeeping.
$F^{T}$	Fixed computable reversible complete-state evolution over signed offset *T* in Section 6; for a reversible finite micro-experiment this role may be played by $D^{T}$.
$\rho_{\Omega }$; $Q_{t}^{\rho }$	Complete-state projection induced on the encoded state $\Omega_{t}$ in Section 6; retained complete-state kernel $Q_{t}^{\rho }=\rho_{\Omega }\left(\Omega_{t}\right)$.
$E_{t}^{\rho }$	Variable-length self-delimiting index identifying $\Omega_{t}$ inside the finite fiber of $Q_{t}^{\rho }$; no equal-fiber-size assumption is made.
$\pi_{\rho }$	Computable reversible self-delimiting representation $\Omega_{t}↔\langle Q_{t}^{\rho },E_{t}^{\rho }\rangle$.
${\tilde{C}}_{\rho }$	Local shorthand $\left(C,F,T,\rho_{\Omega }\right)$ for the fixed conditioning tuple in the conservation/localization subsection.
x(μ)	Binary encoding of the macrohistory generated by micro-experiment μ.
$D_{n}$, $\rho_{n}$	Cyclic shift rule and leading-cell readout used only for the residual-information witness, Theorem 1.
$I_{n}$, $\varrho_{n}$	Identity update and full-state readout used for the arbitrary-record embedding in Theorems 2 and 3.
$y\in {\left\{0,1\right\}}^{n}$	Arbitrary *n*-bit record. In Theorem 1 it is the initial register state with $x(\mu_{n,y})=y$; in the computational reductions $x(\nu_{y})=y$.
$z_{n}=\left(D_{n},\rho_{n},n,C\right)$	Common conditioning information used in the conditional auxiliary result of Appendix D; ${\tilde{z}}_{n}$ is its embedded counterpart in Corollary A2.
*d*	Complexity deficit allowed in Theorem 1; the lower bound is n−d.
*A*, *B*	Total computable procedures returning standalone descriptions in the discovery/optimality results and their auxiliary variants; the input domain is specified in each statement.
*b*	Supplied program-length threshold in open-ended search and Appendix D; fixed chunk width in Proposition 2 and Appendix D.6.
S(x,b); τ(x,b)	Open-ended below-threshold program search; hypothetical computable deadline excluded in Appendix D.
*s*; $c_{\mathrm{sim}}$	Promised bound on initial-state complexity and fixed simulator overhead in Section 3.5.
*g*; *J*	Explicit total parametrization of candidate initial states in Section 3.5 and the (finite, known) number of candidates. Section 7 reuses *J* for the Ising coupling.
*c*	Fixed additive tolerance in Theorem 3.
*ℓ*	Fixed compression margin in Corollaries 1 and A1: the witness satisfies K(x)<|x|−l.
$c_{F,U}$	Chaitin certification constant for formal theory *F* and machine *U*.
**Structure, regularity, and model-compressors**	
S	Finite-set model containing record *x* in algorithmic statistics.
α	Budget on model complexity K(S).
$h_{x}\left(\alpha \right)$	Kolmogorov structure function: minimum residual index length at model-complexity budget α, with value +∞ if no finite-set model is feasible.
$\lambda_{x}\left(\alpha \right)$	Constrained two-part/MDL length K(S)+log|S|.
$G_{x}\left(\alpha_{1},\alpha_{2}\right)$	Net structure gain over a budget interval, Equation (25); a crack is an interval on which it is positive beyond the O(logn) coding uncertainty.
$M=(e_{M},g_{M})$	Shared finite model: $e_{M}$ jointly encodes retained observations through realization coordinates and $g_{M}$ regenerates them under the declared interface.
(ρ,M); *model-compressor*	Factorization separating the choice of what is modeled from the reusable model itself: ρ defines the retained/residual split and *M* organizes and encodes the retained kernel. Figure 1 calls the pair a representation–model pair. In scientific applications, this is a scientific model/compressor; the formal object is agent-generic.
T; $F_{M}^{T}$; $\equiv_{T}$	Declared task; task-relevant behavior induced by *M*; equivalence of implementations that realize the same behavior on the task domain.
$\kappa_{T}\left(M|C\right)$	Task-relative functional core complexity: length of a shortest program, relative to the declared task and pre-acquisition frame, that reproduces *M*’s task-relevant behavior.
*compressive core*	A shortest representative of a model’s task-equivalence class when its behavior yields systematic coding gains.
*D*; $Y^{\rho }$; $E^{\rho }$	Fine-grained finite record; retained algorithmic kernel $Y^{\rho }=\rho \left(D\right)$; residual index completing the lossy projection so $D↔(Y^{\rho },E^{\rho })$.
X	Fixed finite computable record domain used when interpreting the projection fiber $S_{\rho ,Y}$ as a finite-set model.
$R_{C}$; *q*	Admissible projection class under frame *C*; a fixed macroscopic question in the answer-side results, distinct from the objective OF.
*w*; A(w) (Problem 1)	Finite code for a construction instance and the valid representation–model pair returned by one total computable constructor on that family. The code declares the construction inputs, frame, objective and relevance requirements, admissible classes, baselines, margins, and evaluation protocol.
${\left|\langle \rho ,M\rangle \right|}_{C}$	Incremental self-delimiting code needed to add the pair (ρ,M) beyond the pre-acquisition frame *C*. If ρ is supplied, its incremental cost is zero and the ledger describes the fixed-representation subclass.
${\left|M\right|}_{C}$; |M|	Incremental code cost of *M* alone relative to *C*; with ρ fixed, |M| abbreviates ${\left|M\right|}_{\left(\rho ,C\right)}$. These are actual code lengths, unlike the minima *K* and $\kappa_{T}$.
$L_{\mathrm{keep}}$; $L_{\mathrm{full}}$	Retained lossy model-code length for (ρ,M,θ) relative to *C*; corresponding lossless AIT ledger after adding the residual $E^{\rho }$. Either is a compression only when shorter than its declared baseline.
$L_{M}^{\rho }\left(Y^{\rho }|u,C\right)$; $R_{\rho }\left(M;Y^{\rho },u\right)$	Explicit two-part length $\left|M|+\right|e_{M}\left(Y^{\rho },u\right)|$ for a held model at fixed ρ; its unknown excess over $K(Y^{\rho }|\rho ,u,C)$.
$\theta_{i}$; $u_{i}$	Realization coordinates or states; conditions supplied to both encoder and decoder. Information inferred from the observations is coded or recovered from already decoded data.
$D_{m}$; $Y_{m}^{\rho }$; $\theta_{m}$	First *m* observation chunks, their retained sequence, and its jointly represented realization coordinates.
$L_{M}\left(\theta_{m}|u_{m},C\right)$	Actual joint parameter-code length under the acquired model, including framing and corrections needed for exact retained-data reconstruction.
$L_{0}$; $B_{\rho }$	Predeclared valid code lengths for the original and retained observation sequences, under their fixed encodings and supplied conditions. Separate per-chunk sums are allowed as special cases.
$h_{m}$	Shared history or model state available from the construction observations for prospective coding; additional state is charged.
$\delta_{\mathrm{comp}}$, $\delta_{\mathrm{test}}$	Fixed positive total margins for net compression and prospective reuse; independent of the number of chunks.
$a_{\mathrm{bridge}}$; Φ	Bridge information not already available from the conditioned microlaw/frame; fixed restricted-class construction map $\left(\rho ,M\right)=\Phi \left(D,a_{\mathrm{bridge}}\right)$ in Section 7.
**Appendix-local and example-local symbols**	
$\kappa_{T,\varepsilon }\left(M|C\right)$; $d_{T}$	Approximate core benchmark at tolerance ε under a predeclared discrepancy $d_{T}$, Equation (A8).
$P_{0}$; $L_{0}\left(z\right)$; *Q*; $L_{Q}$	Declared baseline distribution and its ideal codelength; competing (semi)measure and its codelength, Proposition A1.
$Q_{m}$; L(m); $L_{\mathrm{sel}}$	Countable candidate codes, prefix code length of the index *m*, and the two-part selected codelength in Appendix B.
$c_{\mathrm{acq}}$; $B_{N}$; $J_{N}$; $G_{N}$	Fixed acquisition cost, baseline and joint parameter-code lengths, and net gain after *N* observations, Equation (A9); $g_{i}$ is an incremental saving when using sequential codes.
$L_{\mathrm{res}}$	Actual joint residual-code length needed to restore the original observations, Equation (A3).
$Q_{D_{m}}$	Test code fixed by the frozen pair after the construction records $D_{m}$, Equation (A15).
$T_{U}\left(b\right)$	Maximum halting runtime of *U*-programs of at most *b* bits, under fixed step-counting semantics.
${\mathrm{Desc}}_{U}$; ${\mathrm{NoShort}}_{U}$	Description relation and the negation of ${\mathrm{Short}}_{U}$ in Appendix C.1.
*r*; *p* (Corollary A1)	Explicit compression witness and explicit competitor that beats the constructor’s output.
*F*; f:S→S	Computably axiomatized formal theory in Appendix C.3; finite-state transition map in Appendix C.4. Both are distinct from the reversible evolution $F^{T}$.
U; $x_{U}$; *e*	Larger effectively encoded system class, its finite record map, and the computable record-preserving embedding in Corollary A2.
F; $\rho_{*}$; $q_{*}$; $a_{*}$; Y; $A_{*}$; $E_{*}$	Fixed family of microscopic specifications, fixed projection and question, declared answer map and answer set, and the constructor–evaluator pair in Appendix E.
$v_{m}$; $q_{A}\left(v;m\right)$; $M_{A}$; $L^{*}\left(v_{m}\right)$	First *m*-chunk block left unshortened by *A*; complete returned code; reusable search-and-replay model; best qualifying complete length in Appendix D.6.
*J*, *h*, *m*, β; $V_{j}$, *r*, *v*	Ising coupling, field, magnetization, and inverse temperature; neuron membrane potentials, population rate, and mean potential. Local to the examples of Section 7.

## Appendix B. From Reusable Regularity to Compression and Prediction

Repeated use can turn a model’s coding advantage into a net saving after its fixed cost is paid. This appendix gives the full code accounting, the functional-complexity benchmark, the amortization condition, and baseline-relative evidence from construction and prospective reuse.

### Appendix B.1. Joint Retained Codes and the Full Record

The split used in Figure 2 separates the record from what the observer retains:

(A2) $$D\;⟷\;\left(Y^{\rho },E^{\rho }\right),\;Y^{\rho }=\rho \left(D\right).$$

The representation keeps $Y^{\rho }$ and omits $E^{\rho }$. For a sequence, a shared model and its joint parameter code reconstruct $Y_{m}^{\rho }$ exactly. An additional joint residual code restores the original observations. Write $L_{\mathrm{res}}\left(E_{m}^{\rho }|Y_{m}^{\rho },\rho ,u_{m},C\right)$ for its actual length, including framing, so that

(A3) $$\begin{array}{cc} L_{\mathrm{full}}\left(D_{m};\rho ,M|u_{m},C\right) & =L_{\mathrm{keep}}\left(D_{m};\rho ,M|u_{m},C\right)\\ & \;+L_{\mathrm{res}}\left(E_{m}^{\rho }|Y_{m}^{\rho },\rho ,u_{m},C\right)+O\left(1\right). \end{array}$$

A sum $\sum_{i}\left|E_{i}^{\rho }\right|$ of separate residual-code lengths is a special case. The residual code and its interpretation must be specified by the charged representation or prior frame. Thus the usual MDL phrase “data given the model” can contain both the realization coordinates the model retains and the detail the observer omits. This is the operational counterpart of the structure-function accounting.

With common arguments suppressed, the baseline difference separates as

(A4) $$L_{0}-L_{\mathrm{keep}}=\left(L_{0}-B_{\rho }\right)+\left(B_{\rho }-L_{\mathrm{keep}}\right).$$

The first term is the saving due to the chosen representation under the declared baselines; the second is the net compression required by Definition 3(iii). Neither is an invariant measure of omitted information. Objective-based relevance justifies omission; the second comparison asks whether a model also captures regularity in what remains.

For a fixed acquired model, the sequence encoder can code parameter values jointly or sequentially. Both conventions permit dependence across observations. All descriptions remain decodable given the same declared conditions. A representation, initial state, parameter value, or observation learned from the data cannot be inserted into those conditions for free.

For a held model, the observer knows the length of its code without knowing how close that code is to optimal. Fix ρ, supplied conditions *u*, and one retained kernel $Y^{\rho }$. Define the explicit two-part length

(A5) $$L_{M}^{\rho }\left(Y^{\rho }|u,C\right):=\left|M\right|+\left|e_{M}\left(Y^{\rho },u\right)\right|.$$

Here |M| abbreviates the acquired model-code cost ${\left|M\right|}_{\left(\rho ,C\right)}$ with ρ fixed. The associated held-model regret is

(A6) $$R_{\rho }\left(M;Y^{\rho },u\right)=L_{M}^{\rho }\left(Y^{\rho }|u,C\right)-K\left(Y^{\rho }|\rho ,u,C\right).$$

The first quantity is computable from the held code; the second is not computable, in general, because its benchmark is the shortest description. A newly found shorter model witnesses suboptimality, whereas failure to find one does not certify near-optimality. Theorem 3 gives the underlying record-level obstruction, and Proposition 2(b) gives the corresponding failure for complete sequence codes among qualifying model-compressors.

### Appendix B.2. Complete Coding Accounts for the Glider and Cellular Automaton

The examples in Section 5.3 compare complete codes for the same retained observations. In each comparison, the pre-acquisition frame *C* fixes the objective, readout, dimensions, boundary conditions, horizon, and decoding conventions; it supplies no new initial state to either decoder. Both margins are fixed at 128 bits. A test block starts a separate run after the acquired model is fixed. The accompanying example code contains the codecs, verifiers, and numerical results.

*Glider: finite experiment and shared information.* The microstate is a periodic binary grid *G* together with a cyclic register *s*:

$$\sigma =\left(G,s\right)\in {\left\{0,1\right\}}^{256\times 256}\times {\left\{0,1\right\}}^{4096}.$$

The grid follows Conway’s Game of Life [16]: a live cell survives with two or three live neighbors among its eight neighbors, and a dead cell becomes live with exactly three. All cells update simultaneously. The register shifts cyclically by one cell, independently of the grid. Writing *F* for the Life update, the retained record and horizon are

$$H=31,\;x\left(\mu \right)=\left(G_{0},G_{1},\ldots ,G_{31}\right),\;G_{t}=F^{t}\left(G_{0}\right).$$

The objective requires the grid movie and none of the register. Both competing codes reconstruct that movie, so omitting the register contributes none of the compared savings.

The shared frame supplies Python 3 (version 3.8 or later) execution, a generic source-module interface, Life, and the geometry. The entire acquired source, including ρ, the glider template, its phases and directions, encoder, decoder, and fallback, contains 2366 bytes. An Elias gamma code for 2367 supplies a 23-bit length header, giving 8·2366+23=18,951 bits. This unminimized source includes Life code already available in *C*; charging that copy is conservative. Its byte length is an implementation cost, not an estimate of *K*.

A matching movie costs one mode bit, two 8-bit position coordinates, two phase bits, and two direction bits: 21 bits. Their roles remain fixed across runs. The encoder checks the observed movie; forecasting from those coordinates additionally requires them before the forecast. The code is lossless on all nonempty finite binary torus movies of side length at least eight: it searches the finitely many glider configurations and sends literal frames after an escape flag if none matches. Geometry and movie length are shared.

*Glider: acquisition and reuse against a sparse incumbent.* The incumbent encodes an initial grid by a mode bit, a gamma-coded live-cell count plus one, and the live-cell positions, then simulates Life. For five live cells, this costs 1+5+5·16=86 bits. A movie inconsistent with Life uses a literal-frame escape instead. The acquired model, therefore, saves 86−21=65 bits per glider run before acquisition. The protocol applies both codes separately to each run, with run counts and boundaries shared. Table A1 gives a passing construction and subsequent test.

**Table A1:** Glider accounting in bits against the sparse initial-grid simulator. Construction uses 294 separately initialized glider runs; testing uses two further runs after acquisition.

Quantity	Construction	Test
Acquired representation and model	18,951	0
Coordinates and mode flags	6174	42
Correction data	0	0
Complete retained code	25,125	42
Sparse initial-state simulation baseline	25,284	172
Net saving	159	130
Required saving	128	128

A single movie costs 18,951+21=18,972 bits and fails against the 86-bit incumbent. At 292 runs, the net saving is 29 bits; 294 runs are needed to reach the construction margin. Two later runs save 130 bits without paying acquisition again. A continuing run with its last grid already shared is a different protocol: a simulation match flag can cost one bit, so the 21-bit coordinate code provides no positive saving.

For comparison, an incumbent without the sparse-grid code stores the 65,536 initial cell values and a mode flag, costing 65,537 bits per run. The model then saves 46,565 bits on the first movie and 65,516 on a later run; a literal 32-frame movie costs 2,097,152 bits. These are alternative declared baselines, not additional gains in Table A1. Restoring the omitted register separately costs its 4096-bit initial state, giving a complete first-record description of 18,972+4096=23,068 bits. No sampled register seed is certified incompressible. Ordinary Life need not be reversible; this example concerns relevance and compression. The verifier checks 240 configurations against independent Life evolution, including boundary crossings, escape cases, and the minimum run counts above.

*Cellular automaton: objective, domain, and decoder.* The microscopic states are 360-bit rows on a periodic ring, evolving synchronously by rule 146. A cell becomes 1 for the neighborhoods 001, 100, and 111 and becomes 0 for all others; each neighborhood lists the left neighbor, the cell itself, and the right neighbor. The readout applies a fixed Boolean table to each aligned triple, producing 120 bits every three microscopic steps. The initial row and forty coarse updates give 41 rows and 4920 retained bits. The AND task requires the fully occupied triples; the OR task requires the nonempty triples. Each objective and readout is fixed before its model search.

For each task, *C* supplies rule 146, the readout, alignment, dimensions, timing, and a generic interpreter for elementary rules and the codes below. The incumbent sends one mode bit and the 360-bit microscopic initial row, then simulates and projects: 361 bits. Its literal escape covers records inconsistent with the microscopic law. The original microscopic movie has the same 361-bit simulation code, satisfying the baseline restriction in Definition 3. The blocky initial rows used for the figure and test are generated reproducibly by seeds 7 and 23 in the verifier; those seeds and their generating recipe are not supplied to either decoder.

The acquired module contains an eight-bit projection table and an eight-bit coarse-rule table, each preceded by a seven-bit Elias gamma header for length eight: 30 bits in total. The module stores its own copy of the task’s supplied readout; counting that redundant copy is conservative. The interpreter and coordinate interface are preinstalled, so this is a table cost, unlike the glider’s source-module cost. These costs concern different declared prior frames and are not estimates of *K*.

The coordinate code contains a one-bit flag and the initial 120-bit coarse row. With flag zero, the rule generates the movie exactly. With flag one, a correction list follows: for *k* differing bits in the autonomously generated movie, encode k+1 by an Elias gamma code and then list the *k* positions in increasing order, using 13 bits per position among the 4920 entries. The decoder flips those bits. This fixes a lossless code for every binary coarse movie of the declared dimensions, including movies for which the selected rule is inaccurate.

*Cellular automaton: successful construction and a failed search.* For each projection, the search minimizes one-step bit error over the 256 elementary rules, breaking ties by rule number. AND selects rule 128 with no errors. Exhaustive checking of all 512 nine-bit microscopic light cones verifies exact closure for this projection and timing. The model, therefore, needs no correction data on either the construction or fresh test run. Table A2 records the savings beyond the known microscopic simulator.

**Table A2:** Cellular-automaton accounting in bits for the declared table interpreter. The model is fixed before the separately initialized test run. The OR column uses rule 182 with the specified correction list; its negative savings are failures of this code.

Quantity	AND, Rule 128	OR, Rule 182
Acquired projection and rule tables	30	30
Initial coarse row and flag	121	121
Construction correction data	0	26,307
Complete construction code	151	26,458
Microscopic simulation baseline	361	361
Construction saving	210	−26,097
Test acquisition cost	0	0
Test initial coarse row and flag	121	121
Test correction data	0	27,440
Complete test code	121	27,561
Test microscopic baseline	361	361
Test saving	240	−27,200
Required margin on each block	128	128

OR selects rule 182, with 1370 one-step errors among 4800 observed transitions. Running that rule autonomously from the initial coarse row instead produces 2022 differing bits across the construction movie, including 54 of 120 bits in its final row. Their correction list costs 21+13·2022=26,307 bits. The held rule produces 2109 differences on the test movie, costing 23+13·2109=27,440 correction bits. Both codes reconstruct exactly after correction, but neither beats the microscopic baseline.

For the caption’s closure count, traverse the observed transitions in time and cell order, store the first successor for each coarse neighborhood, and count subsequent disagreements: zero for AND and 1476 for OR. This count differs from the minimum one-step error because its reference is the first successor rather than the majority successor. Any such conflict rules out an exact elementary update on all those transitions. Its magnitude depends on the counting convention. The finite search and failed certificate concern the stated rule class and code; they leave other model classes and coding methods open. The verifier checks every local light cone, all 256 rules, exact reconstruction with and without corrections, and both coding accounts.

### Appendix B.3. Functional Complexity and Implementation Cost

The acquired implementation *M* realizes its declared task behavior. A fixed interpreter can therefore reproduce that behavior from the incremental description of *M*, giving

(A7) $$\kappa_{T}\left(M|C\right)\leq {\left|M\right|}_{C}+O\left(1\right),$$

where ${\left|M\right|}_{C}$ is the actual incremental description length relative to the prior frame. The excess can include unused code or precomputed information that improves execution speed. The bound concerns description length; it gives no runtime comparison.

For a predeclared nonnegative discrepancy $d_{T}$ between behaviors (for example, the largest output disagreement over the domain), vanishing on identical behaviors, and a tolerance ε≥0, let $F_{p,C}^{T}\left(x\right):=U\left(p,\langle x,T,C\rangle \right)$ on programs that halt on every admissible input. The approximate benchmark is

(A8) $$\kappa_{T,\varepsilon }\left(M|C\right):=\min_{p}\left\{|p|:d_{T}\left(F_{p,C}^{T},F_{M}^{T}\right)\leq \varepsilon \right\},$$

with the domain, discrepancy, tolerance, and supplied information fixed before comparison. This minimizes length over admissible approximations to the given behavior. Closeness within a tolerance need not be transitive, so it does not generally define an equivalence class.

### Appendix B.4. Regularity Can Precede Net Compression

Let the acquired pair have fixed incremental cost $c_{\mathrm{acq}}$. Suppressing common conditions, write $B_{N}$ for the baseline length of the first *N* observations and $J_{N}$ for their joint parameter-code length under the same model. The net gain is

(A9) $$G_{N}=B_{N}-J_{N}-c_{\mathrm{acq}}.$$

For a sequential implementation, define $g_{i}$ as the difference between the baseline and model’s additional code lengths on use *i*, including any updated state or framing. Then $B_{N}-J_{N}=\sum_{i=1}^{N}g_{i}$, up to a fixed initialization cost already included in $c_{\mathrm{acq}}$. Separate coding of each observation is one instance of this accounting; dependence across the parameter sequence is allowed.

If ${\mathrm{lim inf}}_{N\to \infty }\left(B_{N}-J_{N}\right)/N=\bar{g}>0$, the gain eventually exceeds any fixed $\delta_{\mathrm{comp}}$. For constant additional saving g>0 per use, condition (iii) holds once $Ng\geq c_{\mathrm{acq}}+\delta_{\mathrm{comp}}$. This is a sufficient condition for eventual amortization, not a requirement that every use save bits. A large implementation, including one that stores a recurring image literally, can, therefore, become compressive through reuse.

The cost is that of the acquired implementation even if a shorter equivalent program exists. Failure to repay it on a finite batch does not show that no useful regularity has been captured. Without a usable lower bound on the accumulated savings, this observation supplies no deadline. Proposition 2 concerns failure on the available finite batch even when another pair already meets the certificate there; it does not rule out later amortization by the original method.

### Appendix B.5. Compression Relative to a Declared Baseline

Let $P_{0}$ be a probability mass function on a finite or countable set of possible records *z*, and write

(A10) $$L_{0}\left(z\right):=-\log_{2}P_{0}\left(z\right)$$

for its ideal codelength; a Kraft-valid main-text baseline $L_{0}\left(D\right)$ is of this form up to normalization, as discussed below. Let *Q* be any competing probability mass function or semimeasure, $\sum_{z}Q\left(z\right)\leq 1$, with codelength $L_{Q}\left(z\right):=-\log_{2}Q\left(z\right)$. A semimeasure is enough because Kraft’s inequality associates every prefix code with weights whose total mass is at most one.

**Proposition A1** (No hypercompression relative to a baseline)**.**

*Let Z be a record drawn from $P_{0}$. For every k≥0,*

(A11)
$$P_{0}\left(L_{0}\left(Z\right)-L_{Q}\left(Z\right)\geq k\right)\leq 2^{-k}.$$

**Proof.** Let $A_{k}=\left\{z:L_{0}\left(z\right)-L_{Q}\left(z\right)\geq k\right\}$. For every $z\in A_{k}$, $Q\left(z\right)\geq 2^{k}P_{0}\left(z\right)$. Hence

(A12) $$P_{0}\left(A_{k}\right)\leq 2^{-k}\sum_{z\in A_{k}}Q\left(z\right)\leq 2^{-k}.$$

   □

Thus, if the baseline $P_{0}$ generated the data, a *k*-bit advantage by another valid code, fixed in advance or with its selection cost charged as below, is an event of baseline probability at most $2^{-k}$. This is the no-hypercompression inequality used in MDL theory [4,85]. For orientation, a 20-bit gain has a baseline probability of at most about $10^{-6}$. The statement is relative to $P_{0}$: it is evidence against that baseline, not a probability that the discovered model is metaphysically true, nor a distribution-free theorem that the future must resemble the past.

A Kraft-valid baseline code can likewise be associated with subprobability weights by Kraft’s inequality; the same algebra then bounds those weights. A literal probability statement requires a declared normalized data-generating distribution such as $P_{0}$ above. In either case, the argument does not apply to an arbitrary numerical “baseline” that violates Kraft’s inequality. This qualification matters when description length is used operationally rather than probabilistically.

### Appendix B.6. Model Selection Adds a Description Cost

Suppose a search ranges over a countable family of candidate codes $Q_{m}$, and the index *m* is itself described by a prefix code of length L(m) with $\sum_{m}2^{-L(m)}\leq 1$. After seeing *z*, the search may choose whichever candidate looks best. The resulting two-part selected code length is

(A13) $$L_{\mathrm{sel}}\left(z\right)=L\left(m\left(z\right)\right)+L_{Q_{m(z)}}\left(z\right).$$

It is still a valid code because

(A14) $$\sum_{z}2^{-L_{\mathrm{sel}}\left(z\right)}\leq \sum_{m}2^{-L(m)}\sum_{z}Q_{m}\left(z\right)\leq 1.$$

Consequently Proposition A1 continues to hold for data-dependent model selection *only when the information needed to identify the selected model is charged*. If that cost is omitted, one can manufacture apparent regularity by searching a sufficiently rich family and reporting only the winning fit.

This is the coding-theoretic reason for keeping ${\left|\langle \rho ,M\rangle \right|}_{C}$ visible in Definition 3. Information discovered in the representation ρ is no more free than information discovered in the model *M*. If ρ was already supplied or inherited in the pre-acquisition frame *C*, its incremental cost is zero; if it is learned during construction, its code must be paid. The retained-kernel baseline $B_{\rho }$ then asks a separate question: after relevance-guided omission has fixed what is to be modeled, does *M* compress the information that remains?

### Appendix B.7. Prospective Reuse and the Prediction–Coding Link

Compression of construction records alone does not provide a measure-free bound on future success. An arbitrary continuation can always violate a pattern inferred from a finite prefix. The paper therefore freezes (ρ,M) and its coding rules before evaluation on a new block. Its state may evolve according to those rules. The common state $h_{m}$ is recoverable by the decoder from the charged construction code and declared conditions, or is separately charged. The incumbent baseline has access to the same shared history; it need not use that information in the same way. If an incumbent already exploits a repetition, rediscovering it need not improve that baseline. The literal baseline used in Appendix D.6 ignores prior observations by design and is fixed throughout that family.

The no-hypercompression statement has an immediate conditional form. Let $D_{m}$ denote the construction records, and let the frozen pair determine a test code $Q_{D_{m}}$ before a held-out observation block *Z* is observed. For any declared conditional baseline $P_{0}\left(\cdot |D_{m}\right)$,

(A15) $$P_{0}\left(L_{0}\left(Z|D_{m}\right)-L_{Q_{D_{m}}}\left(Z\right)\geq k|D_{m}\right)\leq 2^{-k}.$$

Conditional on the construction history, a large held-out compression gain is, therefore, again unlikely under the baseline. Under the declared sequential evaluation and baseline, repeated prospective success is stronger evidence that the discovered regularity is reusable rather than a one-record accident.

For sequential probabilistic models under logarithmic loss, the coding–prediction connection is exact at the level of ideal codelengths. Let $z^{i}=\left(z_{1},\ldots ,z_{i}\right)$ denote the observed prefix, and let $q_{i}\left(z_{i}|z^{i-1}\right)$ be the probability assigned to observation $z_{i}$ given its past. The joint probability $Q\left(z^{n}\right)=\prod_{i=1}^{n}q_{i}\left(z_{i}|z^{i-1}\right)$ satisfies

(A16) $$-\log_{2}Q\left(z^{n}\right)=\sum_{i=1}^{n}-\log_{2}q_{i}\left(z_{i}|z^{i-1}\right),$$

so predictive code length is cumulative next-observation log loss  [85]. Such sequential coding records how much probability the model assigned to observations as they arrived.

Log loss is convenient, not uniquely privileged for converting prediction into compression. Consider a deterministic sequential predictor of a binary sequence $z_{1},\ldots ,z_{n}$, with the predictor and all of its supplied inputs known to the encoder and decoder. If it makes *k* mistakes, the encoder can transmit the set of mistaken positions; the decoder reruns the predictor and flips its output at those positions. Given *n* and *k*, the error pattern requires

(A17) log2nk

bits, plus self-delimiting framing for *n* and *k* when not supplied, and any charged model or input information. Thus ordinary binary prediction already yields a lossless code whenever its errors are sufficiently sparse. More general discrete predictors admit analogous error descriptions, with any incorrect symbol values also encoded.

Predictive success can, therefore, provide compressive capability even in a large model. To transfer a particular compression barrier to a predictor-discovery claim, the predictor-to-code conversion must be made explicit and the theorem’s information, cost, validity, and guarantee assumptions preserved. For log-loss predictors the conversion is immediate; for ordinary discrete prediction it can be as elementary as the mistake code above.

### Appendix B.8. Relation to Solomonoff Induction

Solomonoff induction supplies an idealized predictive justification for simpler generators. Its universal predictor weights a program *p* by $2^{-|p|}$ and multiplicatively dominates every lower-semicomputable source up to a constant controlled by the source’s description complexity. For a computable data-generating source, the universal predictor’s expected cumulative excess logarithmic loss relative to that source is bounded by the source’s description complexity, up to fixed coding constants  [8,9,10]. This concerns effective description length, rather than raw parameter count. It gives a predictive bound within the stated source class; it does not imply that the physical world is sampled from the universal semimeasure or is itself simple.

One possible world-side motivation, explored in earlier KT work, is that random-program generation strongly favors low-complexity outputs [11]. A broader result is that many low-complexity computable input–output maps exhibit an approximately exponential bias toward simple outputs even when their inputs are sampled uniformly  [12]. These considerations motivate a simplicity-biased inductive stance; they are logically separate from Proposition A1.

Compression after model costs are paid provides evidence against a declared baseline; prospective gains test reuse beyond the selecting data. These guarantees concern the model’s achieved coding performance. They supply neither a shortest equivalent implementation nor a universal method for acquiring a qualifying model.

## Appendix C. Description Relations, Uncomputability, and Certification

Numerical Kolmogorov complexity packages three distinct questions: whether a short description exists, whether its least length can be computed, and whether a lower bound can be certified. This appendix separates them and then compares finite description problems with physical systems that have unbounded resources.

### Appendix C.1. A Relational Reading of Kolmogorov Complexity

Standard AIT writes the shortest description length as the numerical function

(A18) K(x)=min{|p|:U(p)=x}.

That notation is convenient, but it combines two logically separate ingredients: the underlying description relation and the step of packaging its length as a numerical value. For the first ingredient, define

(A19) $${\mathrm{Desc}}_{U}\left(p,x\right):⟺U\left(p\right)=x,\;{\mathrm{Short}}_{U}\left(x,b\right):⟺\exists p\left[{\mathrm{Desc}}_{U}\left(p,x\right)\wedge \left|p\right|<b\right].$$

The complementary statement

(A20) $${\mathrm{NoShort}}_{U}\left(x,b\right):⟺\neg {\mathrm{Short}}_{U}\left(x,b\right)$$

says that no description below the threshold exists. In standard classical AIT,

(A21) $$K\left(x\right)<b⟺{\mathrm{Short}}_{U}\left(x,b\right),\;K\left(x\right)\geq b⟺{\mathrm{NoShort}}_{U}\left(x,b\right).$$

Thus many statements normally written with *K* can instead be stated directly in terms of descriptions and thresholds.

In constructive type theory, Forster, Kunze, and Lauermann formalize Kolmogorov complexity as a *functional relation* rather than as a total function. They prove classical totality only under double negation and emphasize that a constructive proof of ordinary totality would, under Curry–Howard, amount to a computable function for Kolmogorov complexity. They also show that, in their setting, the unrestricted principle assigning least elements to all inhabited predicates entails the law of excluded middle, and they choose the direct short-description predicate when formalizing compressibility  [71]. A complementary classical analysis appears in reverse mathematics.

For plain complexity *C* (not the frame *C* of the main text), Nies and Shafer work in ${\mathrm{RCA}}_{0}$ with the threshold relation “C(σ)≤n” defined by the existence of a description. Their base theory proves that each individual string has a least description length using the $\Sigma_{1}^{0}$ least-element principle, while it does not prove that the noncomputable map σ↦C(σ) exists as a second-order function object [86]. In HOL4, Catt and Norrish take the more classical route and define Kolmogorov complexity as a function; Forster et al. note that this packaging requires classical logic together with a unique choice operator [71,87].

These examples are not criticisms of standard AIT. They show that the description relation makes fewer foundational commitments, while the familiar numerical *K* packages the same information in a form convenient for classical mathematics.

The three barriers in the main text can be read at this relational level. The counting proof of Theorem 1 bounds how many records can possess a short conditional description; its lower bound K(x∣z)≥b can be read as the absence of any conditional program shorter than *b*. The discovery antecedent K(x)<|x| in Theorem 2 is exactly ${\mathrm{Short}}_{U}\left(x,|x|\right)$. The optimality barrier also has a witness-based form that avoids referring to an exact optimum:

**Corollary A1** (Relational optimality barrier)**.**

*Let A be a total computable procedure that returns a valid description U(A(μ))=x(μ) for every finite micro-experiment μ. For every c,l∈N there are a finite micro-experiment μ and programs r,p such that*

(A22)
U(r)=x(μ), |r|<|x(μ)|−l, U(p)=x(μ), |A(μ)|>|p|+c.

*Thus the record has a compression witness r, and another valid description p beats the output of A by more than c bits. Both exist for each such instance; no uniform procedure is promised to find them.*

**Proof.** Use the arbitrary-record embedding $\nu_{y}$ of Equation (7) and $\mathrm{pad}\left(z\right)=z0^{\left|z\right|}$. Define

(A23) $$f\left(z\right):=|A\left(\nu_{\mathrm{pad}(z)}\right)|.$$

Because *A* is valid and pad is injective, distinct *z* require distinct returned programs. Only finitely many programs have length at most any fixed *k*, so the total computable function *f* is unbounded. Given *k*, search strings in length-lexicographic order for the first $z_{k}$ with $f(z_{k})>k$. The search terminates. A fixed program with a self-delimiting encoding of *k* can repeat this search and output $\mathrm{pad}(z_{k})$, giving a valid competitor $p_{k}$ of length O(logk). As *k* grows, $|z_{k}|$ leaves every finite bound, since each fixed *z* has a fixed value f(z) and is excluded once k≥f(z). Hence for sufficiently large *k*, the explicit program that contains $z_{k}$ self-delimitingly and appends $|z_{k}|$ zeroes has length

(A24) $$|z_{k}\left|+2\log\right|z_{k}\left|+O\left(1\right)<2\right|z_{k}|-l=|\mathrm{pad}\left(z_{k}\right)|-l;$$

call this compression witness $r_{k}$. Choosing *k* large enough also gives $k>|p_{k}|+c$. Then

(A25) $$|A\left(\nu_{\mathrm{pad}(z_{k})}\right)|=f\left(z_{k}\right)>k>\left|p_{k}\right|+c,$$

which proves the claim with $\mu =\nu_{\mathrm{pad}(z_{k})}$.    □

In classical AIT, Corollary A1 immediately implies the numerical regret statement because K(x)≤|p| for every valid description *p*. Conversely, if a shortest description is available as a mathematical object, Theorem 3 supplies such a competitor by choosing a shortest program. The two presentations, therefore, have the same classical content, while the relational form makes the observer-side witness structure explicit. The paper uses *K* as the conventional compact benchmark, not as something the observer is assumed to compute or possess.

The distinction also controls the possible ontological reading of the barriers. Classical AIT may treat K(x) and the truth of ${\mathrm{NoShort}}_{U}\left(x,b\right)$ as determinate even when no algorithm computes the former and no fixed effective theory certifies the latter without bound. The obstacle then concerns an observer’s access to a classically given object. For a finite micro-experiment, direct simulation still generates the finite record and answers computable questions about it. Failure to find a compressed macroscopic theory, therefore, does not imply that the corresponding individual answer is absent or not entailed by the microscopic specification.

An unbounded problem with a noncomputable answer map presents a stronger effective obstruction: the answer to the fixed macroscopic question cannot be returned uniformly over the family. In a constructive setting, where an existential assertion carries a demand for a witness, either failure can constrain which objects or properties are available within the theory; packaging the description relation as a total numerical function itself requires additional principles. Their ontological significance, therefore, depends on which constructive requirements the framework attaches to existence. Neither establishes that higher-level facts fail to follow from complete lower-level facts, as Chalmers’s strong emergence requires [65,71]. Section 8.2 compares these tasks with Chalmers’s question.

### Appendix C.2. Classical Numerical Complexity Is Uncomputable

The discovery and optimality reductions depend on a common classical obstruction: shortest-program length is mathematically defined but cannot be returned by one terminating algorithm on all strings. Fix a universal prefix-free machine *U* and write K(x)=min{|p|:U(p)=x} in the usual numerical notation.

**Theorem A1** (Kolmogorov, Solomonoff, Chaitin)**.**

*There is no total computable function that, on every input string x, outputs K(x).*

**Proof.** Suppose *f* is total computable with f(x)=K(x) for all *x*. Consider a program *Q* that, on input *k*, enumerates ${\left\{0,1\right\}}^{*}$ in length-lexicographic order and returns the first *y* with f(y)>k. Such a *y* exists, because among the $2^{n}$ strings of length *n* at most $2^{n}-1$ have descriptions shorter than *n* bits, so some string of each length n>k has K≥n>k. The program halts, and its total description length is |Q|+O(logk) once *k* is supplied self-delimitingly. Its output $y_{k}$, therefore, satisfies $K\left(y_{k}\right)\leq \left|Q\right|+O\left(\log k\right)$, while the search guarantees $K\left(y_{k}\right)=f\left(y_{k}\right)>k$. For large *k* these are incompatible, so *f* does not exist.    □

**Theorem A2** (Exact partial computation has finite domain)**.**

*If $f:{\left\{0,1\right\}}^{*}⇀N$ is partial computable and f(x)=K(x) whenever f(x) is defined, then dom(f) is finite.*

**Proof.** Suppose the domain were infinite. Complexity is unbounded on any infinite set, since only finitely many strings have complexity at most a given bound. Given *k*, dovetail *f* on all strings and output the first $x_{k}$ whose returned value exceeds *k*. This search terminates, so $K\left(x_{k}\right)\leq K\left(k\right)+c_{f}$ for a constant depending only on the fixed search and machine. Correctness gives $K\left(x_{k}\right)=f\left(x_{k}\right)>k$, contradicting K(k)=O(logk) for sufficiently large *k*.    □

This is the classical partial-computation obstruction stated for plain complexity in ([26] Theorem 1); the proof above gives the prefix version used here. A partial method returning a shortest program whenever it answers would yield such an *f* by taking program length. An always-valid compressor can still happen to be optimal on infinitely many inputs if it also answers suboptimally elsewhere.

In the standard classical numerical presentation, K(x) is a total benchmark, but no terminating algorithm returns its exact value for all strings. Operationally, progressively better descriptions can lower an upper bound, while there is no computable stage at which one can know that the current bound is final for every input. The relation ${\mathrm{Short}}_{U}\left(x,b\right)$ is semidecidable; its complement is not, since semideciding both sides would decide the relation. This is the content used by the discovery and optimality barriers; the preceding subsection shows that it can be stated without taking a total *K*-function as primitive. The separate classical limit on formal certification is recorded below.

The same argument with the auxiliary tape carrying a fixed string *z* shows that x↦K(x∣z) is not computable. The conditional auxiliary result in Appendix D needs a separate argument because its conditioning string $z_{n}$ varies with the input length.

Theorem 2 uses the related fact that the set of *compressible* strings {x:K(x)<|x|} is undecidable. Were it decidable, then for each *n* one could compute the length-lexicographically first $x\in {\left\{0,1\right\}}^{n}$ with K(x)≥n, which exists by the counting argument of Appendix D.1. That *x* would then have the description “the first incompressible string of length *n*” of size O(logn), contradicting K(x)≥n for large *n*.

The arbitrary-threshold corollary in Appendix D then follows by specialization and requires no further classical input. Undecidability of the full threshold relation {(x,b):K(x)<b} is itself a consequence of the diagonal case through the computable reduction x↦(x,|x|). The reverse implication is invalid for an arbitrary diagonal slice, so the diagonal case is proved directly. The decision problem “is K(x)≤m?” is Turing-equivalent to the halting problem [5] (Ch. 2–3). Moreover, *K* is upper semicomputable: it can be approximated from above, with no computable bound on the rate of convergence [26]. Thus running a compressor gives an upper bound on K(x); there is no effective certification procedure that succeeds on every true claim that no shorter description exists.

The argument tolerates any fixed slack. For each fixed l∈N, the predicate K(x)<|x|−l is undecidable by the same construction: a decision procedure would let a short program output, for each *n*, the first string of length *n* that is *not ℓ*-compressible, describing a string of complexity at least n−l in O(logn) bits. The construction in fact excludes any decidable *separator*, that is, any test that accepts every string compressible by more than the margin and never accepts an incompressible one: if a decidable set *P* contains every *x* with K(x)<|x|−l and only *x* with K(x)<|x|, then the first length-*n* string outside *P* is computable from *n*, exists because incompressible strings lie outside *P*, and has K≥n−l while being describable in O(logn) bits. This is an independent classical strengthening. Proposition 2 instead uses the direct counting construction in Appendix D.6.

*Finite catalogs and the Busy Beaver stopping bound.* Fix the machine *U*, its output convention, and a step-counting semantics. Define

(A26) $$T_{U}\left(b\right):=\max\{{\mathrm{time}}_{U}\left(p\right):|p|\leq b,\;U\left(p\right)\;\mathrm{halts}\},$$

with value zero when the set is empty. A known upper bound on $T_{U}\left(b\right)$ suffices to classify halting by simulating all inputs through *b* bits for that many steps each. There is no total computable upper bound valid for every *b*, since one would decide halting for *U*. Andreev compares Busy Beaver functions defined through plain, prefix, and a priori complexity [25]. This bound supplies a sufficient exhaustive stopping rule, not the necessary work for deciding a particular output: a slow program may print a different string, or one with a much faster description.

More directly, no total computable function of *x* bounds the runtime of a fastest shortest program for *x*. If one did, simulating every program up to a known literal-description length bound for that many steps would identify a shortest program and compute K(x), contradicting Theorem A1. This also excludes a bound depending only on |x|; it does not give a numerical runtime lower bound for any specified short record.

Suppose halting has been completely classified through *b* bits and all halting outputs obtained. For any string represented in that finite catalog, its shortest listed program has length exactly K(x), because every shorter candidate is covered. An absent string has K(x)>b. Thus compressibility K(x)<|x| is decided for every |x|≤b+1, but exact complexity for every such string does not follow: some may require longer programs. For a particular candidate, one can sometimes exclude every shorter program as a description of *x* without settling all of those programs’ halting questions. Full halting classification is sufficient, not necessary.

Calude, Dinneen, and Shu report a complete halting classification through 84 program bits for their specified universal self-delimiting register machine, and 64 initial bits of its halting probability [88]. By the preceding argument, their catalog settles compressibility through 85 record bits under that output convention; it does not determine exact complexity for all those records. These cutoffs depend on the interpreter and are not a universal frontier. The obstruction is to an effective method extending such complete answers to all lengths, while individual cases and certified finite ranges can be settled.

### Appendix C.3. Certification Is Theory-Relative

A logically distinct limitation concerns formal certification. Let *F* be a computably axiomatized formal theory, strong enough to express the relevant statements about *U* and sound for them. There is a constant $c_{F,U}$, depending on *F* and *U*, such that *F* proves no statement K(x)>m with $m>c_{F,U}$ [5,76]. If *F* proved arbitrarily large lower bounds on *K*, a program could take a threshold *m*, enumerate the theorems of *F* until it found a proof that some string exceeded that threshold, and output that string: a description of a string of complexity greater than *m* in O(logm)+|F| bits, a contradiction for large *m*.

Since certifying that a program *p* is shortest for *x* requires ruling out every shorter description, such certificates are unavailable beyond a constant fixed by *F* and *U*. The constant is theory- and machine-dependent, not a universal physical scale. This classical limitation is separate from the micro-to-macro construction barriers of the main text; it is machine-checked as chaitin_certification_ceiling.

The statement ${\mathrm{Short}}_{U}\left(x,b\right)$ has a finite witness when true: exhibit a program shorter than *b* that prints *x*. Its negation says that every such candidate fails, and continued simulation by itself need never produce a finite certificate. In the usual classical presentation, the least threshold is packaged into the numerical quantity K(x); constructive formulations need not make that same move. The barrier results require only the stated limits on effective construction and certification. They do not require the additional philosophical claim that every proposition that no finite observer can establish must nevertheless correspond to an observer-independent mathematical or physical fact.

The incompleteness literature should be read with the same separation. The Gödel–Rosser theorem states, in the usual setting, that a consistent effectively axiomatized theory strong enough for elementary arithmetic has a sentence that is neither provable nor refutable in that theory [89]. This is a statement about derivability relative to a specified formal system. Calling an independent sentence “true but unprovable” adds a semantic evaluation in an intended model; that step is not supplied by syntactic independence alone. A stronger consistent effective theory may settle a particular sentence while remaining incomplete in turn. Chaitin’s certification ceiling has the same theory-relative character: changing *F* or the reference machine changes the constant, so the theorem rules out unbounded certification within one fixed formal system rather than every possible route to knowledge of an individual instance.

### Appendix C.4. Finite-State Boundedness, Compact Continua, and Recurrence

The finite-state argument used in Section 8 depends on effective finiteness, not on geometric boundedness. Let f:S→S be a computable deterministic transition on an effectively represented set with |S|=N<∞, and let membership in a target A⊆S be decidable. From any initial state $s_{0}$, simulation must either reach *A* or repeat a previously visited state within N+1 visits. If $s_{i}=s_{j}$ with i<j, determinism gives $s_{i+k}=s_{j+k}$ for every k≥0; the future is periodic from that point onward. Eventual reachability is, therefore, decidable by exact cycle detection. The value of *N* need not be supplied to the algorithm: decidable state equality and the promise of finiteness suffice for termination. A bounded-tape Turing machine is an example.

A compact positive-dimensional continuum is different. Compactness gives a finite cover at every fixed resolution ε>0, but no resolution-independent finite set of exact states. A simple example is an irrational rotation of the circle: every orbit returns arbitrarily close to its starting point but never returns exactly. More generally, Poincaré recurrence states, under the usual finite-measure and measure-preserving hypotheses, that almost every point returns infinitely often to neighborhoods of its initial state; Hamiltonian flows on finite-measure invariant regions fall under this framework because they preserve Liouville measure  [90]. Recurrence is, therefore, much weaker than finite-state periodicity. It supplies neither exact repetition nor, in general, a computable return-time bound that could serve as a negative reachability certificate.

A compact continuum can nevertheless carry an effectively unbounded symbolic state space inside a geometrically bounded region. Moore made this explicit using generalized shifts: the two halves of a Turing tape are encoded in the digits of a point in a Cantor set, and iteration updates those digits as a Turing machine updates its tape. He showed that smooth systems with as few as three degrees of freedom, including the motion of a particle in a three-dimensional potential, can realize universal computation  [52,55]. Approximate recurrence does not identify two computational states: points that agree to finite precision may differ in deeper tape digits and hence in their subsequent symbolic evolution. Continuity alone is not sufficient for universality; it merely removes the finite-state pigeonhole argument. The dynamics must organize the available fine distinctions into an effective simulation.

Compact conservative and fluid systems show that neither compactness nor conservation restores the finite-state conclusion. Cardona et al. realized a Turing-complete area-preserving return map as a stationary incompressible Euler flow on a suitably metrized $S^{3}$ [56]. Dyhr et al. construct Turing-complete stationary Navier–Stokes solutions on Hodge-admissible Riemannian three-manifolds after, in general, a non-small metric deformation; the resulting fields are harmonic and the undecidable predicate is Lagrangian reachability of a computably specified open target  [57]. The result, therefore, does not establish Turing completeness of time-dependent Euclidean Navier–Stokes evolution, undecidability of singularity formation, or blow-up. Tao’s averaged-equation blow-up theorem and proposed fluid logic program concern a different, time-dependent Eulerian direction  [58,59].

This mathematical distinction should not be confused with a claim about unlimited physical precision. Exact continuum encodings can use distinctions at progressively finer scales. If an operational physical theory instead has only finitely many distinguishable states with computable deterministic transitions, exact cycle detection restores reachability decidability. Noise or finite measurement precision may prevent an indefinitely stable realization of a continuum encoding without changing the exact mathematical theorem. Whether nature supplies the required fine-scale state capacity is, therefore, a separate physical question.

These continuum examples concern the answer-side undecidability barrier; the finite-record AIT construction barriers require no continuum resource and remain logically distinct, although one substrate may exhibit both. These are computability statements, not claims about whether every inaccessible value is an observer-independent property of the world.

## Appendix D. Proofs and Supporting Construction Results

This appendix records the counting argument behind Theorem 1 and closes four apparent loopholes in the construction barriers: unbounded substrates, a supplied length threshold, complete knowledge of the law, and a promise that open-ended search eventually succeeds. The final subsection gives the periodic-block counting construction and the full proof of Proposition 2 for reusable model-compressors.

### Appendix D.1. Counting Proof for Theorem 1

Fix a conditioning string *z* and write K(·∣z) for complexity relative to *U* with *z* on the auxiliary tape. There are $2^{m}-1$ binary strings of length strictly less than *m*, so there are at most $2^{m}-1$ candidate programs; restricting the machine domain to a prefix-free set can only reduce this count. Since a deterministic program has one output, fewer than $2^{m}$ strings possess a description shorter than *m*. Hence

(A27) $$\#\left\{y\in {\left\{0,1\right\}}^{n}:K\left(y|z\right)<m\right\}\;<\;2^{m}.$$

Setting m=n−d gives (6). Because *z* is fixed across the ensemble, the bound is independent of the chosen shared microlaw and observation map. It fails if the conditioning information varies with the initial state: when *z* contains *y*, K(y∣z)=O(1). The count itself needs no probability; reading it as a fraction of initial states uses the uniform counting measure, and a different prior on $\sigma_{0}$ gives a different fraction.

### Appendix D.2. Finite-Horizon Embeddings Transfer the Barriers

Let $F_{\mathrm{fin}}$ denote the class of finite micro-experiments in Definition 1. Let U be an effectively encoded class of systems whose state space or physical substrate may be unbounded, but where each input specifies a finite observation horizon and hence a finite binary record $x_{U}\left(u\right)$.

The main witnesses are finite, but an unbounded substrate family may contain them as finite-horizon instances. Their construction barriers survive such an enlargement whenever the embedding is computable and preserves the observed records.

**Corollary A2** (Inheritance under finite-horizon embeddings)**.**

*Suppose there is a computable map $e:F_{\mathrm{fin}}\to U$ satisfying*

(A28)
$$x_{U}\left(e\left(\mu \right)\right)=x\left(\mu \right)\;\mathrm{for}\;\mathrm{every}\;\mu \in F_{\mathrm{fin}}.$$

*Then the discovery and optimality barriers hold on U: no total computable procedure on U can satisfy the guarantee of Theorem 2, and no such procedure can satisfy the fixed-regret guarantee of Theorem 3, even on the embedded compressible records. If, on the embedded shift-register family, the conditioning string ${\tilde{z}}_{n}$ (the encoded embedded law, projection, horizon, and frame) depends on n but not on the initial state $y\in {\left\{0,1\right\}}^{n}$, then the residual-information bound also transfers:*

(A29)
$$\#\left\{y\in {\left\{0,1\right\}}^{n}:K\left(x_{U}\left(e\left(\mu_{n,y}\right)\right)|{\tilde{z}}_{n}\right)<n-d\right\}<2^{n-d}$$

*for every integer d with 0≤d≤n.*

**Proof.** For fixed *n*, Equation (A28) gives $x_{U}\left(e\left(\mu_{n,y}\right)\right)=y$. Since ${\tilde{z}}_{n}$ is fixed across the ensemble, the counting argument of Theorem 1 applies without change and gives Equation (A29). For the computational statements, suppose a total computable procedure with either claimed guarantee existed on U. Composing it with the computable map *e* would give a total computable procedure with the same validity and length guarantee on $F_{\mathrm{fin}}$, contradicting Theorem 2 or Theorem 3.    □

A standard instance adjoins an infinite inactive tape or lattice to a finite experiment, lets the original dynamics act on a marked finite region, and lets the observation map ignore the added degrees of freedom. The horizon remains finite and the record is unchanged. Thus an unbounded substrate class admitting this effective extension inherits the barriers. Set-theoretic inclusion alone is insufficient: the embedding must be computable and must preserve the observed record; the conditioning data in the residual-information ensemble must not encode the varying initial state.

### Appendix D.3. A Supplied Length Threshold Does Not Restore Construction

A target code length does not provide a program meeting that target or a stopping rule for deciding that none exists. The threshold formulation, therefore, retains the discovery obstruction.

**Corollary A3** (Arbitrary thresholds)**.**

*There is no total computable procedure B that, given a finite string x and threshold b∈N, always returns a program B(x,b) with U(B(x,b))=x and is guaranteed to satisfy K(x)<b⇒|B(x,b)|<b.*

**Proof.** If such a *B* existed, then on input *x* one could compute p=B(x,|x|). Validity gives |p|<|x|⇒K(x)<|x|, while the guarantee gives the converse. Comparing the two lengths would decide whether K(x)<|x|, contradicting Appendix C.    □

Thus supplying a target code length in advance does not restore a uniform guarantee. This result is an immediate threshold variant of the discovery obstruction, not a separate construction barrier.

### Appendix D.4. Known Laws Do Not Make Conditional Complexity Computable

Supplying the common microlaw, observation map, horizon, and frame changes the benchmark from unconditional to conditional complexity. It does not make that benchmark computable when the same procedure must work across the growing family.

**Corollary A4** (Conditional form)**.**

*There is no total computable procedure that, for every nonempty string y, returns $K(y|z_{\left|y\right|})$, where $z_{n}=\left(D_{n},\rho_{n},n,C\right)$ is the common microlaw, observation map, register length (which fixes the horizon n−1), and frame of Theorem 1. Conditioning on exactly known laws does not restore computability.*

**Proof.** Suppose $g\left(y\right)=K\left(y|z_{\left|y\right|}\right)$ were total computable. On input *n*, enumerate ${\left\{0,1\right\}}^{n}$ and return the first $y_{n}$ with $g(y_{n})\geq n$. Such a string exists by the counting bound of Appendix D.1. Given $z_{n}$, a program of constant size can recover *n* and run this search. Hence $K\left(y_{n}|z_{n}\right)=O\left(1\right)$, contradicting $g(y_{n})\geq n$ for large *n*.    □

The varying condition $z_{n}$ is why this statement needs its own diagonal argument rather than the standard theorem for one fixed conditioning string.

### Appendix D.5. Open-Ended Discovery Has No Computable Schedule

Let S(x,b) denote the open-ended search that enumerates all programs shorter than *b*, runs them dovetailed, and returns the first that outputs *x*.

On a positive instance, this search eventually succeeds. Turning that fact into a constructor that terminates on all inputs would require a computable deadline. No such uniform deadline exists.

**Proposition A2** (No computable schedule)**.**

*There is no total computable function τ such that, for every pair (x,b) with K(x)<b, the search S(x,b) returns a program within τ(x,b) computation steps.*

**Proof.** Suppose such a τ existed. Run S(x,b) for τ(x,b) steps and report whether a program has been returned. If K(x)<b, the search returns within the allotted time; if K(x)≥b, no program shorter than *b* outputs *x*. This would decide the threshold relation, and, in particular, its diagonal case K(x)<|x|, contradicting Appendix C.    □

Hence a valid promise K(x)<b guarantees eventual discovery without supplying a computable universal deadline.

### Appendix D.6. Periodic Observation Blocks: A Counting Proof

This construction proves Proposition 2 directly. It uses the same counting idea as Proposition 1, with the observation count supplied to the decoder and a reusable code that remains valid outside the constructed stream.

*Domain, objective, and shared information.* Fix a chunk width b≥1, frame *C*, and positive total margins $\delta_{\mathrm{comp}},\delta_{\mathrm{test}}$. A block $v=\left(Y_{1},\ldots ,Y_{m}\right)\in {\left({\left\{0,1\right\}}^{b}\right)}^{m}$ contains bm bits. Its stream repeats that block indefinitely; construction uses its first *m* chunks and testing uses the next *m*, obtained only after the pair has been selected. The objective is faithful reproduction of the designated chunks, measured by

(A30) $$\mathrm{OF}\left(\hat{Y};Y\right)=\sum_{i=1}^{m}1\left\{{\hat{Y}}_{i}\neq Y_{i}\right\},$$

with the required discrepancy being zero. It can be evaluated on the output of *M* at the queried phases. The representation is supplied: $D_{i}=Y_{i}$ and ρ=id. It retains every distinction required by this objective, and there is no omitted residual to provide a saving. This is a permitted fixed-representation subclass of the joint problem, without an auxiliary discarded bit.

The decoder is given *C*, *b*, *m*, the queried block length, and the starting phase. These are common across all $2^{bm}$ blocks at fixed *m*; the data *v* are not supplied. A complete code includes the acquired model, the representation if not already supplied, the joint parameter code, and all framing. The baseline is literal concatenation, of length bm on each construction or test block. At supplied length it is a valid fixed-length code. During testing it continues to ignore past data, although those data may be available to both codes. This baseline choice is declared before selection and does not change with the returned model.

The stream also has a finite microscopic realization: store *v* in a cyclic register, advance by *b* bits per step, and read its first *b* bits. The update and readout conventions are fixed, with *m* specifying the register length. A horizon H=m−1 yields one block. If the constructor’s input includes a complete micro-experiment, construct this specification effectively from *v*; it remains input to the constructor, not free information for the returned code’s decoder.

**Proof of Proposition** **2.** Fix a total computable constructor *A* satisfying the proposition’s validity condition. For each *m* and $v\in {\left({\left\{0,1\right\}}^{b}\right)}^{m}$, run *A* on the corresponding instance and run its returned encoder on *v*. Let $q_{A}\left(v;m\right)$ be the resulting complete code, under the fixed convention above. This is a total computable map: both *A* and every returned encoder terminate on their declared inputs. A single fixed decoder reconstructs *v* from $q_{A}\left(v;m\right)$ and the common supplied conditions. Consequently, for fixed *m*, the map $v\mapsto q_{A}\left(v;m\right)$ is injective. Since

(A31) $$\left|\{q\in {\left\{0,1\right\}}^{*}:|q|<bm\}\right|=\sum_{j=0}^{bm-1}2^{j}=2^{bm}-1,$$

there is at least one *v* with $|q_{A}\left(v;m\right)|\geq bm$. Let $v_{m}$ be the first such block in lexicographic order. A finite search computes $v_{m}$ from *m*: enumerate the blocks, compute each $q_{A}\left(v;m\right)$, and stop at the first whose length is at least bm. Only code lengths are tested; the search need not decide equivalence, totality, or optimality of arbitrary programs. Its termination follows from the stipulated totality and validity of *A*, and from the counting argument. Construct one model $M_{A}$ containing *A* and this search. Given *m* and a queried phase, it computes $v_{m}$ and generates the requested finite segment of its periodic repetition. Its encoder compares the observed segment with that prediction. It emits a one-bit match code 0 if they agree; otherwise it emits 1 followed by the literal segment. The decoder regenerates the prediction in the first case and reads the supplied number of literal bits in the second. At every fixed supplied block length, the code set consisting of 0 and all strings 1z of the appropriate literal length is prefix-free. Both maps are total for every finite segment. This is a stable phase-and-correction interface: the model carries the recurring pattern, phase is a supplied condition, and the code records whether corrections are needed. The escape case preserves validity even when the observed stream does not follow the prediction. Let $c_{A}$ bound the actual complete construction-code length of this pair on its matching block, including the model program, its fixed framing, and the match bit. This is a constant in *m*; it includes the cost of describing *A*. The supplied identity representation has zero incremental cost. Thus, on the first block $v_{m}$ and the later block from the same periodic stream,

(A32) $$L_{\mathrm{keep}}\left(v_{m};\mathrm{id},M_{A}|u_{m},C\right)\leq c_{A},\;L_{M_{A}}\left(\theta_{\mathrm{test}}|u_{\mathrm{test}},h_{m},C\right)=1.$$

The model meets relevance and reuse throughout the declared domain. For every *m* large enough that $bm\geq c_{A}+\delta_{\mathrm{comp}}$ and $bm\geq 1+\delta_{\mathrm{test}}$, it also meets both compression inequalities of Definition 3. The test block is not used to select the model: by construction, the continuing stream supplies the predicted block afterward. The code returned by *A* has length at least bm and fails (iii), proving (a). Let $L^{*}\left(v_{m}\right)$ be the best qualifying complete construction-code length on this instance and its declared continuation. Since $L^{*}\left(v_{m}\right)\leq c_{A}$, part (b) follows from the growing lower bound

(A33) $$|q_{A}\left(v_{m};m\right)|-L^{*}\left(v_{m}\right)\geq bm-c_{A}.$$

   □

The fixed constant uses the fact that *m* is supplied. If the decoder must also learn *m*, encode it and include that length; the corresponding comparison gains the cost of describing *m*. The search inside $M_{A}$ can take an enormous amount of time, so the result supplies no practical runtime bound for a particular short block. Nor does it say that learning recurrence is impossible: after receiving more copies, a method that stores and reuses $v_{m}$ may meet the certificate. The proved failure is on the available construction block, where a compact qualifying model already exists. A universal joint constructor must handle this supplied-representation subclass, but the argument makes no separate claim about the difficulty of selecting ρ.

## Appendix E. Composition Links Construction and Answer Barriers

This appendix records the closure observation represented by the dashed implication in Figure 7. Construction and evaluation are different tasks, but a pipeline that performs both is itself an algorithm from the microscopic input to the macroscopic answer. If that answer map is noncomputable, at least one stage of every proposed universal pipeline must fail to be total or correct. This is not an additional source of undecidability. Its role is to state the one-way dependence between the answer barrier and a constructor–evaluator pipeline.

To state the argument, fix an effectively encoded family F of microscopic specifications, a projection $\rho_{*}$, and one macroscopic question $q_{*}$. The formal semantics of the problem assigns each μ∈F a finite answer in an effectively encoded set Y. Write this declared answer map as

(A34) $$a_{*}:F⟶Y.$$

For example, $q_{*}$ may ask whether the $\rho_{*}$-projected trajectory ever enters a target set. Over an open-ended time domain, the resulting binary map can encode the halting problem. Totality of $a_{*}$ is part of the formal problem specification; the proposition does not derive it from a claim about observer-independent physical truth.

Suppose a total computable constructor $A_{*}$ turns every microscopic input into a finite model code, and a total computable evaluator $E_{*}$ extracts the answer to $q_{*}$ from that code. The proposed detour is the composition $E_{*}\circ A_{*}$.

**Proposition A3** (Composition rule for a fixed macroquestion)**.**

*Let $A_{*}:F\to {\left\{0,1\right\}}^{*}$ and $E_{*}:{\left\{0,1\right\}}^{*}\to Y$ be total computable maps. If*

(A35)
$$E_{*}\left(A_{*}\left(\mu \right)\right)=a_{*}\left(\mu \right)\;\mathrm{for}\;\mathrm{every}\;\mu \in F,$$

*then $a_{*}$ is computable. Consequently, if $a_{*}$ is noncomputable, no such total and universally correct constructor–evaluator pair exists.*

**Proof.** Given μ, first compute $A_{*}\left(\mu \right)$ and then $E_{*}\left(A_{*}\left(\mu \right)\right)$. The assumed equality makes this composition an algorithm for $a_{*}$. The final statement is the contrapositive.    □

The proposition concerns a uniform pair that works for the entire family F. It permits a useful model for an individual μ, and it says nothing about whether a model compresses, transfers to new data, or answers questions outside $q_{*}$. It also separates the two algorithms only for interpretation: the impossibility applies to their composition, not necessarily to either stage considered alone.

The same reasoning applies to a scheme advertised as correct for varying projections and questions. Fixing any admissible $\left(\rho_{*},q_{*}\right)$ produces the factorization in Proposition A3. Holding one admissible question and representation fixed gives a slice of any scheme that promises to handle them all; a single noncomputable slice therefore rules out such a scheme.

*The converse is only an encoding fact.* At the fixed-task level, a computable answer map always has a trivial factorization: $A_{*}$ can encode the entire microscopic input μ, after which $E_{*}$ decodes it and runs the algorithm for $a_{*}$. This “model” need not compress the input, transfer to new data, or expose reusable macroscopic structure. Computability of the answer is, therefore, not sufficient for algorithmic emergence.

The fixed coarse-graining is an essential hypothesis. If a constructor may choose ρ without an independent relevance constraint, it can avoid the hard question by discarding the distinction the question asks about. The obstruction applies when ρ is supplied, when the constructor must work for every admissible ρ, or when the relevance criterion requires the selected question to be preserved.

## Appendix F. Machine-Checked Formalization

This appendix records the declaration-level correspondence between the paper and the Lean 4 development. BCOM WP0195 gives the full inventory and axiom audit.

### Appendix F.1. Main Barriers and Finite-Horizon Inheritance

The declarations named below occur in the KTAIT module AlgorithmicEmergence.lean. Theorem 1 is proved outright (counting_bound, few_low_complexity_strings, most_states_incompressible). Theorem 2 corresponds to no_universal_compressor_micro; the generic string reductions are available as no_universal_compressor and no_compression_improver_of_raw. The Lean statement of the discovery reduction is formulated abstractly over any computable faithful embedding of finite strings into experiments, that is, any emb with x(emb(y))=y; the trivial identity/full-readout construction of Equation (7) used in the main text, and the shift-register family of Theorem 1, are both instances of that hypothesis. The machine-checked result therefore does not depend on the particular witness chosen for exposition, and the paper’s choice makes explicit that the computational result requires only arbitrary-record representability. Corollary A2 uses the same abstract embedding: its residual-information part is counting_bound applied with the shared embedded condition, while its discovery and optimality parts are, respectively, no_universal_compressor_micro and no_additive_approximation_compressible. Theorem 3, in the compressible-witness form stated here, corresponds to no_additive_approximation_compressible, whose classical input is the padded-witness strengthening of non-approximability; the weaker unconditional statement is no_additive_approximation, and identification_barrier records the exact-optimality (c=0) consequence. Theorem 3 and Corollary 1 are derived here from Proposition 1; the Lean statements and their hypotheses are unaffected. Proposition 1 and Theorem A2 are proved at paper level only; no machine-checked declarations are claimed for them.

### Appendix F.2. Relational, Auxiliary, and Fixed-Question Results

Corollary A1, the witness-based form of the optimality barrier, is machine-checked as relational_optimality_barrier. Its analytic inputs are named hypotheses in the same style as elsewhere: LengthUnbounded for the counting argument that a valid constructor’s returned length grows along the witness family, CompetitorBound and BoundOutrun for the competitor being describable from the search index, and WitnessCompresses for the padded compression witness. No shortest description appears in that statement or its proof. The current Lean development supplies *K* through its abstract AIT frame rather than constructing a global total *K*-function; theorem statements quantify over frames carrying that benchmark. Thus the foundational commitment is explicit in the formal interface rather than hidden in the proofs.

The threshold, conditional, and schedule results of Appendix D are machine-checked. compressor_of_threshold and threshold_undecidable_of_compressible cover the threshold specialization. conditional_complexity_uncomputable covers the varying-condition corollary. no_computable_schedule covers Proposition A2, with the step-indexed search and promise-halting exposed as named hypotheses. Proposition A3 is machine-checked as fixed_task_computable_of_factorization. The varying-projection result is query_slice_computable_of_query_complete. Its nonexistence consequence is no_query_complete_of_noncomputable_slice. The extensional converse discussed after the proposition is fixed_task_computable_iff_factorization; its identity construction uses the microscopic input as its own model code. These declarations are proved from Mathlib’s computability interface and consume no AIT hypothesis.

### Appendix F.3. Reusable-Model Acquisition

The revised Proposition 2 and its periodic-block construction in Appendix D.6 are proved at paper level. They are not claimed to be covered by the existing declarations no_universal_emergence_constructor and no_bounded_regret_emergence_constructor. Those declarations express the earlier abstract transfer through program-to-model and model-to-program translations under named hypotheses; their validity is independent of this change in the manuscript.

The revised correspondence requires the joint-code validity and injectivity argument, finite search for a missed block, a fixed program implementing that search with the block length supplied, and the match/escape code’s satisfaction of the acquisition and prospective margins. The queue records these obligations. Until they are formalized and the correspondence checked, the revised proposition carries a paper-level annotation. The record-level declarations described above and the functional-core benchmarks below retain their existing scope.

### Appendix F.4. Functional-Core Benchmarks

The module FunctionalCore.lean uses the namespace KTAIT.FunctionalCore. It defines the conditional exact and approximate benchmarks as functionalCoreComplexity and approximateCoreComplexity. A shortest representative is expressed by IsFunctionalCore; its existence from a supplied finite realization is exists_functional_core. The bound in Equation (A7) has a typed counterpart, functional_core_le_implementation, under explicit hypotheses that an interpreter realizes the declared behavior within a fixed description overhead. These results concern minimum description length for fixed behavior; they assert neither computable extraction nor optimal predictive quality or runtime. The probability argument in Proposition A1 is proved at paper level.

### Appendix F.5. Scope of the Formalization

The conservation/coarse-graining equations in Section 6 do not introduce a separate theorem suite. Reversible complete-state invariance and the binary localization balance are direct applications of the already machine-checked localization machinery in the companion development; Equation (46) additionally uses only the standard non-growth of Kolmogorov complexity under a fixed computable projection. We, therefore, state these as consequences rather than new named theorems.

The finite-catalog and runtime Busy Beaver discussion in Appendix C.2 is likewise at paper level. Semidecidability of K(x)<b is not formalized: it would require an operational machine semantics and a dovetailing construction, which the present development lacks. No main theorem depends on it: Theorem 2 rules out a *total* procedure that halts on every input and shortens whenever a shortening exists, and semidecidability is the complementary positive fact about partial search. Its negative scheduling companion is formalized: no_computable_schedule treats the search abstractly, as a step-indexed function with soundness and promise-halting as hypotheses, so the auxiliary Proposition A2 is machine-checked without the dovetailer being built.
